# Efficacy and Safety of COVID-19 Convalescent Plasma in Hospitalized Patients: A Randomized Clinical Trial

**DOI:** 10.1001/jamainternmed.2021.6850

**Published:** 2021-12-13

**Authors:** Mila B. Ortigoza, Hyunah Yoon, Keith S. Goldfeld, Andrea B. Troxel, Johanna P. Daily, Yinxiang Wu, Yi Li, Danni Wu, Gia F. Cobb, Gillian Baptiste, Mary O’Keeffe, Marilou O. Corpuz, Luis Ostrosky-Zeichner, Amee Amin, Ioannis M. Zacharioudakis, Dushyantha T. Jayaweera, Yanyun Wu, Julie V. Philley, Megan S. Devine, Mahalia S. Desruisseaux, Alessandro D. Santin, Shweta Anjan, Reeba Mathew, Bela Patel, Masayuki Nigo, Rabi Upadhyay, Tania Kupferman, Andrew N. Dentino, Rahul Nanchal, Christian A. Merlo, David N. Hager, Kartik Chandran, Jonathan R. Lai, Johanna Rivera, Chowdhury R. Bikash, Gorka Lasso, Timothy P. Hilbert, Monika Paroder, Andrea A. Asencio, Mengling Liu, Eva Petkova, Alexander Bragat, Reza Shaker, David D. McPherson, Ralph L. Sacco, Marla J. Keller, Corita R. Grudzen, Judith S. Hochman, Liise-anne Pirofski, and the CONTAIN COVID-19 Consortium for the CONTAIN COVID-19 Study Group, Lalitha Parameswaran, Anthony T. Corcoran, Abhinav Rohatgi, Marta W. Wronska, Xinyuan Wu, Ranjini Srinivasan, Fang-Ming Deng, Thomas D. Filardo, Jay Pendse, Simone B. Blaser, Olga Whyte, Jacqueline M. Gallagher, Ololade E. Thomas, Danibel Ramos, Caroline L. Sturm-Reganato, Charlotte C. Fong, Ivy M. Daus, Arianne Gisselle Payoen, Joseph T. Chiofolo, Mark T. Friedman, Ding Wen Wu, Jessica L. Jacobson, Jeffrey G. Schneider, Uzma N. Sarwar, Henry E. Wang, Ryan M. Huebinger, Goutham Dronavalli, Yu Bai, Carolyn Z. Grimes, Karen W. Eldin, Virginia E Umana, Jessica G. Martin, Timothy R. Heath, Fatimah O. Bello, Daru Lane Ransford, Maudry Laurent-Rolle, Sheela V. Shenoi, Oscar Bate Akide-Ndunge, Bipin Thapa, Jennifer L. Peterson, Kelly Knauf, Shivani U. Patel, Laura L. Cheney, Christopher A. Tormey, Jeanne E. Hendrickson

**Affiliations:** 1 Division of Infectious Disease, Department of Medicine, NYU Grossman School of Medicine, New York, New York; 2 Department of Microbiology, NYU Grossman School of Medicine, New York, New York; 3 Division of Infectious Disease, Department of Medicine, Albert Einstein College of Medicine, Montefiore Medical Center, Bronx, New York; 4 Department of Population Health, NYU Grossman School of Medicine, New York, New York; 5 Department of Microbiology and Immunology, Albert Einstein College of Medicine, Bronx, New York; 6 Department of Medicine, NYU Grossman School of Medicine, New York, New York; 7 Department of Surgery, NYU Grossman School of Medicine, New York, New York; 8 Department of Medicine, NYU Long Island School of Medicine, Mineola, New York; 9 Division of Infectious Disease, Department of Internal Medicine, The University of Texas Health Science Center at Houston, McGovern Medical School, Houston; 10 Department of Emergency Medicine, The University of Texas Health Science Center at Houston, McGovern Medical School, Houston; 11 Division of Infectious Disease, Department of Medicine, University of Miami Miller School of Medicine, Miami, Florida; 12 Miami Clinical and Translational Science Institute, University of Miami Miller School of Medicine Miami, Florida; 13 Department of Pathology, University of Miami Miller School of Medicine, Miami, Florida; 14 Division of Pulmonary and Critical Care Medicine, Department of Internal Medicine, The University of Texas Health Science Center at Tyler, UTHealth East Texas, Tyler; 15 Section of Infectious Diseases, Department of Internal Medicine, Yale University School of Medicine, New Haven, Connecticut; 16 Department of Obstetrics, Gynecology, and Reproductive Sciences, Yale University School of Medicine, New Haven, Connecticut; 17 Division of Critical Care, Department of Internal Medicine, The University of Texas Health Science Center at Houston, McGovern Medical School, Houston; 18 Laura and Isaac Perlmutter Cancer Center, NYU Grossman School of Medicine, New York, New York; 19 Department of Internal Medicine, The University of Texas Rio Grande Valley, Edinburg; 20 Division of Pulmonary, Critical Care, and Sleep Medicine, Department of Medicine, Medical College of Wisconsin, Milwaukee; 21 Division of Pulmonary and Critical Care Medicine, Department of Medicine, Johns Hopkins University, Baltimore, Maryland; 22 Department of Biochemistry, Albert Einstein College of Medicine, Bronx, New York; 23 Department of Pathology, NYU Grossman School of Medicine, New York, New York; 24 Department of Pathology, Albert Einstein College of Medicine, Bronx, New York; 25 Department of Environmental Health, NYU Grossman School of Medicine, New York, New York; 26 Department of Child and Adolescent Psychiatry, NYU Grossman School of Medicine, New York; 27 Nathan S. Kline Institute for Psychiatric Research, Orangeburg, New York; 28 Clinical Research Information Technology and Informatics, NYU Grossman School of Medicine, New York, New York; 29 Clinical and Translational Science Institute of Southern Wisconsin, Medical College of Wisconsin Milwaukee; 30 Center for Clinical and Translational Sciences, Division of Cardiovascular Medicine, Department of Internal Medicine, The University of Texas Health Science Center at Houston, McGovern Medical School, Houston; 31 Harold and Muriel Block Institute for Clinical and Translational Research, Albert Einstein College of Medicine and Montefiore Medical Center Bronx, New York; 32 Ronald O. Perelman Department of Emergency Medicine, NYU Grossman School of Medicine, New York, New York; 33 NYC Health and Hospitals Corporation Clinical and Translational Science Institute, NYU Grossman School of Medicine, New York, New York; 34 Leon H. Charney Division of Cardiology, Department of Medicine, NYU Grossman School of Medicine, New York, New York; 35 Department of Urology, NYU Long Island School of Medicine, Mineola, New York; 36 Department of Pediatrics, NYU Grossman School of Medicine, New York, New York; 37 Department of Pathology, NYU Long Island School of Medicine, Mineola, New York; 38 Pfizer Vaccine Clinical Research and Development, Pfizer Inc, Pearl River, New York; 39 Department of Emergency Medicine, The Ohio State University, Ohio; 40 Department of Pathology and Laboratory Medicine, The University of Texas Health Science Center at Houston, McGovern Medical School, Houston; 41 Department of Medicine, Medical College of Wisconsin, Milwaukee; 42 Department of Laboratory Medicine, Yale University School of Medicine, New Haven, Connecticut; 43 Department of Pediatrics, Yale University School of Medicine, New Haven, Connecticut

## Abstract

**Importance:**

There is clinical equipoise for COVID-19 convalescent plasma (CCP) use in patients hospitalized with COVID-19.

**Objective:**

To determine the safety and efficacy of CCP compared with placebo in hospitalized patients with COVID-19 receiving noninvasive supplemental oxygen.

**Design, Setting, and Participants:**

CONTAIN COVID-19, a randomized, double-blind, placebo-controlled trial of CCP in hospitalized adults with COVID-19, was conducted at 21 US hospitals from April 17, 2020, to March 15, 2021. The trial enrolled 941 participants who were hospitalized for 3 or less days or presented 7 or less days after symptom onset and required noninvasive oxygen supplementation.

**Interventions:**

A unit of approximately 250 mL of CCP or equivalent volume of placebo (normal saline).

**Main Outcomes and Measures:**

The primary outcome was participant scores on the 11-point World Health Organization (WHO) Ordinal Scale for Clinical Improvement on day 14 after randomization; the secondary outcome was WHO scores determined on day 28. Subgroups were analyzed with respect to age, baseline WHO score, concomitant medications, symptom duration, CCP SARS-CoV-2 titer, baseline SARS-CoV-2 serostatus, and enrollment quarter. Outcomes were analyzed using a bayesian proportional cumulative odds model. Efficacy of CCP was defined as a cumulative adjusted odds ratio (cOR) less than 1 and a clinically meaningful effect as cOR less than 0.8.

**Results:**

Of 941 participants randomized (473 to placebo and 468 to CCP), 556 were men (59.1%); median age was 63 years (IQR, 52-73); 373 (39.6%) were Hispanic and 132 (14.0%) were non-Hispanic Black. The cOR for the primary outcome adjusted for site, baseline risk, WHO score, age, sex, and symptom duration was 0.94 (95% credible interval [CrI], 0.75-1.18) with posterior probability (P[cOR<1] = 72%); the cOR for the secondary adjusted outcome was 0.92 (95% CrI, 0.74-1.16; P[cOR<1] = 76%). Exploratory subgroup analyses suggested heterogeneity of treatment effect: at day 28, cORs were 0.72 (95% CrI, 0.46-1.13; P[cOR<1] = 93%) for participants enrolled in April-June 2020 and 0.65 (95% CrI, 0.41 to 1.02; P[cOR<1] = 97%) for those not receiving remdesivir and not receiving corticosteroids at randomization. Median CCP SARS-CoV-2 neutralizing titer used in April to June 2020 was 1:175 (IQR, 76-379). Any adverse events (excluding transfusion reactions) were reported for 39 (8.2%) placebo recipients and 44 (9.4%) CCP recipients (*P* = .57). Transfusion reactions occurred in 2 (0.4) placebo recipients and 8 (1.7) CCP recipients (*P* = .06).

**Conclusions and Relevance:**

In this trial, CCP did not meet the prespecified primary and secondary outcomes for CCP efficacy. However, high-titer CCP may have benefited participants early in the pandemic when remdesivir and corticosteroids were not in use.

**Trial Registration:**

ClinicalTrials.gov Identifier: NCT04364737

## Key Points

**Question:**

Does COVID-19 convalescent plasma (CCP), compared with placebo, improve the clinical status of hospitalized patients with COVID-19 requiring noninvasive supplemental oxygen?

**Findings:**

In this randomized clinical trial including 941 patients, based on the World Health Organization 11-point Ordinal Scale for Clinical Improvement, CCP did not benefit 468 participants randomized to CCP compared with 473 randomized to placebo from April 2020 to March 2021. However, in exploratory analyses, CCP appeared to benefit those enrolled from April to June 2020, the period when most participants received high-titer CCP and were not receiving remdesivir and corticosteroids at randomization.

**Meaning:**

In this trial, CCP did not meet prespecified outcomes for efficacy, but high-titer CCP may have benefited hospitalized patients with COVID-19 early in the pandemic when other treatments were not in use, suggesting a heterogenous treatment effect over time.

## Teaser

This randomized clinical trial examines the use of convalescent plasma from patients with COVID-19 in hospitalized patients with COVID-19 who are not receiving invasive oxygenation treatment.

## Introduction

First reported in December 2019,^1^ the COVID-19 pandemic spread to the US, with an epicenter in New York City (NYC) resulting in 203 000 cases and 18 600 fatalities from March to June 2020.^2^ The absence of effective therapies prompted COVID-19 convalescent plasma (CCP) use because of biological plausibility and historical success of convalescent plasma in prior pandemics^3,4,5^ and randomized trials for diphtheria^6,7^ and Argentine hemorrhagic fever.^8^ Although early CCP treatment of hospitalized patients with COVID-19 reduced mortality in matched-control studies,^9,10,11,12^ randomized clinical trials have yielded mixed results, reducing mortality in one study^13^ but not others,^14,15,16,17,18,19^ despite showing signals of efficacy in some subgroups.

On April 17, 2020, we initiated a randomized, double-blind, placebo-controlled trial of CCP vs normal saline in hospitalized patients with COVID-19 in NYC and Long Island, New York, requiring noninvasive oxygen supplementation. When the spring 2020 COVID-19 wave abated in NYC sites, the trial expanded to other regions in the US and continued until March 15, 2021.

## Methods

### Trial Design and Oversight

CONTAIN COVID-19 was an investigator-initiated, multicenter, randomized, double-blind, placebo-controlled trial comparing CCP with normal saline in hospitalized patients with laboratory-confirmed COVID-19 who required noninvasive oxygen supplementation. Participants were enrolled from April 17, 2020, to March 15, 2021, at 21 hospitals at 7 centers in Manhattan, Bronx, Brooklyn, and Long Island, New York; New Haven, Connecticut; Miami, Florida; Houston and Tyler, Texas; Baltimore, Maryland; and Milwaukee, Wisconsin. The institutional review boards of each participating center approved the study. The New York University CONTAIN Coordinating Center and Data Safety Monitoring Board (DSMB) provided trial oversight. Patients or legally authorized representatives provided either written or witnessed oral informed consent for participation in accordance with institutional review board–approved consent procedures. The trial protocol is available in Supplement 1.

### Patient Population

Eligible patients were adults aged 18 years or older hospitalized for 3 days or less or with symptoms of respiratory illness for 7 days or less (to include patients with presumably early phases of disease) who required noninvasive oxygen supplementation and had a positive nasopharyngeal SARS-CoV-2 reverse-transcriptase polymerase-chain-reaction test. Exclusion criteria were receipt of pooled immunoglobulin in the preceding 30 days, contraindication to transfusion, invasive mechanical ventilation or extracorporeal membrane oxygenation, volume overload, considered unlikely to survive past 72 hours based on investigator assessment, and receipt of a COVID-19 vaccine (after vaccines were available). Patients whose clinical outcomes were deemed not assessable after hospital discharge were also excluded. Race and ethnicity data were obtained from entries in the medical record, as reported by the participants, using fixed categories. Race and ethnicity data were included to provide additional information about participants included in the study and the potential generalizability of the results.

### Randomization and Risk Stratification

A centralized electronic system was used to randomly assign enrolled patients to receive CCP or placebo in a 1:1 ratio stratified by enrollment site and risk status using randomization block sizes of 4 and 6 to maintain balanced group sizes. Allocation was concealed. Patients, treating clinicians, trial personnel, and outcome assessors were blinded to group assignment. Patients were stratified as high or average risk for COVID-19 progression. High-risk participants were aged 60 years or older or younger than 60 years with at least 1 of the following criteria: chronic pulmonary or heart conditions, hypertension, chronic kidney disease, body mass index greater than or equal to 35 (calculated as weight in kilograms divided by height in meters squared), diabetes, or immunosuppression.^20^ Average risk participants were younger than 60 years without any high-risk condition (Supplement 1 and eMethods in Supplement 2).

### Trial Interventions

One unit of CCP (approximately 250 mL) was infused within 24 hours of randomization at a rate of less than or equal to 500 mL/h. From April 2020 to January 2021, participants at Montefiore Medical Center received CCP from donors who participated in the Montefiore COVID-19 convalescent plasma donor program.^21,22,23^ Because CCP could not be transferred between institutions, all other sites used CCP from New York Blood Center donors with a reactive anti-SARS-CoV-2 antibody test on the SARS-CoV-2 Microsphere Immunoassay.^24^ Criteria for high-titer CCP were not available in April 2020. From January 2021 onward, all sites used CCP qualified by the New York Blood Center as high titer by a signal to cutoff value greater than or equal to 12 on the Ortho-Clinical Diagnostics VITROS Anti-SARS-CoV-2 immunoglobulin G (IgG) platform.^25^ Placebo recipients received normal saline of equivalent volume. The trial product was masked with an opaque covering to ensure blinding of treating clinicians, research staff, and participants. The CCP SARS-CoV-2 spike protein IgG titers were determined retrospectively (eMethods in Supplement 2).

### Outcomes

The primary outcome was clinical status based on the participant scores on the 11-point WHO Ordinal Scale for Clinical Improvement (WHO scale)^26^ 14 days after randomization; the secondary outcome was clinical status on the scale 28 days after randomization. WHO scale scores range from 0 to 10, with 0 indicating uninfected and no viral RNA detected and 10 indicating dead. Mortality at 14 and 28 days after randomization was a tertiary outcome (eMethods in Supplement 2).

### Subgroup Analyses

The following exploratory analyses were proposed in the protocol: (1) CCP and participant plasma SARS-CoV-2 Spike Protein binding antibody titer and neutralizing titer, (2) CCP and participant SARS-CoV-2 antibody profiles and functional assays, (3) rates, levels and duration of SARS-CoV-2 RNA in nasopharyngeal swabs, (4) SARS-CoV-2 variants, (5) clinical status at other visit days, mortality and rates of discharge, (6) lymphocytes, neutrophils, and cytokines, and (7) moderating effect of concomitant medications—including corticosteroids, remdesivir, and anticoagulants on CCP effects. Studies 2 through 6 are not reported because they have not been completed. Analyses 1 and 7 were prespecified as exploratory. We report CCP and participant plasma antibody titers (analysis 1) and effects of corticosteroids and remdesivir, which became standard of care during the study (analysis 7), because of their explanatory power and the insights they provide into the primary outcome. Prespecified subgroup analyses were conducted for the following characteristics at randomization: age, WHO score, symptom duration, concomitant medications, CCP SARS-CoV-2 titer, and pretransfusion plasma SARS-CoV-2 IgG serostatus. Post hoc analysis was conducted to evaluate treatment effects over time.

Adverse events were systematically collected between randomization and study end point, including occurrence of transfusion-related acute lung injury, transfusion-associated circulatory overload, and other allergic reactions.

### Participant and CCP SARS-CoV-2 Spike Protein IgG and CCP Neutralizing Titers

The CCP and pretransfusion participant plasma SARS-CoV-2 IgG titers were determined retrospectively using single plate and automated Spike ectodomain protein enzyme-linked immunosorbent assays^21,27,28^ and reported as half-maximal effective concentrations (EC_50_). A participant plasma SARS-CoV-2 IgG EC_50_ value less than 1:100 was considered seronegative. COVID-19 convalescent plasma SARS-CoV-2 IgG titers were categorized as low and high EC_50_ (CCP EC_50_) for analysis, dichotomized at the median EC_50_ (eMethods in Supplement 2). COVID-19 convalescent plasma–neutralizing titers were determined via a vesicular stomatitis pseudovirus assay (Q2) as described.^22^

### Stopping the Trial

The DSMB conducted interim analyses every 2 to 4 weeks. The statistical analysis plan specified that the DSMB consider stopping the trial for success with P(cumulative adjusted odds ratio [cOR]<1) greater than or equal to 95% and P(cOR<0.8) greater than or equal to 50% (statistical analysis plan in Supplement 3). The stopping rules for harm and safety were defined, respectively, as P(OR>1) greater than or equal to 80% and P(OR_adverse event_>1) greater than or equal to 75% (statistical analysis plan in Supplement 3). There were no prespecified stopping criteria for futility. However, after reviewing data on 920 participants on March 12, 2021, the DSMB recommended ceasing enrollment on March 15, 2021, based on slowing recruitment, the need for rapid reporting, and a 0.2% probability that the study would meet criteria for success if enrollment continued to 1000 participants.

### Statistical Analysis

The trial design used a bayesian approach based on continuous monitoring, allowing real-time decisions given the urgency to find effective treatment. There was no maximum sample size, but enrollment of 1000 participants was anticipated. We used a skeptical prior distribution, N(mean, 0; SD, 0.354) for the treatment effect to ensure a type I error rate less than 5% and conducted regular monitoring using bayesian techniques. Simulations based on prespecified criteria and found the type I error rate was less than 5%. Convergence of the bayesian models was confirmed through inspection of trace plots (eFigure 1 in Supplement 2).^29^

COVID-19 convalescent plasma and placebo recipient WHO scores were compared, with the placebo group as the reference arm. Primary and secondary outcomes were analyzed with a bayesian proportional cumulative odds model with adjustment for the following prespecified covariates: age, sex, prerandomization WHO score, symptom duration, and the stratification variables: risk status (high vs average) and study site. We examined goodness-of-fit of the model and confirmed the proportional odds assumption (eTable 1, eFigure 2 in Supplement 2).

For the primary outcome, CCP efficacy was defined as a cOR less than 1 and clinically meaningful effects were defined as cORs less than 0.8. Trial success was defined by posterior probability distributions of the cOR (P[cOR]): high, greater than or equal to 95% for effectiveness, and moderate, greater than or equal to 50% for clinical meaningfulness. Between-group differences were reported using point estimates based on median, 95% credible intervals (CrI), and posterior probabilities drawn from the estimated posterior distribution.

Analyses were performed using R, version 4.0.3 (R Foundation for Statistical Computing) (statistical analysis plan in Supplement 3).

## Results

### Participants

**Figure 1.**
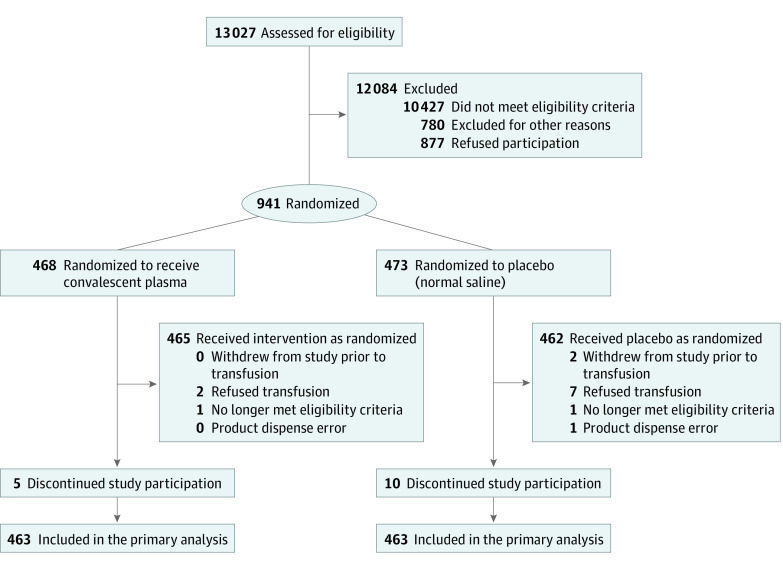
Patient Screening, Enrollment, and Treatment Assignment

From April 17, 2020, to March 15, 2021, 13 027 participants were evaluated; 941 were randomized (Figure 1). Day 28 follow-up of the last participant was completed on April 12, 2021. Of the 941 randomized participants, median age was 63 (IQR, 52-73) years, 556 patients were men (59.1%), 385 were women (40.9%), and 673 (71.5%) had prerandomization WHO scores of 5 (patient is hospitalized and requires oxygen by mask or nasal prongs). A total of 71 patients (7.5%) were Asian, 373 (39.6%) were Hispanic, 132 (14.0%) were non-Hispanic Black, and 318 (33.8%) were non-Hispanic White. Median time from symptom onset to randomization was 7 (IQR, 4-9) days; 468 patients were assigned to CCP and 465 (99.4%) received CCP; 473 patients were assigned to placebo and 462 (97.7%) received normal saline. A total of 924 participants (98.2%) completed the study and 17 patients (1.8%) withdrew (15 by day 14 and 2 by day 28). Primary analysis was done with 926 participants (463 CCP and 463 placebo recipients). Baseline characteristics were similar in the CCP and placebo groups (Table) and across participating sites (eTable 2 in Supplement 2).

**Table.** Demographic and Clinical Characteristics of the Patients at Randomization and Key Medications Initiated at or Prior to Randomization

Variable	No. with complete data	No. (%)			SMD
		Overall	Placebo	CCP	
No.		941	473	468	
Baseline characteristics					
Enrollment quarters	941				0.052
2020 Q2		170 (18.1)	86 (18.2)	84 (17.9)	
2020 Q3		113 (12.0)	53 (11.2)	60 (12.8)	
2020 Q4		407 (43.3)	208 (44.0)	199 (42.5)	
2021 Q5		251 (26.7)	126 (26.6)	125 (26.7)	
Age, median (IQR)	941	63.0 (52.0-73.0)	64.0 (54.0-74.0)	62.0 (51.0-72.0)	0.112
Age categorical, y	941				0.123
<45		126 (13.4)	59 (12.5)	67 (14.3)	
45-64		376 (40.0)	180 (38.1)	196 (41.9)	
65-80		321 (34.1)	168 (35.5)	153 (32.7)	
>80		118 (12.5)	66 (14.0)	52 (11.1)	
Sex	941				
Women		385 (40.9)	201 (42.5)	184 (39.3)	0.065
Men		556 (59.1)	272 (57.5)	284 (60.7)	0.065
Race and ethnicity^a^	941				0.124
Asian		71 (7.5)	30 (6.3)	41 (8.8)	
Hispanic		373 (39.6)	190 (40.2)	183 (39.1)	
Non-Hispanic Black		132 (14.0)	63 (13.3)	69 (14.7)	
Non-Hispanic White		318 (33.8)	165 (34.9)	153 (32.7)	
Other^b^		18 (1.9)	8 (1.7)	10 (2.1)	
Unknown		29 (3.1)	17 (3.6)	12 (2.6)	
BMI, median (IQR)^c^	940	30.4 (26.1-36.1)	29.7 (25.8-35.5)	31.0 (26.5-36.3)	0.044
WHO score of 5 at randomization	941	673 (71.5)	341 (72.1)	332 (70.9)	0.026
High risk^d^	941	777 (82.6)	388 (82.0)	389 (83.1)	0.029
Blood type	941				0.134
O		489 (52.0)	250 (52.9)	239 (51.1)	
A		274 (29.1)	138 (29.2)	136 (29.1)	
B		135 (14.3)	67 (14.2)	68 (14.5)	
AB		41 (4.4)	16 (3.4)	25 (5.3)	
Unknown		2 (0.2)	2 (0.4)	0 (0.0)	
Smoking history	941				0.033
Never		671 (71.3)	339 (71.7)	332 (70.9)	
Quit		227 (24.1)	114 (24.1)	113 (24.1)	
Yes		43 (4.6)	20 (4.2)	23 (4.9)	
Pregnancy	941	9 (1.0)	2 (0.4)	7 (1.5)	0.110
Time intervals, median (IQR), d					
Time between admission and randomization	941	1.0 (1.0-2.0)	1.0 (1.0-2.0)	1.0 (1.0-2.0)	0.067
Time between symptom onset and randomization	940	7.0 (4.0-9.0)	7.0 (4.0-9.0)	7.0 (4.0-9.0)	0.020
Time between symptom onset and randomization, d	940				0.081
<4		153 (16.3)	77 (16.3)	76 (16.3)	
4-7		436 (46.4)	217 (45.9)	219 (46.9)	
8-11		247 (26.3)	123 (26.0)	124 (26.6)	
12-15		70 (7.4)	40 (8.5)	30 (6.4)	
>15		34 (3.6)	16 (3.4)	18 (3.9)	
Comorbidities					
Pulmonary	941	97 (10.3)	47 (9.9)	50 (10.7)	0.025
Asthma	941	110 (11.7)	53 (11.2)	57 (12.2)	0.030
Hypertension	941	571 (60.7)	286 (60.5)	285 (60.9)	0.009
Cardiovascular	941	404 (42.9)	215 (45.5)	189 (40.4)	0.103
Diabetes	941	332 (35.3)	166 (35.1)	166 (35.5)	0.008
Chronic kidney disease	941	99 (10.5)	49 (10.4)	50 (10.7)	0.011
Liver disease	941	23 (2.4)	10 (2.1)	13 (2.8)	0.043
Cancer	941	106 (11.3)	52 (11.0)	54 (11.5)	0.017
Transplant	941	15 (1.6)	4 (0.8)	11 (2.4)	0.120
HIV and other immunodeficient states	941	12 (1.3)	6 (1.3)	6 (1.3)	0.001
Concomitant medications at randomization					
Hydroxychloroquine	941	33 (3.5)	17 (3.6)	16 (3.4)	0.010
Remdesivir	941	537 (57.1)	264 (55.8)	273 (58.3)	0.051
Corticosteroids					
Intravenous/oral^e^	941	721 (76.6)	365 (77.2)	356 (76.1)	0.026
Intranasal	941	99 (10.5)	48 (10.1)	51 (10.9)	0.024
Therapeutic anticoagulation^f^	941	736 (78.2)	368 (77.8)	368 (78.6)	0.020
Antiplatelets^g^	941	226 (24.0)	108 (22.8)	118 (25.2)	0.056
Anti-inflammatory agents^h^	941	267 (28.4)	136 (28.8)	131 (28.0)	0.017
Antipyretics^i^	941	546 (58.0)	283 (59.8)	263 (56.2)	0.074
Antibacterial agents	941	464 (49.3)	243 (51.4)	221 (47.2)	0.083
ACE inhibitors	941	63 (6.7)	35 (7.4)	28 (6.0)	0.057
Statins	941	265 (28.2)	128 (27.1)	137 (29.3)	0.049
Acid-reducing agents^j^	941	372 (39.5)	190 (40.2)	182 (38.9)	0.026
Laboratory results					
Baseline SARS-CoV-2 IgG, positive^k^	728	486 (66.8)	258 (68.8)	228 (64.4)	0.089
SARS-CoV-2 PCR test, positive	941	940 (99.9)	472 (99.8)	468 (100.0)	0.065
Neutrophil count, median (IQR), /μL	893	5700 (3700-8500)	5600 (4100-8500)	5800 (3300-8500)	0.05
Lymphocyte count, median (IQR), /μL	893	800 (500-1200)	800 (500-1100)	800 (500-1200)	0.045
Creatinine, median (IQR), mg/dL	939	0.8 (0.7-1.1)	0.8 (0.7-1.1)	0.8 (0.7-1.1)	0.019
D-dimer, median (IQR), ng/mL	895	594.0 (328.5-1165.0)	600.0 (334.0-1134.0)	584.0 (320.0-1204.0)	0.067
Fibrinogen, median (IQR), mg/dL	712	619.5 (526.8-700.0)	624.5 (527.5-700.0)	615.0 (525.8-700.0)	0.031
Lactate dehydrogenase, median (IQR), U/L	785	385.0 (301.0-513.0)	394.5 (299.3-511.3)	379.0 (305.0-514.5)	0.086
Ferritin, median (IQR), ng/mL	887	772.4 (392.5-1462.5)	753.9 (391.3-1437.8)	788.0 (412.0-1483.1)	0.004
C-reactive protein, median (IQR), mg/dL	891	7.8 (2.6-14.4)	8.1 (2.7-14.4)	7.5 (2.4-14.4)	0.032

Abbreviations: ACE, angiotensin-converting enzyme; BMI, body mass index; CCP, COVID-19 convalescent plasma; PCR, polymerase chain reaction;

SMD, standardized mean difference; WHO, World Health Organization.

SI conversion factors: To convert C-reactive protein to milligrams per liter, multiply by 10; creatinine to micromoles per liter, 88.4; D-dimer to nanomoles per liter, 5.476; ferritin to micrograms per liter, 1; fibrinogen to grams per liter, 0.01; lactate dehydrogenase to microkatals per liter, 0.0167; lymphocytes to ×10^9^ per liter, 0.001; and neutrophils to ×10^9^ per liter, 0.001.

^a^ Information on race and ethnic group was obtained from entries in the medical record, as reported by the patients.

^b^ Other included mixed race, American Indian or Alaska Native, and Native Hawaiian or other Pacific Islander.

^c^ BMI is calculated as weight in kilograms divided by height in meters squared.

^d^ Defined as participants aged 60 years or older or age younger than 60 years, and at least 1 of the high risk-comorbid conditions as per protocol.

^e^ Dexamethasone, prednisone, methylprednisolone, hydrocortisone.

^f^ Therapeutic dose of unfractionated heparin, low molecular weight heparin, warfarin, and direct-acting oral anticoagulants.

^g^ Aspirin, clopidogrel.

^h^ Interleukin (IL)-6 inhibitors, IL-1 inhibitors, tumor necrosis factor inhibitors, histamine antagonists, leukotriene inhibitors, mycophenolate mofetil, colchicine, intravenous immunoglobulin, CD20-inhibitors, phosphodiesterase 4-inhibitors, purine/pyrimidine synthesis inhibitors, interferon-β, aminosalicylate, and disease-modifying antirheumatic drugs.

^i^ Ibuprofen, acetaminophen, and other nonsteroidal anti-inflammatory drugs.

^j^ Proton pump inhibitors, H_2_ receptor blockers, and other antacids.

^k^ Defined as SARS-CoV-2 IgG titer greater than 1:100 using in-house full-length spike protein enzyme-linked immunosorbent assay.

### Primary and Secondary Outcomes

**Figure 2.**
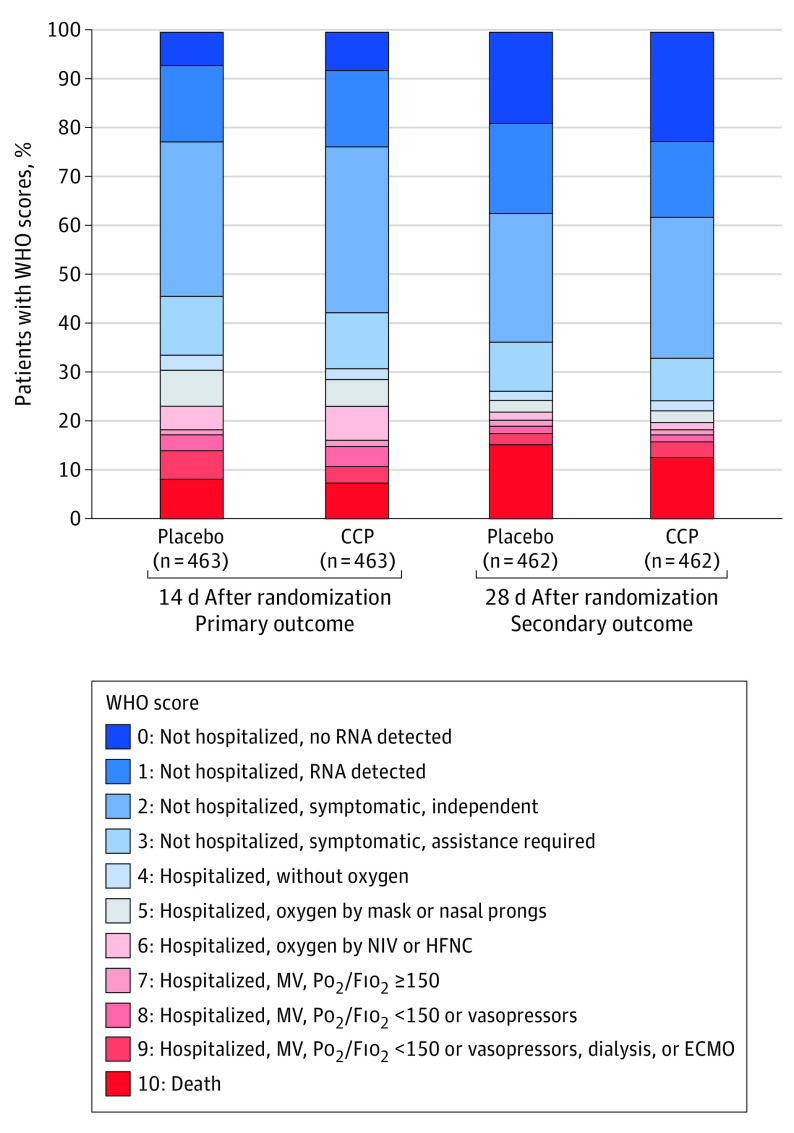
Primary and Secondary Outcomes by Treatment Group Distribution of clinical status assessed on the 11-point World Health Organization (WHO) Ordinal Scale for Clinical Improvement 14 and 28 days after randomization. ECMO indicates extracorporeal membrane oxygenation; HFNC, high-flow nasal cannula; MV, mechanical ventilation; NIV, noninvasive ventilation; PO_2_/FIO_2_, ratio of partial pressure of oxygen (PO_2_) to fraction of inspired oxygen (FIO_2_).

**Figure 3.**
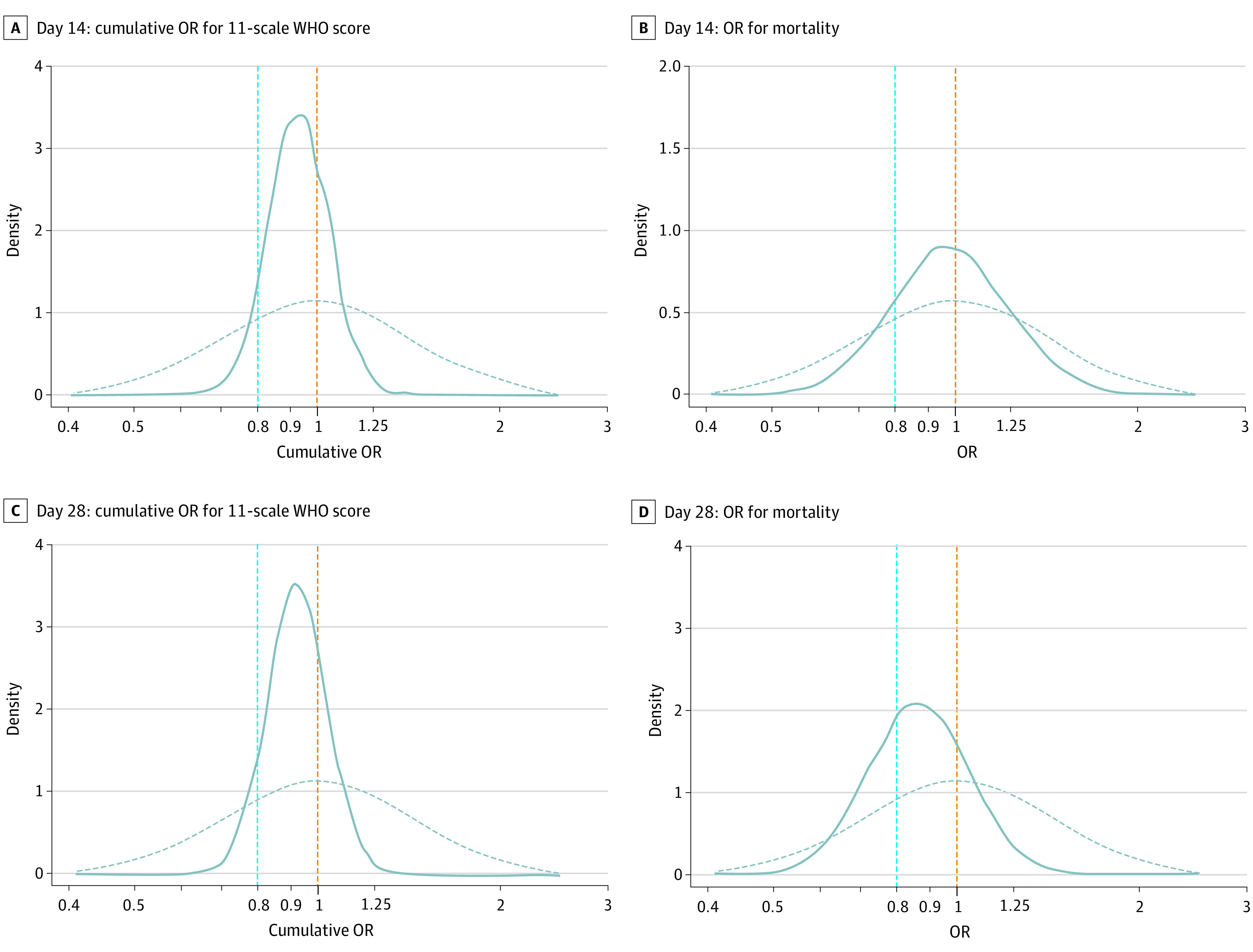
Posterior Distributions of Cumulative Odds Ratio (OR) for World Health Organization (WHO) Scores and OR for Mortality 14 and 28 Days After Randomization Posterior distribution of cumulative OR and OR estimates from bayesian models adjusted for sites, baseline risk, baseline WHO score, age, sex, and days since symptom onset to randomization (0-3, 4-7, or >7 days). Sites were combined within networks (New York University, Albert Einstein College of Medicine, Montefiore Medical Center, Yale University School of Medicine, University of Miami Miller School of Medicine, University of Texas Health Science Center at Houston, University of Texas Health Science Center at Tyler, Johns Hopkins University, and Medical College of Wisconsin Froedtert Hospital). The dashed curves represent the prior distribution assumptions for the ORs, and the solid curves represent the estimated posterior probability distributions of the ORs: P(ORs). The area under each solid curve totals 1, and the area to the left of the dashed orange line represents P(OR<1).

The primary (WHO scores on day 14) and secondary (WHO scores on day 28) outcomes, adjusted for prespecified covariates, did not meet prespecified definitions of efficacy (Figure 2 and Figure 3). At day 14, compared with placebo, CCP had an estimated median of the cOR of 0.94 (95% CrI, 0.75-1.18; P[cOR<1] = 72% and P[cOR<0.8] = 8%). At day 28, the cOR was 0.92 (95% CrI, 0.74-1.16; P[cOR<1] = 76% and P[cOR<0.8] = 10%).

### Tertiary Outcome

At day 14, 35 of 463 (7.6%) CCP recipients and 39 of 463 (8.4%) placebo recipients had died. At day 28, 59 of 462 (12.8%) CCP recipients and 71 of 462 (15.4%) placebo recipients had died (Figure 2). The day 14 median OR (0.99; 95% CrI, 0.64-1.53; P[OR<1] = 53% and P[OR<0.8] = 17%), and day 28 OR (0.86; 95% CrI, 0.60-1.25; P[OR<1] = 78% and P[OR<0.8] = 34%) did not meet prespecified thresholds for efficacy (Figure 3).

### Exploratory and Post Hoc Subgroup Analyses

As the trial neared completion, it was apparent there were differences in participant characteristics over time. Between April-June and July-September 2020, median participant age decreased (from 70 to 59 years), while increases were noted in symptom duration less than 7 days (from 43.5% to 73.5%), high-risk status (from 62% to 90%), remdesivir use (from 1% to 47%), and corticosteroid use (from 24% to 85%) (eTable 3 in Supplement 2). Thus, we conducted a post hoc analysis to assess heterogeneous treatment effects across time, analyzing the data by enrollment quarter (Q): Q2, April-June 2020; Q3, July-September 2020; Q4, October-December 2020; and Q5, January-March 2021. At day 28, cORs comparing WHO scores of CCP participants with placebo participants were 0.72 (95% CrI, 0.46-1.13; P[cOR<1] = 93%) in Q2, 0.83 (95% CrI, 0.50-1.39; P[cOR<1] = 77%) in Q3, 0.99 (95% CrI, 0.72-1.37; P[cOR<1] = 52%) in Q4, and 1.18 (95% CrI, 0.81-1.74; P[cOR<1] = 19%) in Q5 (eTables 4-7, eFigures 3-5 in Supplement 2). The probability of death for all participants was highest in Q2 when all enrollments were at NYC and Long Island, New York, sites (eFigure 6 and eFigure 7 in Supplement 2).

**Figure 4.**
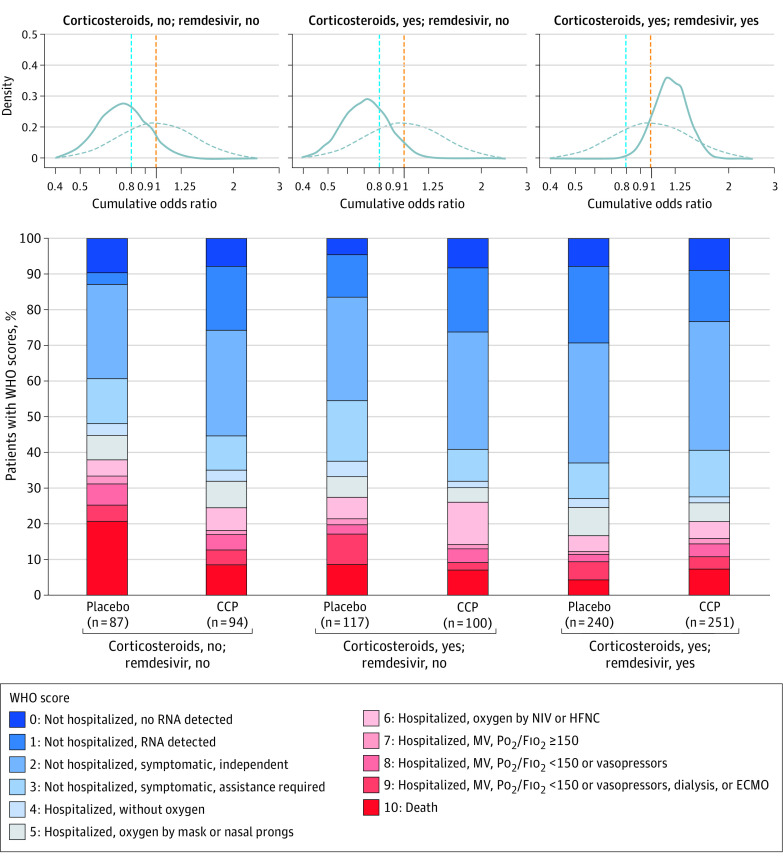
Clinical Outcomes among Patients Treated With COVID-19 Convalescent Plasma and Placebo 14 Days After Randomization by Remdesivir/Corticosteroid Use Distribution of clinical status assessed on the 11-point WHO Ordinal Scale for Clinical Improvement 14 days after randomization by remdesivir and/or corticosteroid use shown by cumulative OR (curves) and WHO scores (stacked bars). In the top panel, the dashed curves represent the prior distribution assumptions for the ORs, and the solid curves represent the estimated posterior probability distributions of the ORs: P(ORs). The area under each solid curve totals 1, and the area to the left of the dashed orange line represents P(OR<1). ECMO, extracorporeal membrane oxygenation; HFNC, high-flow nasal cannula; MV, mechanical ventilation; NIV, noninvasive ventilation; PO_2_/FIO_2_, ratio of partial pressure of oxygen (PO_2_) to fraction of inspired oxygen (FIO_2_).

We assessed heterogeneity in treatment effects based on remdesivir and/or corticosteroid use at randomization (eTable 8 and eTable 9 in Supplement 2). At day 14, the cOR for participants not receiving remdesivir or corticosteroids (with most enrolled in Q2) was 0.74 (95% CrI, 0.48-1.15; P[cOR<1] = 92%) and, for those receiving corticosteroids but not remdesivir, 0.71 (95% CrI, 0.47-1.06; P[cOR<1] = 95%), which were lower than the cORs in patients receiving both medications: 1.19 (95% CrI, 0.89-1.60; P[cOR<1] = 12%) (Figure 4; eTable 4, eTable 5, and eFigure 4 in Supplement 2). At day 28, the cORs of participants not receiving either medication, 0.65 (95% CrI, 0.41-1.02; P[cOR<1] = 97%) and those receiving corticosteroids but not remdesivir, 0.84 (95% CrI, 0.56-1.27; P[cOR<1] = 79%) were lower than those receiving both agents, 1.14 (95% CrI, 0.85-1.54; P[cOR<1] = 19%) (eTable 6, eTable 7, and eFigure 5 in Supplement 2). The posterior probabilities of death at days 14 and 28 were lower in participants receiving corticosteroids and remdesivir at randomization, irrespective of treatment arm, without adjustment for covariates (eFigure 6 and eFigure 7 in Supplement 2). At day 14, the cOR was 0.94 (95% CrI, 0.62-1.45; P[OR<1]=61%) in those who did not receive anticoagulation and 0.93 (95% CrI, 0.73-1.19; P[OR<1]=71%) in those who received anticoagulation.

The effects of CCP also differed by participant age, WHO score, and symptom duration at randomization. At day 28, cORs were lower for participants aged 65 years or older (0.84; 95% CrI, 0.62-1.14; P[cOR<1] = 87%) than those younger than 65 years (1.03; 95% CrI, 0.76-1.38; P[cOR<1] = 43%) and for those with WHO scores of 5 (0.89; 95% CrI, 0.69-1.15; P[cOR<1] = 82%) than 6 (1.00; 95% CrI, 0.68-1.47; P[cOR<1] = 50%). Posterior probabilities based on symptom duration exhibited considerable uncertainty (eTables 4-7, eFigure 4 and eFigure 5 in Supplement 2), and for death, increased with shorter symptom duration (eFigure 6 and eFigure 7 in Supplement 2).

### CCP SARS-CoV-2 Spike Protein IgG and Neutralizing Titers

The overall median CCP EC_50_, 1:2016 (IQR, 916-4229), was highest in Q5 (1:3596 [IQR, 2179-6097]), then Q2 (1:2047 [IQR, 677-5400]). The median CCP neutralizing titer (1:93 [IQR, 48-213], 69% < 1:160) was highest in Q2 (1:175 [IQR, 76-379]) then in Q5 (1:106 [IQR, 63-235]) (eTable 10 in Supplement 2). Mortality appeared to be lower for CCP recipients who received high EC_50_ CCP than placebo in Q2 (eFigure 8 in Supplement 2), but there were no significant associations between CCP EC_50_ or neutralizing titer and clinical outcome after adjustment for covariates.

### Pretreatment Participant Plasma SARS-CoV-2 Spike Protein IgG

Plasma SARS-CoV-2 IgG was present before randomization in 486 (66.8%) of 728 participants from whom samples were available. At day 28, mortality (WHO score of 10) was lower in 486-seropositive than 242-seronegative participants irrespective of treatment arm, and in seronegative CCP (14.4%) than placebo (17.9%) recipients, which did not meet the definition of efficacy (eTable 11, eFigure 7 in Supplement 2), but analysis was restricted by sample availability, particularly for Q2 (62 samples available, 170 randomized).

### Safety Outcomes and Adverse Events

There were no episodes of transfusion-related acute lung injury or transfusion-associated circulatory overload reported. Any adverse events (excluding transfusion reactions) were reported for 39 (8.2%) of placebo participants and 44 (9.4%) of CCP recipients (*P* = .57). There were 2 (0.4%) transfusion reactions in placebo recipients and 8 (1.7%) in CCP recipients (*P* = .06) (eTable 12 in Supplement 2).

## Discussion

The CONTAIN COVID-19 trial was initiated in April 2020 during the first pandemic wave in NYC and Long Island, expanded to other US sites in August 2020, and continued until March 2021, spanning 11 months during which COVID-19 care changed substantially. The primary outcome did not meet the prespecified definition for CCP efficacy. However, exploratory subgroup analyses revealed a possible benefit of CCP in Q2 (April-June 2020), when all participants were enrolled in NYC and Long Island, most received high-titer CCP, and most did not receive remdesivir and/or corticosteroids. These medications were incorporated into COVID-19 care after the corticosteroid results from the RECOVERY trial were reported in July 2020^30^ and the US Food and Drug Administration issued an emergency use authorization for remdesivir in May 2020 followed by approval in October 2020.^31,32^

Consistent with the ACTT-I^32^ and RECOVERY^30^ trial results, remdesivir and corticosteroids appeared to improve clinical status irrespective of treatment arm. However, in the CONTAIN COVID-19 trial, use of these medications at randomization resulted in heterogeneous treatment effects. At day 14, CCP use appeared to improve clinical status when only corticosteroids were in use, but there was no evidence of CCP benefit when remdesivir and corticosteroids were both in use, and those who received both may have done worse. Our trial cannot establish the effect of these medications on CCP efficacy; they were not randomized, the trial was not designed to investigate their effects, and the analyses were exploratory. Nonetheless, based on other trial results, interactions between CCP, corticosteroids, and remdesivir warrant further investigation.^13,17^ A randomized clinical trial in which 81% of 223 participants received corticosteroids and 6% received remdesivir found a CCP mortality benefit.^13^ However, the 11 558-participant RECOVERY trial, in which 93% of 5795 recipients of CCP received corticosteroids and 32% received remdesivir, did not find a CCP mortality benefit, although CCP recipients not receiving corticosteroids appeared less likely to be intubated or die than controls (18% vs 24%; *P* = .07).^17^ Data for remdesivir were not reported. Further studies are needed to understand interactions between CCP, corticosteroids, and remdesivir.

We found no associations between clinical outcome and CCP EC_50_ or neutralizing titer, or participant SARS-CoV-2 serostatus. Less than 15% of our cohort had cancer or other immunosuppressing conditions that are associated with an impaired SARS-CoV-2 antibody response. A benefit of CCP has been shown in these patients.^33^ The largest CCP effect was in Q2, particularly at day 28, when its effect (P[cOR<1] = 93%) approached the prespecified bayesian definition of efficacy. Retrospective analysis showed the median Q2 CCP-neutralizing titer was greater than 1:160, which likely fulfilled criteria for high-titer CCP,^34,35^ whereas the CCP that was used during Q3 to Q5 was likely not high titer. Recently aggregated randomized clinical trial data suggest high-titer CCP is necessary, although it may not be sufficient, to benefit hospitalized patients with COVID-19.^36^ Clearly, there is a need for standardized platforms and thresholds to qualify CCP for use. Nonetheless, CCP may have had heterogeneous effects over time as viral variants changed in this population. The effect of SARS-CoV-2 variants on our results is unknown, but 60% of Q5 enrollments were at NYC sites when the alpha and iota variants predominated,^37^ and surveillance data identified alpha and beta variants in Miami and alpha in Houston.^38^

Although exploratory subgroup analyses suggested CCP may be beneficial in participants aged 65 years or older and those with less severe disease (WHO 5), the posterior probabilities of these findings exhibited considerable uncertainty. Nonetheless, consistent with these findings, other hospitalized patient studies identified a possible CCP benefit in older patients^13,14,15,17^ and those with less severe disease.^9,14,17^ Given the absence of overall CCP benefit in our trial and randomized clinical trials of hospitalized patients with severe to life-threatening disease,^14,15,16,17,18,36^ it is possible that patients with less severe disease could benefit the most from CCP therapy. Further insight may come from the COMPILE cohort, which included patients not requiring oxygen (WHO 4).^39,40^

### Strengths and Limitations

Strengths of the trial include its multicenter, blinded nature and use of a placebo control; an 11-month enrollment period that provided insights into CCP efficacy as COVID-19 treatments were being developed; a highly diverse population that allows for generalizability; and use of a bayesian statistical approach that allowed near real-time monitoring of accruing data.

Limitations of the trial include that the primary outcome at day 14 was likely too early for a disease now known to have a prolonged course. Therefore, day 28 findings may be more important clinically. In addition, there were heterogeneous treatment effects over time, perhaps related to changing patient characteristics, treatment options, and other factors. Compared with Q3 to Q5, Q2 participants were older, most received CCP with a median neutralizing titer greater than 1:160 and were not receiving remdesivir or corticosteroids. COVID-19 convalescent plasma obtained in the NYC area was used in non-NY sites and may not have matched local viral species,^38,41^ and emergence of SARS-CoV-2 variants, which were not studied, may have reduced CCP efficacy over time. Because most Q3 to Q5 participants received CCP with a neutralizing titer less than 1:160, more than 1 unit may have been beneficial.^18^ Participants with shorter symptom duration had higher mortality and we may have inadvertently enrolled patients with more severe disease by using symptom duration as an inclusion criterion. Analysis of the association between serostatus and CCP efficacy, as done by others^42^ was restricted by sample availability.

## Conclusions

This placebo-controlled double-blind randomized clinical trial of use of CCP in hospitalized patients with COVID-19 requiring noninvasive oxygen supplementation did not meet the prespecified definition of CCP efficacy. However, a possible benefit of CCP was observed early in the pandemic when high-titer CCP was used and corticosteroids and remdesivir were not in use. This supports the concept that convalescent plasma may be a feasible treatment option at the beginning of a pandemic or when other therapies are not in use or available. Further investigation is needed to understand the effects of corticosteroids and remdesivir on CCP efficacy and establish thresholds for antibody quantity and function that are most likely to confer a benefit.

## Notes

Supplement 1. Trial Protocol

Supplement 2. eMethods. Expanded Methods **eTable 1.** Summary of Posterior Predictive Checks for 10 Test Statistics Based on 10 000 Replicated Data sets **eTable 2.** Baseline Characteristics by Enrollment Sites and Treatment Group **eTable 3.** Baseline Patient Characteristics by Enrollment Quarter **eTable 4.** Cumulative Odds Model of WHO Scores at Day 14 by Subgroups **eTable 5.** Odds Ratios for Mortality at Day 14 by Subgroups **eTable 6.** Cumulative Odds Model of WHO Scores at Day 28 by Subgroups **eTable 7.** Odds Ratio for Mortality at Day 28 by Subgroups **eTable 8.** Baseline Patient Characteristics by Remdesivir Use at Randomization **eTable 9.** Baseline Patient Characteristics by Corticosteroids Use at Randomization **eTable 10.** CCP SARS-CoV-2 IgG and Neutralizing Titers by Quarters of Enrollment **eTable 11.** Baseline Characteristics and Day 14/28 Outcomes by Baseline SARS-CoV-2 IgG Status and Treatment Group **eTable 12.** Adverse Events and Serious Adverse Events **eFigure 1.** Trace Plots of Model Convergence **eFigure 2.** Observed Cumulative Probability With Predicted 95% Credible Interval by Treatment Group **eFigure 3.** Clinical Outcomes Among Patients Treated With Convalescent Plasma and Placebo 14 and 28 Days After Randomization by Enrollment Quarter **eFigure 4.** Cumulative OR for WHO Ordinal Scale and OR for Mortality at Day 14 in Indicated Subgroups **eFigure 5.** Cumulative OR for WHO Ordinal Scale and OR for Mortality at Day 14 in Indicated Subgroups **eFigure 6.** Posterior Probability of Mortality at Day 14 of Placebo and CCP Recipients in Indicated Subgroups Without Adjustment for any Covariates **eFigure 7.** Posterior Probability of Mortality at Day 28 of Placebo and CCP Recipients in Indicated Subgroups Without Adjustment for any Covariates **eFigure 8.** Clinical Outcome in Placebo and CCP Groups Dichotomized by Median CCP SARS-CoV-2 IgG EC_50_ at 14 and 28 Days After Randomization
eReferences

Supplement 3. Statistical Analysis Plan

Supplement 4. Nonauthor Collaborators. The CONTAIN COVID-19 Study Group

Supplement 5. Data Sharing Statement
