# Supplementary material for: Efficacy and Safety of COVID-19 Convalescent Plasma in Hospitalized Patients: A Randomized Clinical Trial
Source: JAMA Intern Med. 2021 Dec 13;182(2):1–12. doi: 10.1001/jamainternmed.2021.6850 (PMC8669605; doi:10.1001/jamainternmed.2021.6850)
Supplement: Supplement 1. — Trial Protocol [file jamainternmed-e216850-s001.pdf]

Efficacy and Safety of COVID-19 Convalescent Plasma in Hospitalized Patients, A Randomized Clinical Trial

This supplement contains the following items:

1. Original Protocol (Page 2)

2. Final Protocol (Page 28)

3. Summary of Changes in the Protocol (Page 57)

CONVALESCENT PLASMA TO LIMIT CORONAVIRUS ASSOCIATED COMPLICATIONS: A RANDOMIZED BLINDED PHASE 2 STUDY COMPARING THE EFFICACY AND SAFETY OF ANTI-SARS-COV-2 PLASMA TO PLACEBO IN COVID-19 HOSPITALIZED PATIENTS

Protocol Version: 1.0
Protocol Date: April 13, 2020

LIST OF ABBREVIATIONS

ADR: Adverse Drug Reaction
ADE: Antibody-mediated enhancement of infection
AE: Adverse Event/Adverse Experience
CBC: Complete Blood Count
CDC: United States Centers for Disease Control and Prevention
CFR: Code of Federal Regulations
CLIA: Clinical Laboratory Improvement Amendment of 1988
COI: Conflict of Interest
COVID-19: Coronavirus Disease
CRF: Case Report Form
CRP: C-Reactive Protein
DMC: Data Management Center
DSMB: Data and Safety Monitoring Board
EUA: Emergency Use Authorization
FDA: Food and Drug Administration
GCP: Good Clinical Practice
HBV: Hepatitis B virus
HCV: Hepatitis C virus
HFNC: High flow nasal cannula
HIV: Human immunodeficiency virus
HTLV: Human T-cell lymphotropic virus
IB: Investigator's Brochure
ICF: Informed Consent (Informed Consent Form)
ICH: International Conference on Harmonization
ICU: Intensive Care Unit
IEC: Independent Ethics Committee
IND: Investigational New Drug Application
IRB: Institutional Review Board
ISBT: International Society of Blood Transfusion
ISM: Independent Safety Monitor
IWRS: Interactive Web Response System
LOS: Length of Stay
MERS: Middle East Respiratory Syndrome
NEWS 2- National Early Warning Score
NP: Nasopharyngeal
OHRP: Office of Human Research Protections
OP: Oropharyngeal
RT-PCR: Reverse Transcriptase Real-Time Polymerase Chain Reaction
PK: Pharmacokinetic
SAE: Serious Adverse Event
SARS: Severe Acute Respiratory Syndrome
SARS-CoV-2: Severe Acute Respiratory
TACO: Transfusion-associated Circulatory Overload
T. cruzi: *Trypanosoma cruzi*
TRALI: Transfusion-related Acute Lung Injury
UP: Unanticipated Problem
UPnonAE: Unanticipated Problem that is not an Adverse Event

PROTOCOL SUMMARY

Long title: Convalescent Plasma to Limit Coronavirus Associated Complications: A Randomized Blinded Phase 2 Study Comparing the Efficacy and Safety of Anti-SARS-CoV-2 Plasma to Placebo in COVID-19 hospitalized patients

Clinical Phase: 2 Blinded

Sample Size: 300

Study Population: Hospitalized COVID-19 patients aged ≥18 years of age with respiratory symptoms within 3 to 7 days from the onset of illness OR within 3 days of hospitalization.

Study Duration: April 20, 2020 to January 31, 2023

Study Design: This randomized, blinded phase 2 trial will assess the efficacy and safety of anti-SARS-CoV-2 convalescent plasma in hospitalized patients with acute respiratory symptoms between 3 and 7 days after the onset of symptoms OR within 3 days of hospitalization. A total of 300 eligible subjects will be randomized in a 1:1 ratio to receive either convalescent plasma from people who have recovered from Covid-2 containing antibodies to SARS-CoV-2 or control (standard thawed plasma).
Stratified randomization by site and risk (high versus average risk).

Underlying risk based on pre-COVID 19 infection baseline characteristics:

- <u>High risk</u>: Any of the following: ≥60 years or age, immune compromised (immunosuppressive drugs, solid tumor, solid organ transplant, hematologic malignancies, hematologic stem cell transplantation, rheumatological disease on immunosuppressants, inflammatory bowel disease on immunosuppressants, asthma or COPD on chronic steroid therapy), diabetes mellitus, cardiovascular or pulmonary co-morbidities, HIV (CD4<200)

- <u>Average risk</u>: <60 years of age AND absence of immune compromise, diabetes mellitus, cardiovascular, pulmonary comorbidities, HIV (CD4>200)

Data Collection:

The following will be assessed in all subjects: (frequency noted in Table 1)

<u>I. Clinical, Laboratory and Imaging Data</u>

1. Date of Symptom Onset and history of presenting illness
2. Demographics: Age, sex, comorbidities, zip code, race/ethnicity, BMI
3. Vital Signs: Temperature, respiratory rate, blood pressure, oxygen saturation, oxygen requirements, NEWS score
4. Laboratory Data:
   - Hematologic Markers: CBC with differential (neutrophil, lymphocyte counts and platelet count explicitly recorded), PTT, LDH, D-dimer, fibrinogen, ferritin, CD4-CD8 counts
   - Metabolic Markers: Creatinine, arterial blood gas, LFTs
   - Cardiac Markers: Troponin
   - Inflammatory Markers: CRP, procalcitonin, IL6, cytokine panel
5. Chest imaging (CT or Chest x-ray), EKG and/or ultrasound: Day 0, and day14 or discharge whichever comes first and times obtained as part of standard care

<u>II. Safety and Efficacy</u>

1. Day 0 (baseline), 1, 2, 3, 7, 14, and 28 and once at 2-3 months.

III. SARS-CoV-2 Viral and Antibody Response – to be done at Montefiore Medical Center Sites

1. Serum or plasma antibody titer[1] to SARS-CoV-2: Day 0, 1, 7, 14, 28, 90
2. SARS-CoV-2 PCR from nasopharyngeal swab, quantitative if available: Day 0, 7, 14, 28

IV. Outcome measures:

Primary Outcome: Status at 14 days (1 through 5). Effect size will be measured as the cumulative odds ratio comparing treatment to control, estimated using a cumulative proportional odds model that adjusts for initial status (indicator for status = 3 or status = 4).

Study Agent:

- SARS-CoV-2 convalescent plasma (1-2 units; ~250-500 mL with antibodies to SARS-CoV-2[1] per April 8, 2020 directive https://www.fda.gov/vaccines-blood-biologics/investigational-new-drug-ind-or-device-exemption-ide-process-cber/recommendations-investigational-covid-19-convalescent-plasma, pathogen reduced) obtained from New York Blood Center or American Red Cross
- Standard plasma[2]

Primary Objective: Evaluate the efficacy of convalescent plasma from people who have recovered from Covid-19 containing antibodies to SARS-CoV-2 versus control (standard plasma) to prevent worsening respiratory status or death in hospitalized patients with COVID-19 who are within 3 days of presentation to the hospital or 3-7 days of symptom onset.

Primary Endpoint:
Primary Outcome: Status at 14 days (1 through 5, defined below). Effect size will be measured as the cumulative odds ratio comparing treatment to control, estimated using a cumulative proportional odds model that adjusts for initial status (indicator for status = 3 or status = 4).

Clinical Status Scale
1. Death;
2. Hospitalized, on invasive mechanical ventilation or extracorporeal membrane oxygenation (ECMO);
3. Hospitalized, on non-invasive ventilation or high flow oxygen devices;
4. Hospitalized, requiring supplemental oxygen;
5. Discharged alive

Score 3 or 4 describes eligibility and a score of 1-5 defines the outcome status at 14 days:

Secondary Objectives:
Secondary outcome; same as above at 28 days.

Exploratory objectives:
1. Serum or plasma anti-SARS-CoV-2 IgM, IgG, IgA on Days 0, 7, 14, 28, 90
2. Serum or plasma SARS-CoV-2 neutralizing activity and antibody dependent cytotoxicity (ADCC) on Days Day 0, 7, 14, 28, 90 (To be primarily done at Albert Einstein)

---

[1] Measured via initial anti-SARS-CoV-2 ELISA.

[2] Solvent detergent treated pooled plasma

3. Rates, levels and duration of SARS-CoV-2 RNA in NP swabs using RT-PCR on days 0, 7, 14, 28. Other specimen types may be tested as available (*e.g.*, BAL fluid, tracheal secretions, sputum, etc.).
4. Lymphocyte and neutrophil counts on days 0, 3, 7, 14 or as obtained in care.
5. Hematological measurements (D-dimer, fibrinogen) on days 0, 3, 7, 14 or as obtained in care.
6. T and B cell subsets on days 0, 7, 28 (To be done primarily at Albert Einstein)

Safety

1. Cumulative incidence of adverse events during the study period: transfusion reaction (fever, rash), transfusion related acute lung injury (TRALI), transfusion associated circulatory overload (TACO), transfusion related infection.

Study population

Inclusion Criteria for Enrollment:

1. Patients ≥18 years of age
2. Hospitalized for COVID-19 respiratory symptoms
3. Hospitalized for less than 72 hours OR within day 3 to 7 from first signs of illness
4. Laboratory confirmed COVID-19
5. On supplemental oxygen, non-invasive ventilation or high-flow oxygen (clinical status 3 or 4)
6. N.B. It is assumed patients may be on other randomized controlled trials of pharmaceuticals for COVID -19 and patients who meet eligibility criteria will not be excluded on this basis.

Exclusion Criteria

1. Receipt of pooled immunoglobulin in past 30 days
2. Contraindication to transfusion or history of prior reactions to transfusion blood products
3. Invasive mechanical ventilation or extracorporeal membrane oxygenation (ECMO)
4. Volume overload secondary to congestive heart failure or renal failure

RATIONALE/BACKGROUND:

1. **<u>Background and scientific rationale</u>**

There are currently no proven treatment options for coronavirus disease (COVID-19) and the related pneumonia, caused by Severe Acute Respiratory Syndrome Coronavirus 2 (SARS-CoV-2) beyond supportive care. Human convalescent plasma is a treatment option for COVID-19 and could be rapidly available when there are enough people who have recovered and donate high titer (anti- SARS-CoV-2) neutralizing immunoglobulin-containing plasma. As more individuals contract COVID-19 and recover, the number of potential donors will continue to increase, to allow greater use.

Use of convalescent plasma is a form of passive antibody therapy that involves the administration of antibodies to a given agent to a susceptible individual for the purpose of preventing or treating the infectious disease it causes. In contrast, active vaccination requires the induction of an immune response that takes time to develop and varies in efficacy depending on the vaccine recipient. Some immunocompromised patients fail to achieve an adequate immune response to active vaccination and at present, there are no vaccines to prevent COVID-19. When given to a susceptible person, antibody used for therapy will circulate in the blood, reach tissues and

hopefully mediate a beneficial effect by anti-microbial anti-inflammatory activity (1). Depending on antibody amount and composition, protection conferred by transferred immunoglobulin can last from weeks to months.

Passive antibody administration is the only means of providing immediate immunity to susceptible persons and immunity of any measurable kind for susceptible, including immunocompromised patients with Covid-19. This kind of antibody therapy has a storied history going back to the 1890s and was the only means of treating certain infectious diseases prior to the development of antimicrobial therapy in the 1940s (2, 3). Experience from prior outbreaks with other coronaviruses, such as SARS-CoV-1 shows that such convalescent plasma contains neutralizing antibodies to the relevant virus (4). In the case of SARS-CoV-2, the anticipated mechanism of action by which passive antibody therapy would mediate protection is viral neutralization. However, other mechanisms may be possible, such as antibody dependent cellular cytotoxicity and/or phagocytosis. Convalescent serum was also used in the 2013 African Ebola epidemic. A small non-randomized study in Sierra Leone revealed a significant increase in survival for those treated with convalescent whole blood relative to those who received standard treatment (5).

When used for therapy, antibody is most effective when administered shortly after the onset of symptoms. The reason for temporal variation in efficacy is not well understood but could reflect that passive antibody works by neutralizing the initial inoculum, which is likely to be much smaller than that of established disease. Another explanation is that antibody works by modifying the inflammatory response, which is also easier during the initial immune response, which may be asymptomatic (6). For example, passive antibody therapy for pneumococcal pneumonia was most effective when administered shortly after the onset of symptoms and there was no benefit if antibody administration was delayed past the third day of disease (2). Clinical outcomes after convalescent antibody therapy were better when it was administered to ill patients SARS-CoV-1 within 14 days after onset of symptoms (discussed below) (7). Our goal is to treat patients who are sick enough to warrant hospitalization but do not have severe respiratory disease and/or ARDS.

2. Experience with the use of convalescent plasma against coronavirus diseases

In the 21st century, there were two other epidemics with coronaviruses that were associated with high mortality, SARS1 in 2003 and MERS in 2012. In both outbreaks, the high mortality and absence of effective therapies led to the use of convalescent plasma. The largest study involved the treatment of 80 patients in Hong Kong with SARS (7). Consistent with historical data that earlier administration of antibody is more likely to be effective, 30 patients treated a mean of 11.7 (+/- 2.3) days after symptom onset had improved prognosis defined by discharge from hospital before day 22, whereas 47 patients who received plasma a mean of 16 days after symptom onset died before day 22 or had a late discharge. The mortality rates in the two groups were 6.3% and 21.9%, respectively (P=0.08), and those who were nasal swab PCR positive and seronegative for coronavirus at the time of therapy had improved prognosis. There is also some anecdotal information on the use of convalescent plasma in seriously ill individuals. Three patients with SARS in Taiwan were treated with 500 ml of convalescent plasma, resulting in a reduction in plasma virus titer and each survived (8). Three patients with MERS in South Korea were treated with convalescent plasma, but only two of the recipients had neutralizing antibody in their plasma (9). The latter study highlights a challenge in using convalescent plasma; some who recover may not have high titers of neutralizing antibody (10). An analysis of 99 samples of convalescent sera from patients with MERS showed 87 had neutralizing antibody with a geometric mean titer of 1:64. This suggests that antibody declines with time and/or only a few patients make high titer responses. Our study addresses this issue by screening plasma for antibody titers to SARS-CoV-2 and using high titer antibody for treatment. Although the optimal titer for treatment of SARS-CoV-2 is not established, plasma with a neutralizing titer of at least 1:64 should be administered. However, it is possible non-neutralizing antibodies may also contribute to protection as described for other viral diseases (11, 12).

A recently performed pilot study in Wuhan, China collected convalescent plasma from COVID-19 positive patients 3 weeks following the onset of illness and 4 days post-discharge and treated patients diagnosed with 'severe COVID-19' as defined by WHO Interim Guidance and the Guideline of Diagnosis and Treatment of COVID-19 National Health Commission of China (13). Ten patients were treated with one dose of convalescent plasma (200ml, >1:640 titer by neutralization assay) at a median of 16.5 days (11-19.3 days) post-onset of symptoms. A COVID-19 positive control cohort was retrospectively identified and matched by demographics, comorbidities, and severity of illness. There were no serious adverse reactions or safety events recorded with convalescent plasma, including no reported transfusion related reactions, transfusion-related acute lung injury, or antibody-mediated enhancement of infection. In the treatment group, there were 0 deaths, 3 discharges and 7 patients improved, whereas there were 3 deaths and 7 patients who improved in the control group ($p < 0.001$). In addition, 2 of 3 patients in the treatment on mechanical ventilation were weaned to high flow nasal canula, which as discontinued in one patient. There was a reduction in blood RNA viral load in 7 of 10 patients on day 6 post-convalescent plasma therapy as well as improvement in laboratory markers. There were also varying degrees of improvement in pulmonary lesions on chest CT after convalescent plasma therapy. In another case series from China, five severely ill patients with COVID-19, all on mechanical ventilation received convalescent plasma within 22 days of admission (14). Temperatures normalized in 4 of 5 patients within 3 days, NEWS scores decreased, and there was improvement in oxygenation and ARDS resolution. All survived, with 3 discharged home and 2 in stable condition. These reports suggest convalescent plasma may hold promise for ameliorating the severity of Covid-19 and deserves immediate investigation for this indication.

There are limited data on use of convalescent plasma in pregnancy. A non-randomized comparative study that evaluated use of convalescent plasma for Ebola Virus Disease reported that eight out of 84 participants were pregnant and that mortality was 25% among pregnant women and 32% among non-pregnant individuals after receiving plasma treatment (van Griensven et al. N Engl J Med 2016). A case series of 4 Chinese patients, among whom one was pregnant, received convalescent plasma and had recovered from SARS-CoV-2 infection (Zhang B, et al. Chest 2020 Mar 31). We do not have robust data of using convalescent plasma in pregnancy as most trials have excluded pregnant patients. IVIG therapy however is safe to give during pregnancy and is often used in those with inflammatory disorder and/or autoimmune conditions.

INVESTIGATIONAL PLAN:

1. Study Objectives

Primary Objective:

1. Evaluate the efficacy of high-titer anti-SARS-CoV-2 plasma versus control (standard plasma) to prevent worsening respiratory failure or death in hospitalized patients with COVID-19 who are within 3 days of presentation to the hospital or 3-7 days of symptom onset.

Secondary Objectives:
Secondary outcome; same as above at 28 days.

Exploratory objectives:

1. Compare the study (anti-SARS-CoV-2 convalescent plasma) and control (standard plasma) groups anti-SARS-CoV-2 titers at Days 0, 7, 14, 28, 90.
2. Compare the rates, levels and duration of SARS-CoV-2 RNA in NP swabs using RT-PCR between the study (anti-SARS-CoV-2 convalescent plasma) and control (standard plasma) groups at days 0, 7 14, 28. Other specimen types may be tested as available (*e.g.*, BAL fluid, tracheal secretions, sputum, etc.) or when RT-PCR assays are validated for additional sources (*i.e.*, stool, blood).
3. Mortality, in-hospital and Day 28; rates of ICU admission

4. Lymphocyte and neutrophil counts on days 0, 3, 7, 14 or as obtained in care; Hematological measurements (D-dimer, fibrinogen) on days 0, 3, 7, 14 or as obtained in care; and T and B cell subsets on days 0, 7, 28.

Safety

Cumulative incidence of serious adverse events during the study period: transfusion reaction (fever, rash), transfusion related acute lung injury (TRALI), transfusion associated circulatory overload (TACO), transfusion related infection.

2. Definitions

- *Enrolled*: From time consented to participate until designated as (i) ineligible based on the inclusion/exclusion criteria or withdraws, (ii) been discontinued from the study or (iii) completed the study.
- *Randomized*: when a randomization number is assigned.
- *Screen Failures*: signed informed consent, but then determined to be ineligible or withdraws before being randomized.
- *Discontinued*: randomized, but then withdrawn by investigator or subject withdraws consent
- *Completed:* Subjects are considered completed when they are followed through to day 28, had an adverse event or death occurred prior to day 28. Patients will be asked to have day 60 and day 90 study visits as well.

3. Study Population

Inclusion Criteria for Enrollment:

1. Patients ≥18 years of age
2. Hospitalized for COVID-19 respiratory symptoms
3. Hospitalized for less than 72 hours OR within day 3 to 7 from first signs of illness
4. Laboratory confirmed COVID-19
5. On supplemental oxygen, non-invasive ventilation or high-flow oxygen (clinical status 3 or 4)
6. N.B. It is assumed patients may be on other randomized controlled trials of pharmaceuticals for COVID -19 and patients who meet eligibility criteria will not be excluded on this basis.

Exclusion Criteria

1. Receipt of pooled immunoglobulin in past 30 days
2. Contraindication to transfusion or history of prior reactions to transfusion blood products
3. Invasive mechanical ventilation or extracorporeal membrane oxygenation (ECMO)
4. Volume overload secondary to congestive heart failure or renal failure

4. Subject Withdrawal

1. Subjects can terminate study participation and/or withdraw consent at any time without prejudice.
2. Randomized subjects who withdraw from the study will not be replaced.
3. The investigator may withdraw subjects if the investigator determines that continued participation in the study would be harmful to the subject or the integrity of the study data

4. Discontinuation of the study: The study sponsor, FDA and IRB all have the right to terminate this study at any time

Table 1: Schedule of Evaluations

**Study period**	**Screen**	**Baseline**	**Transfusion**	**Follow up**						
Day	-1 to 0	0	0	1	3	7	14	28	60	90
[redacted]										
Eligibility										
Informed consent	x									
Demographic and Medical history	x									
COVID-19 symptom screen	x									
SARS-CoV-2 RT-PCR for eligibility	x									
Pregnancy test	x									
ABO for plasma compatibility	x									
Chest imaging (CXRAY or CT scan), EKG	x[1]						x[1]			
Oxygenation Level	x									
Study Drug Administration										
Randomization		x								
Drug infusion			x							
Study Procedures										
Vital signs	x	x	xxxx[3]	x	x	x	x	x	x	x
Physical examination	x		x	x	x	x	x	x	x	x
Symptom screen	x	x	x	x	x	x	x	x	x	x
Concomitant medications	x	x	x							
Assessment with 5-point ordinal scale		x		x	x	x	x	x	x	x
Chest imaging (CXR or CT scan),					x					
Adverse event monitoring		x	x	x	x	x	x	x	x	x
Laboratory testing										
CBC and CMP		x		x	x	x	x			
PTT, LDH, fibrinogen, CRP, procalcitonin, ferritin		x		x	x	x	x			
Troponin		x		x	x	x	x			
SARS-CoV-2 RT-PCR[4]		x				x	x	x		
SARS-CoV-2 antibody		x	x[2]	x		x	x	x		x
IL-6, cytokines, CD4-CD8 counts		x		x		x	x	x		x

[3] Vital sign testing: Immediately prior to infusion, 10-20 minutes after start of infusion, at completion of infusion and 30-60 minutes after the end of the infusion

[4] Sites could include nasopharyngeal and throat.

[1] EKG, Chest imaging on admission or thereafter, before randomization. Repeat at 14 days or discharge, which ever comes first.
[2] Blood bank collects from plasma tail bag for antibody testing

5. Treatment

1. Subjects will be randomized in a 1:1 ratio to receive study drug (convalescent plasma) versus standard plasma; randomization will be stratified by site and risk.
2. Study drug: The investigational product is anti-SARS-CoV-2 convalescent plasma. Patients identified as having recovered from COVID-19 will serve as potential donors as per FDA guidelines (https://www.fda.gov/vaccines-blood-biologics/investigational-new-drug-ind-or-device-exemption-ide-process-cber/recommendations-investigational-covid-19-convalescent-plasma).
3. Potential donors and samples will be screened per New York Blood Center guidelines.
4. Treatment arm will receive 1-2 units of plasma with antibodies to SARS-CoV-2 per April 8, 2020 guidelines (https://www.fda.gov/vaccines-blood-biologics/investigational-new-drug-ind-or-device-exemption-ide-process-cber/recommendations-investigational-covid-19-convalescent-plasma).
5. Control arm will receive 1 unit of standard plasma
6. Both treatment and control plasma will be in standard plasma unit bags, with a study specific International Society of Blood Transfusion (ISBT) label

6. **Randomization:**

1. Subjects enrolled in the study will be randomized using an interactive web response system (IWRS) to receive study drug versus placebo at a 1:1 ratio with stratification by site and risk (high versus average).

7. Rationale for Dosing

Two units of plasma (approximately 250-500mL) containing anti-SARS2-CoV-19 antibodies and aim for a titer ≥1:160 per April 8, 2020 guidelines (https://www.fda.gov/vaccines-blood-biologics/investigational-new-drug-ind-or-device-exemption-ide-process-cber/recommendations-investigational-covid-19-convalescent-plasma).

We will utilize 2 units (~ 500 mL) of plasma with antibodies to SARS-CoV-as per April 8, 2020 FDA guidelines: https://www.fda.gov/vaccines-blood-biologics/investigational-new-drug-ind-or-device-exemption-ide-process-cber/recommendations-investigational-covid-19-convalescent-plasma. Dosing is based on experience with previous use of convalescent plasma therapy in SARS1 where 5 mL/kg of plasma at titer ≥ 1:160 was utilized (7). For a 70 Kg person plasma volume is estimated at 2800 mL (40 mL/kg x70 kg) with baseline anti-SARS-CoV-2 titer of 0. For example, if protective titer was 1:25 and each unit had titer of 1:160, ~300 mL can achieve this ([250/(2800+250)] x 1:160>1:25).

At the discretion of the treating physician, one unit may be administered if the patient is deemed to be at high risk of circulatory overload.

8. **Study drug administration**

1. Drug will be administered within 24 hours of randomization
2. Infusion rate ≤ 500 mL/hour

3. Pretreatment to minimize transfusion reactions (e.g. acetaminophen, diphenhydramine) may be given. Individual institutional guidelines/SOPs for the administration of plasma should be followed, including the use of any premeditations, such as acetaminophen and diphenhydramine.
4. If an AE develops during infusion, the infusion may be slowed or stopped as per investigator's decision.
   - Most reactions to plasma are relatively minor and the infusion can generally be continued. Infusion site burning and non-allergic systemic effects can generally be managed with slowing of the infusion. Infusion can generally be continued in cases of itching or hives after pausing the transfusion, administering antihistamines, and observing the patient for worsening.
   - Severe allergic reactions such as, bronchospasm and hypotension, generally require discontinuation of the infusion.
5. Concomitant medications will be documented on the Case Report Form (CRF)
   - Prescription medications
   - Over the counter medications
   - Herbal treatments/nutritional supplements
   - Blood products
   - Any medications with established activity against SARS-CoV-2 that subject is receiving

STATISTICAL PLAN

1. Sample Size and Power Considerations

The planned sample size for the trial is 300 subjects, randomized in a 1:1 ratio stratified by site and risk (high versus average) to receive anti-SARS-CoV-2 convalescent plasma versus control plasma. The primary analysis will compare the efficacy of anti-SARS-CoV-2 convalescent plasma using a proportional odds model. We estimated the sample size of the proportional odds model by simulations assuming a two-sided Type I error rate (alpha) of 0.1and 80% power. We made the following additional assumptions:

a. 30% incidence of worsening respiratory status (10% death and 20% on invasive mechanical ventilation or ECMO, respectively) and 10% of discharged alive in the control group estimated by current data from our hospital,
b. 1.7 odds ratio (OR) of worsening respiratory status between the control group and the anti-SARS-CoV-2 convalescent plasma group, This corresponds to an 11% absolute reduction in incidence of worsening respiratory status (6% death and 13% on invasive mechanical ventilation or ECMO, respectively) and 8% absolute increase of discharged alive using anti-SARS-CoV-2 convalescent plasma.
c. Very few subjects will be randomized and fail to receive study plasma infusion or will be lost to follow-up and have missing data for the primary endpoint.

We estimated a sample size of 300 patients (150 in each arm) would be sufficient to detect the specified difference in clinical status between the two arms with a power of at least 0.8.

2. Statistical Analysis

1.1 Primary endpoint:

Our primary hypothesis is that by providing anti-SARS-CoV-2 plasma, the proportional odds of worsening respiratory failure or death will be decreased as compared to the rate in the group receiving control plasma. We will apply a cumulative proportional odds model adjusting for initial status, site, and underlying risk. The general form of the cumulative proportional odds model will be: $\mathrm{logit}(\mathrm{P}(\mathrm{Y} \leq j) = \alpha_j + \beta X + \gamma C$, j=1,…,4, in which Y is the status, X is the treatment, C is a covariate, β and γ are the regression coefficients for the treatment and covariate. The $\exp(\beta)$ is the corresponding the OR ratio of treatment. All analyses will be conducted with a modified intention-to-treat approach, which excludes randomized subjects who do not initiate an infusion of the study plasma. In secondary analysis, we will

use inverse probability of selection weights to account for the individuals who did not initiate their assigned treatment. As secondary analysis, we will also analyze the reduced oxygenation rate, requirement for supplemental oxygen rate, and mechanical ventilation rate, respectively. Statistical inference will be based on a two-sided Type 1 error rate of 0.05 and 95% confidence intervals.

2. **Analysis of AE data**

Analysis of AE data will primarily be descriptive based on MedDRA coding of events. AE will be compared between randomized arms using Fisher's Exact Test.

3. **EXPLORATORY ANALYSIS**

3.1 Analysis of the anti-SARS-CoV-2 titers

Analysis of titers will primarily be descriptive, comparing the geometric mean titers at days 0, 7, 14, 28, 60 between the randomized arms. It is also of interest to describe the entire distributions of anti-SARS-CoV-2 titers by randomized arms and contrast these distributions. Therefore, we will use quantile regression to describe whether there is a shift or change in the titer distribution between randomized arms. Given that repeated measures of titers will be obtained, we will account for the correlation in measures within individuals using a cluster bootstrap in order to properly estimate the p-value and 95% confidence intervals. Similar analysis will also be applied to lymphocyte and neutrophil counts on days 0, 3, 7, 14 or as obtained in care, hematological measurements (D-dimer, fibrinogen) on days 0, 3, 7, 14 or as obtained in care, and T and B cell subsets on days 0, 7, 28.

3.2 Analysis the rates, levels and duration of SARS-CoV-2 RNA in NP swabs

This exploratory analysis will be primarily descriptive. The proportion positive at days 0, 1, 7, 28 and whether individuals lose positive status at a subsequent time. To determine the proportion that are positive, we will do a pooled complementary log-log model in order to describe the cumulative incidence of SARS-CoV-2 positivity over time. The pooled complementary log-log model is a discrete time-to-event-analysis that estimates the log hazard rate at each discrete time point. Like the analysis of anti-SARS-CoV-2 titers, the goal of this secondary aim is to describe the distribution of SARS-CoV-2 RNA between randomized arms. Therefore, we will use the same approach as for the anti-SARS-CoV-2 titers. Because the exact day that an individual becomes negative is not known, a minimum and maximum amount of positive time will be used to describe the positive duration of each individual. If the sample is adequate, we will describe the duration of positivity using a non-parametric approach for time-to-event analysis.

3.3 Mortality, in-hospital and Day 28; rates of ICU admission

Standard chi-square test will be applied to compare the two groups. Logistic regression will also be applied to adjust for the randomization stratification factors (age, immune compromised status, and comorbidity status) and any baseline variables that appear to be imbalanced across treatment arms despite randomization.

3.4 Lymphocyte and neutrophil counts on days 0, 3, 7, 14 or as obtained in care; Hematological measurements (D-dimer, fibrinogen) on days 0, 3, 7, 14 or as obtained in care; and T and B cell subsets on days 0, 7, 28, 90. We will use the same approach as above.

<u>STUDY PROCEDURES</u>

1. Study Protocol by Day:

Day -1 to 0:

A. Screening (must be completed before randomization)
B. Informed consent (obtained before performing study related activities)
C. Baseline Evaluation (at screening) (much of the information will be obtained from the medical record)

1. Demographics:
    - Age, sex, race
2. Medical history:
    - Timing of exposure to COVID-19 source patient
    - Acute and chronic medical conditions
    - Medications, allergies
    - Any medical condition arising after consent to be recorded as AE.
3. COVID-19 symptom screen:
    - Symptoms: Fevers, cough, shortness of breath.
    - History of illness: Onset of symptoms, source of contagion
4. Vital signs
5. COVID-19 testing (RT-PCR)
    - Nasopharyngeal, oropharyngeal, tracheal aspirate, bronchoalveolar lavage and/or stool (optional) samples
6. Baseline Basic Lab Testing and imaging
    - Blood typing, CBC, comprehensive metabolic panel
    - Chest imaging (CXRAY or CT scan), EKG
7. Serological testing: anti-SARS CoV-2 titers
8. Stored samples for future studies
9. Urine or serum pregnancy test
    - For females of childbearing potential
    - Results from laboratory tests obtained up to 7 days before enrollment may be used for the pregnancy test
10. Determination of eligibility
    - Inclusion/exclusion criteria age
    - Consent
    - Positive for COVID-19
    - Respiratory symptoms, not already an ICU patient
    - Between day 3 and 7 of first sign of illness or within 72 hours of admission

Day 0:

1. Randomization of eligible subject in IWRS
2. Study Plasma Administration:
    i. 1-2 units of plasma will be transfused
    ii. Time at start and end of infusion will be recorded
    iii. Vital signs will be measured immediately prior to infusion, 10-20 minutes after start of infusion, at completion of infusion and 30-60 minutes after the end of the infusion
    iv. Blood bank will collect plasma bag tail segment for SARS-CoV-2 and antibody titers
3. COVID-19 symptom screen: fevers, cough, shortness of breath
4. Assessment of clinical status (5-point ordinal scale)
5. New medical conditions, concomitant medication, AE evaluation
6. Physical examination
7. COVID-19 testing (RT-PCR): nasopharyngeal samples

8. Blood typing, CBC, comprehensive metabolic panel, C-reactive protein, PTT, LDH, fibrinogen, procalcitonin, troponins and CPK, IL6, cytokine panel, CD4-CD8 counts
9. Serological testing: anti-SARS CoV-2 titers
10.

<u>Day 1-7 (or for duration of hospitalization):</u>

1. Vital signs daily
2. COVID-19 symptom screen (fevers, cough, shortness of breath)
3. Assessment of clinical status (5-point ordinal scale)
4. New medical conditions, AE evaluation
5. Physical examination
6. CBC, comprehensive metabolic panel, CRP daily, PTT, LDH, fibrinogen, procalcitonin, troponin, CPK
7. Serological testing: anti-SARS CoV-2 titers
8. CXR (day 3) (or CT with increased oxygen requirement)
9. EKG (day 3 and 7 post plasma infusion)

<u>Day 14 (if still hospitalized):</u>

1. COVID-19 symptom screen (fevers, cough, shortness of breath)
2. Assessment of clinical status (5-point ordinal scale)
3. New medical conditions, AE evaluation
4. Physical examination
5. CBC, comprehensive metabolic panel, CRP, PTT, LDH, fibrinogen, procalcitonin, troponin, CPK, IL6, cytokine panel, CD4-CD8 counts
6. Serological testing: anti-SARS CoV-2 titers
7. Blood Direct PCR: SARS CoV-2

<u>Day 28 (if still hospitalized or as outpatient):</u>

1. COVID-19 symptom screen (fevers, cough, shortness of breath)
2. Assessment of clinical status (5-point ordinal scale)
3. New medical conditions, AE evaluation
4. Serological testing: anti-SARS CoV-2 titers
5. Blood Direct PCR: SARS CoV-2
6. Define disposition (home, hospital, status)
7. Pulmonary status (supplemental oxygen)

<u>Day 60 (as outpatient):</u>

1. COVID-19 symptom screen (fevers, cough, shortness of breath)
2. Assessment of clinical status (5-point ordinal scale)
3. New medical conditions, AE evaluation
4. Define disposition (home, hospital, status)
5. Pulmonary status (supplemental oxygen)

<u>Day 90 (as outpatient):</u>

1. COVID-19 symptom screen (fevers, cough, shortness of breath)
2. Assessment of clinical status (5-point ordinal scale)
3. New medical conditions, AE evaluation
4. Serological testing: anti-SARS CoV-2 titers
5. Blood Direct PCR: SARS CoV-2
6. Define disposition (home, hospital, status)
7. Pulmonary status (supplemental oxygen)

2. Efficacy, Virology, Serology and PK Measures:

Clinical Efficacy (ordinal scale):

1. Death/Cardiocirculatory arrest at anytime
2. Transfer to ICU
3. Type and duration of respiratory support (and other ICU support in ICU)
4. ICU mortality and LOS
5. Hospital mortality and LOS
6. Ventilator-free days
7. 28 day mortality

SARS-CoV-2 Viral and Antibody Response

1. Rates, levels and duration of SARS-CoV-2 RNA in NP swabs by RT-PCR
   - Day 0, 7, 14, 28
   - Other specimen types may be tested as available (*e.g.*, BAL fluid, tracheal secretions, sputum, etc.) or when RT-PCR assays are validated for additional sources (*i.e.*, stool, blood).
2. Serologic positivity and neutralization antibody titers for anti-SARS-CoV-2
   - Day 0, 7, 14, 28, 90

HUMAN SUBJECTS PROTECTIONS

1. RISK/BENEFIT ASSESSMENT

Known potential risks

a. A theoretical risk of administration of convalescent plasma is the phenomenon of antibody-mediated enhancement of infection (ADE). ADE can occur in viral diseases, such as dengue and involves an enhancement of disease in the presence of certain antibodies. For coronaviruses, several mechanisms of ADE have been described, including the theoretical concern that antibodies to one type of coronavirus could enhance infection to another strain (15). It may be possible to predict the risk of ADE in SARS-CoV-2 experimentally, as proposed for MERS (15). Since the proposed use of convalescent plasma in the COVID-19 epidemic would rely on preparations with high titers of antibody against the same virus, SARS2-CoV-2, ADE may be unlikely. Available evidence from the use of convalescent plasma in patients with SARS1 and MERS (16) demonstrated it is safe and there were no adverse effects in a pilot study of patients with COVID-19 (13). Nevertheless, caution and vigilance will be exercised to use clinical and laboratory measures to detect evidence of enhanced infection.

b. Another theoretical risk is that antibody administration to those exposed to SARS-CoV-2 may prevent disease but modify the immune response such that those who are treated may mount attenuated immune responses. This may leave them vulnerable to subsequent re-infection. Passive antibody administration before vaccination with respiratory syncytial virus attenuated humoral but not cellular immunity (17). This will be investigated as part of this clinical trial by comparing immune responses in those who receive standard plasma and convalescent plasma. If responses differ, those with attenuated levels could be vaccinated against COVID-19 when a vaccine becomes available. Nonetheless, these concerns are modest compared to the possible benefit of reducing the risk of respiratory failure and avoiding mechanical ventilation.

c. Finally, there are risks associated with any transfusion of plasma including transmission of transfusion transmitted viruses (e.g. HIV, HBV, HCV, etc.), allergic transfusion reactions, anaphylaxis to transfusion, febrile transfusion reaction, transfusion related acute lung injury (TRALI), transfusion associated cardiac overload (TACO), and hemolysis should ABO incompatible plasma be administered. To minimize the risks of disease transmission, all plasma will be screened for blood borne pathogens, and pathogen reduction techniques will be utilized to prepare the plasma using standardly accepted FDA guidelines that oversee plasma collection. In addition, donors will fulfill donor requirements which require a history of COVID19 illness, a positive COVID19 test, a two-week period of being asymptomatic post infection and a negative nasopharyngeal swab for SARS-CoV2 by PCR. These patients will be screened for high titers against SARS-CoV2 and referred to NYC Blood Bank or American Red Cross for apheresis (plasma donation).

<u>Known potential benefits</u>

The most important potential benefit is that convalescent plasma may reduce progression to respiratory failure in patients with COVID-19 and early respiratory symptoms, such as shortness of breath, cough, chest pain, and pulmonary infiltrates. The benefit of plasma is expected to an improvement in symptoms, oxygenation, the need for mechanical ventilation and possibly reduced mortality. Based on historical experience with antibody administration, antibody administration is expected to be effective relatively early in disease (1). Convalescent plasma was safe, reduced symptoms, and improved oxygenation in a non-randomized open label study of patients with more advanced disease in Wuhan, China (13).

Assessment of potential risks and benefits

Given historical data showing convalescent plasma was safe and possibly effective in patients with SARS1 (7, 16), and emerging data from China suggest it is safe and possibly effective in patients with severe Covid-19 along with the relative lack of other readily available therapeutic options for severe or life-threatening disease, the benefits of its use in those at high risk for severe disease outweigh the risks. However, for all patients in whom convalescent plasma administration is considered, a risk-benefit assessment will be conducted to assess individual variables. This protocol proposes a randomized controlled trial to assess the efficacy of convalescent plasma in preventing respiratory progression in patients with Covid-19. A recent JAMA editorial by experts note the importance of randomized clinical trials to demonstrate efficacy of this approach and change the course of the epidemic (18).

In pregnant woman and fetus there are limited data on potential risks and/or benefits. Pregnancy can cause changes in the coagulation and fibrinolytic systems and convalescent plasma may potentially benefit individuals but providing the coagulation factors.

Alternatives: The alternative to participation in this study is routine care.

2. Safety measures

1. Safety Evaluations will assess for clinical and laboratory indices of reactions to high titer anti-SARS-CoV-2 plasma and determine if they are higher, lower or the same as standard plasma
2. Clinical evaluations: Vital signs and symptom screen on days 0-7, 14 and symptom screens on days 28, 60, and 90.
3. Laboratory evaluations to include chest radiography (chest x-rays and/or chest CT and/or ultrasound) Safety laboratory tests (ABO typing, urine or serum pregnancy testing, CBC, comprehensive metabolic panel, PTT, LDH, fibrinogen, CRP, procalcitonin, troponin and CPK) will be performed at the local CLIA-certified clinical laboratory on days 0-7 and 14 as specified by above plan.

3. Definitions

1. **Adverse Event (AE):** Any untoward medical occurrence in a clinical investigation subject who has received a study intervention and that does not necessarily have to have a causal relationship with the study product. An AE can, therefore, be any unfavorable and unintended sign (including an abnormal laboratory finding, for example), symptom, or disease temporally associated with the use of the study product, whether or not considered related to the study product.

2. **Serious Adverse Event (SAE):** Any adverse event that results in any of the following outcomes:
    1. Death
    2. Life-threatening (immediate risk of death)
    3. Prolongation of existing hospitalization
    4. Persistent or significant disability or incapacity
    5. Important medical events that may not result in death, be life threatening, or require intervention or escalation of care may be considered a serious adverse event when, based upon appropriate medical judgment, they may jeopardize the subject and may require medical or surgical intervention to prevent one of the outcomes listed in this definition. Examples of such medical events include allergic bronchospasm requiring intensive treatment in an emergency room or at home, blood dyscrasias or convulsions that do not result in inpatient hospitalization.

- **Unexpected Adverse Event (UAE):** An adverse reaction, the nature or severity of which is not consistent with the investigator's brochure.

- **Serious and Unexpected Suspected Adverse Reaction (SUSAR):** An adverse reaction, the nature of which is not consistent with the investigator's brochure with severity as defined by SAE above.

- **Unanticipated Problem (UP):** Unanticipated Problem that is not an Adverse Event (e.g. breaches of confidentiality, accidental destruction of study records, or unaccounted-for study drug).

- **Protocol Deviation:** Deviation from the IRB-approved study procedures. Designated serious and non-serious
    1. **Serious Protocol Deviation:** Protocol deviation that is also an SAE and/or compromises the safety, welfare or rights of subjects or others

4. Safety Reporting Requirements

Reporting Interval

All AEs and SAEs will be documented from the first administration of study product. All AEs and SAEs will be followed until resolution even if AEs extend beyond the study-reporting period. Resolution of an adverse event is defined as the return to pre-treatment status or stabilization of the condition with the expectation that it will remain chronic. At any time after completion of the study, if the investigator becomes aware of a SAE that is suspected to be related to study product.

Investigator's Assessment of Adverse Events

The determination of seriousness, severity, and causality will be made by an on-site investigator who is qualified (licensed) to diagnose adverse event information, provide a medical evaluation of adverse events, and classify adverse events based upon medical judgment. This includes but is not limited to physicians, physician assistants, and nurse practitioners.

Laboratory abnormalities will be reported as AEs if there is a 2 standard deviation increase above baseline.

Assessment of Seriousness

1. Event seriousness will be determined according to the protocol definition of an SAE
2. Assessment of Severity

Event severity will be assigned according to the scale below

1 = Mild: Transient or mild discomfort (<48 hours); no medical intervention/therapy required.
2 = Moderate: Some worsening of symptoms but no or minimal medical intervention/therapy required)
3 = Severe: Escalation of medical intervention/therapy required
4 = Life-threatening: Marked escalation of medical intervention/therapy required.
5 = Death

Assessment of Association

1. The association assessment categories that will be used for this study are:
   - **Associated** – The event is temporally related to the administration of the study product and no other etiology explains the event.
   - **Not Associated** – The event is temporally independent of the study product and/or the event appears to be explained by another etiology.

2. The investigator must provide an assessment of association or relationship of AEs to the study product based on:
   - Temporal relationship of the event to the administration of study product
   - Whether an alternative etiology has been identified
   - Biological plausibility
   - Existing therapy and/or concomitant medications.

5. Safety Oversight

Monitoring Plan

1. All AE and SAE will be reviewed by protocol team weekly, or more often if needed.

2. A medical monitor will be appointed by the study team for safety oversight of the clinical study.

3. A data safety monitoring board (DSMB) composed of independent experts, including infectious diseases and hematology specialists, without conflict of interests will be established. The DSMB reports will be disseminated to all other participating site at least annually. If there is any information that suggests the changed risk of the study or lack of benefit, the report will be distributed to other sites within 2 days. The DSMB will review the study before initiation, after enrollment of patient 10, patient 20, then at the midpoint of enrollment and at least yearly thereafter. The DSMB will review study data to evaluate the safety, enrollment, efficacy, study progress, and conduct of the study.

4. An Independent Safety Monitor (ISM) will be appointed. The ISM is a physician with expertise in infectious diseases and whose primary responsibility is to provide timely independent safety monitoring. An ISM is in close proximity to the study site and has the authority to readily access study participant records. The ISM reviews any SAE that occurs at the study site in real time and provides a written assessment to DMID.

6. Study monitoring

1. As per ICH-GCP 5.18 and FDA 21 CFR 312.50, clinical protocols are required to be adequately monitored. The study team will verify that
   a. There is documentation of the informed consent process and signed informed consent documents for each participant
   b. There is compliance with recording requirements for data points
   c. All SAEs are reported as required
   d. Individual participant study records and source documents align
   e. Investigators are in compliance with the protocol
   d. Regulatory requirements as per Office for Human Research Protections (OHRP), FDA, and applicable guidelines (ICH-GCP) are being followed.

<u>STUDY MODIFICATION</u>

1. Halting Criteria for the Study: The study enrollment and dosing will be stopped and an ad hoc review will be performed if any of the specific following events occur or, if in the judgment of the study physician, participant safety is at risk of being compromised:

1. Death within one hour of plasma infusion

2. Occurrence of a life-threatening allergic/hypersensitivity reaction (anaphylaxis), manifested by bronchospasm with or without urticaria or angioedema requiring hemodynamic support with pressor medications or mechanical ventilation, TRALI, TACO

3. One participant with an SAE associated with study product.

4. Two participants with a Grade 3 or higher lab toxicity for the same parameter associated with study product. (Grading will be assessed using Common Terminology Criteria for Adverse Events (CTCAE) grading scale developed by NCI, NIH , https://evs.nci.nih.gov/ftp1/CTCAE/About.html)

5. An overall pattern of symptomatic, clinical, or laboratory events that the medical monitor, or DSMB consider associated with study product and that may appear minor in terms of individual events but that collectively may represent a serious potential concern for safety.

6. Any other event(s) which is considered to be a serious adverse event in the good clinical judgment of the responsible physician. This will be appropriately documented.

2. Halting Criteria/Rules for Subject Infusion: Infusion of study drug will be halted if any of the following manifestations of anaphylaxis develop and will not be restarted:

- Skin or mucous membrane manifestations: hives, pruritus, flushing, swollen lips, tongue or uvula
- Respiratory compromise: dyspnea, wheezing, stridor, hypoxemia
- A decrease in systolic blood pressure to < 90 mmHg or >30% decrease from baseline or a diastolic drop of >30% from baseline.
- Tachycardia with an increase in resting heart rate to > 130 beats per minute; or bradycardia <40 that is associated with dizziness, nausea or feeling faint.
- Any other symptom or sign which in the good clinical judgment of the study clinician or supervising physician warrants halting the infusion. For example, the rapid onset of gastrointestinal symptoms, such as nausea, vomiting, diarrhea, and cramps, for instance, may be manifestations of anaphylaxis and may warrant an immediate halt prior to meeting full SAE criteria

ETHICS/PROTECTION OF HUMAN SUBJECTS

1. Ethical Standard

All sites conducting this study are committed to the integrity and quality of the clinical studies it coordinates and implements according with local institutional and regulatory requirements.

All sites participating in this research have a Federal wide Assurance (FWA) number on file with the Office for Human Research Protections (OHRP).

This assurance commits a research facility to conduct all human subjects' research in accordance with the ethical principles in The Belmont Report and any other ethical standards recognized by OHRP. Finally, per OHRP regulations, the research facility will ensure that the mandatory renewal of this assurance occurs at the times specified in the regulations.

2. Institutional Review Board

The Albert Einstein College of Medicine IRB will review this protocol and all protocol-related documents and procedures as required by OHRP and local requirements before subject enrollment.

3. **Informed Consent Process**

Each site will follow institution specific policy and process for consenting participants of legally authorized representatives for the study. A site specific protocol addendum will be provided by each site to the respective IRB.

The informed consent process will be initiated before a volunteer agrees to participate in the study and should continue throughout the individual's study participation. The subject will sign the informed consent document before any procedures are undertaken for the study. A copy of the signed informed consent document will be given to the subject for their records. The consent will explain that subjects may withdraw consent at any time throughout the course of the trial. Extensive explanation and discussion of risks and possible benefits of this investigation will be provided to the subjects in understandable language. Adequate time will be provided to ensure that the subject has time to consider and discuss participation in the protocol. The consent will describe in detail the study interventions/products/procedures and risks/benefits associated with participation in the study. The rights and welfare of the subjects will be protected by emphasizing that their access to and the quality of medical care will not be adversely affected if they decline to participate in this study.

3. Subject Confidentiality

Subject confidentiality is strictly held in trust by the participating investigators, their staff, and the sponsors and their agents. No information concerning the study or the data will be released to any unauthorized third party without prior written approval of the sponsor. The results of the research study may be published, but subjects' names or identifiers will not be revealed. Records will remain confidential. To maintain confidentiality, the PI will be responsible for keeping records in a locked area and results of tests coded to prevent association with subjects' names. Data entered into computerized files will be accessible only by authorized personnel directly involved with the study and will be coded. Subjects' records will be available to the FDA, the New York Blood Center and their representatives, investigators at the site involved with the study, and the IRB.

4. Future Use of Stored Specimens

Subjects will be asked for consent to use their samples for future testing before the sample is obtained. The confidentiality of the subject will be maintained. There will be no plans to re-contact them for consent or to inform them of results. Each specimen will be assigned a unique identifier at the time of collection. The identifier links the information it contains to the labeled specimen. The risk of collection of the sample will be the small risk of bruising or fainting associated with phlebotomy however these samples will be taken at the same time as other protocol required samples. No human genetic testing will be performed on the samples. Five ml of blood samples will be collected at 5 time points (See Schedule of Events). Serum and PBMCs collected at Albert Einstein will be frozen in aliquots and stored in Dr. Pirofski's laboratory freezer at Albert Einstein College of Medicine as de-identified, coded samples. Studies on these samples will answer important questions on the antibody response to SARS-CoV-2 that will inform future therapeutic development, interventions, and vaccine development. For example, it is unknown which type of antibody will provide protection. Studies on these patients' antibodies and immune responses may help determine the best viral antigen to target, provide insights into the role of neutralizing versus non neutralizing antibody; identify minimum required titers and functional attributes of antibodies needed to achieve a clinical response. Similarly, COVID19 protective cellular responses are unknown. The analysis of these samples will allow immunophenotyping studies to characterize the functional state of cellular responses associated with clinical outcomes and antibody responses. The goal of these studies is to fill information gaps to inform the design of future therapeutics such as vaccines, monoclonal antibodies and immune modulators. These immune assays can also be used in the future for more granular prognostic indicators. If for instance, there were unanticipated AEs, serum could be used to run tests that might help determine the reason for the AEs. Cytokines could be measured, for example.

Samples will not be shared with investigators other than investigators included in this protocol. The linking key to re-identify specimens will be held locally by the site. The specimens will remain linked and at Montefiore Medical Center for 5 years. Storage beyond the study length will be an option. Subjects who change their mind and no longer want their samples stored for future use can write to the local site PI to request destruction of their samples. Any use of these specimens not specified in the current protocol will be reviewed by the Einstein IRB.

5. Data management and monitoring

a. Source Documents

The primary source documents for this study will be the subjects' medical records. If the investigators maintain separate research records, both the medical record and the research records will be considered the source documents for the purposes of auditing the study. The investigator will retain a copy of source documents. The investigator will permit monitoring and auditing of these data, and will allow the IRB and regulatory authorities access to the original source documents. The investigator is responsible for ensuring that the data collected are complete, accurate, and recorded in a timely manner. Source documentation (the point of initial recording of information) should support the data collected and entered in to the study database and must be signed and dated by the person recording and/or reviewing the data. All data submitted should be reviewed by the site investigator and signed as required with written or electronic signature, as appropriate. Data entered into the study database will be collected directly from subjects during study visits or will be abstracted from subjects' medical records. The subjects' medical records must record their participation in the clinical trial and what medications (with doses and frequency) or other medical interventions or treatments were administered, as well as any AEs experienced during the trial.

b. Data Management Plan

Study data will be collected at the study site(s) and entered into the study database. Data entry is to be completed on an ongoing basis during the study.

c. Data Capture Methods

Clinical data will be entered into a 21 CFR 11-compliant Internet Data Entry System (IDES). The data system includes password protection and internal quality checks to identify data that appear inconsistent, incomplete, or inaccurate.

d. Study Record Retention

The PI is responsible for retaining all essential documents listed in the ICH GCP Guidelines. The FDA requires study records to be retained for up to 2 years after marketing approval or disapproval (21 CFR 312.62), or until at least 2 years have elapsed since the formal discontinuation of clinical development of the investigational agent for a specific indication. These records are also to be maintained in compliance with IRB/IEC, state, and federal medical records retention requirements, whichever is longest. All stored records are to be kept confidential to the extent provided by federal, state, and local law.

No study document should be destroyed. Should the investigator wish to assign the study records to another party and/or move them to another location, the site investigator must provide written notification of such intent to sponsor with the name of the person who will accept responsibility for the

transferred records and/or their new location. The sponsor must be notified in writing and written permission must be received by the site prior to destruction or relocation of research records.

Typical features according to current publications
Age Mean (SD) 55,5 (13·1), Male (68%)
Exposure to Huanan seafood market in Wuhan, China (49%)
Chronic medical underlying illness (51%)
Admission to Intensive Care Unit (23%)

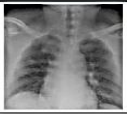

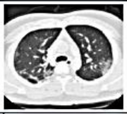

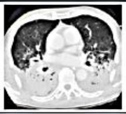

INCUBATION PERIOD and ONSET OF SYMPTOMS 3 DAYS AGO

	FIRST WEEK				SECOND WEEK				
SETTING	WARD Illness day 4	WARD Illness day 5	WARD Illness day 6	WARD Illness day 7	WARD/ICU Illness day 8	ICU Illness day 9	ICU Illness day 10	ICU Illness day 11	LONG TERM INFO PENDING
REPEATED SAMPLING OF THE NASOPHARYNX AND TRACHEAL ASPIRATES (IF INTUBATED) BY rRT-PCR FOR THE COVID-19	Initial important viral shedding		Decrease of the viral shedding sometimes associated with transient respiratory deterioration		Respiratory failure, increase of the viral shedding and viremia or Decrease of the viral shedding, and superinfections			Duration of viral excretion unknown	
OXYGEN THERAPY AND MECHANICAL VENTILATION	NO		Consider oxygen support	FNC	FNC followed by MV	MV		MV	
ORGAN FAILURE	**Typical signs according to current publications** Fever, cough, and shortness of breath (15%) bilateral pneumonia (75%), lymphopenia (35%), thrombocytopenia (12%), prothrombin time decreased (30%), elevated liver enzyme levels (about 30%)		Deterioration of respiratory status with most often spontaneous recovery		ARDS If shock beware of superinfections ⚠ Possible renal failure Neurological failure unlikely Hemostasis disorders			YES	
CO-INFECTION/SUPERINFECTION	NOT LIKELY				Consider a possible HAP/VAP and other nosocomial infections (see text for diagnostic procedures)			Profound immune paralysis and late onset infections	
ANTIBIOTICS	NO				Consider antibiotic therapy			YES	
ANTIVIRAL AGENTS	NO				Consider antiviral agents if deterioration[a]				

FNC = flow nasal cannula; HFNC = high flow nasal cannula; HAP = healthcare-associated pneumonia; VAP = ventilator-associated pneumonia; MV = Mechanical ventilation;
[a] The use of immunomodulation including corticosteroids is unlikely but debated

Figure 1 *Describes what is known about the potential course of patients with COVID-19 pneumonia. The goal of this protocol is to administer convalescent plasma in the "green" area to evaluate its ability to improve the clinical respiratory status of the patient and avoid the need for respiratory support, mechanical ventilation and/or ICU admission. (from Bouadma et al Int Care Med 2020)*

PHYSIOLOGICAL PARAMETERS	3	2	1	0	1	2	3
Respiration Rate	≤8		9 - 11	12 - 20		21 - 24	≥25
Oxygen Saturations	≤91	92 - 93	94 - 95	≥96			
Any Supplemental Oxygen		Yes		No			
Temperature	≤35.0		35.1 - 36.0	36.1 - 38.0	38.1 - 39.0	≥39.1	
Systolic BP	≤90	91 - 100	101 - 110	111 - 219			≥220
Heart Rate	≤40		41 - 50	51 - 90	91 - 110	111 - 130	≥131
Level of Consciousness				A			V, P, or U

Figure 2 NEWS score

REFERENCES

1. Casadevall A, Pirofski LA. 2020. The convalescent sera option for containing COVID-19. J Clin Invest doi:10.1172/JCI138003.
2. Casadevall A, Scharff MD. 1994. Serum therapy revisited: animal models of infection and development of passive antibody therapy. Antimicrob Agents Chemotherap 38:1695-1702.
3. Casadevall A, Dadachova E, Pirofski LA. 2004. Passive antibody therapy for infectious diseases. Nat Rev Microbiol 2:695-703.
4. Zhang JS, Chen JT, Liu YX, Zhang ZS, Gao H, Liu Y, Wang X, Ning Y, Liu YF, Gao Q, Xu JG, Qin C, Dong XP, Yin WD. 2005. A serological survey on neutralizing antibody titer of SARS convalescent sera. J Med Virol 77:147-50.
5. Sahr F, Ansumana R, Massaquoi TA, Idriss BR, Sesay FR, Lamin JM, Baker S, Nicol S, Conton B, Johnson W, Abiri OT, Kargbo O, Kamara P, Goba A, Russell JB, Gevao SM. 2017. Evaluation of convalescent whole blood for treating Ebola Virus Disease in Freetown, Sierra Leone. J Infect 74:302-309.
6. Casadevall A, Pirofski L. 2003. The damage-response framework of microbial pathogenesis. Nat Rev Microbiol 1:17-24.
7. Cheng Y, Wong R, Soo YO, Wong WS, Lee CK, Ng MH, Chan P, Wong KC, Leung CB, Cheng G. 2005. Use of convalescent plasma therapy in SARS patients in Hong Kong. Eur J Clin Microbiol Infect Dis 24:44-6.
8. Yeh KM, Chiueh TS, Siu LK, Lin JC, Chan PK, Peng MY, Wan HL, Chen JH, Hu BS, Perng CL, Lu JJ, Chang FY. 2005. Experience of using convalescent plasma for severe acute respiratory syndrome among healthcare workers in a Taiwan hospital. J Antimicrob Chemother 56:919-22.
9. Ko JH, Seok H, Cho SY, Ha YE, Baek JY, Kim SH, Kim YJ, Park JK, Chung CR, Kang ES, Cho D, Muller MA, Drosten C, Kang CI, Chung DR, Song JH, Peck KR. 2018. Challenges of convalescent plasma infusion therapy in Middle East respiratory coronavirus infection: a single centre experience. Antivir Ther 23:617-622.
10. Arabi YM, Hajeer AH, Luke T, Raviprakash K, Balkhy H, Johani S, Al-Dawood A, Al-Qahtani S, Al-Omari A, Al-Hameed F, Hayden FG, Fowler R, Bouchama A, Shindo N, Al-Khairy K, Carson G, Taha Y, Sadat M, Alahmadi M. 2016. Feasibility of Using Convalescent Plasma Immunotherapy for MERS-CoV Infection, Saudi Arabia. Emerg Infect Dis 22:1554-61.
11. van Erp EA, Luytjes W, Ferwerda G, van Kasteren PB. 2019. Fc-Mediated Antibody Effector Functions During Respiratory Syncytial Virus Infection and Disease. Front Immunol 10:548.
12. Gunn BM, Yu WH, Karim MM, Brannan JM, Herbert AS, Wec AZ, Halfmann PJ, Fusco ML, Schendel SL, Gangavarapu K, Krause T, Qiu X, He S, Das J, Suscovich TJ, Lai J, Chandran K, Zeitlin L, Crowe JE, Jr., Lauffenburger D, Kawaoka Y, Kobinger GP, Andersen KG, Dye JM, Saphire EO, Alter G. 2018. A Role for Fc Function in Therapeutic Monoclonal Antibody-Mediated Protection against Ebola Virus. Cell Host Microbe 24:221-233 e5.
13. Duan K, Liu B, Li C, Zhang H, Yu T, Qu J, Zhou M, Chen L, Meng S, Hu Y, Peng C, Yuan M, Huang J, Wang Z, Yu J, Gao X, Wang D, Yu X, Li L, Zhang J, Wu X, Li B, Xu Y, Chen W, Peng Y, Hu Y, Lin L, Liu X, Huang S, Zhou Z, Zhang L, Wang Y, Zhang Z, Deng K, Xia Z, Gong Q, Zhang W, Zheng X, Liu Y, Yang H, Zhou D, Yu D, Hou J, Shi Z, Chen S, Chen Z, Zhang X, Yang X. 2020. Effectiveness of convalescent plasma therapy in severe COVID-19 patients. Proceedings of the National Academy of Sciences doi:10.1073/pnas.2004168117:202004168.
14. Shen C, Wang Z, Zhao F, Yang Y, Li J, Yuan J, Wang F, Li D, Yang M, Xing L, Wei J, Xiao H, Yang Y, Qu J, Qing L, Chen L, Xu Z, Peng L, Li Y, Zheng H, Chen F, Huang K, Jiang Y, Liu D, Zhang Z, Liu Y, Liu L. 2020. Treatment of 5 Critically Ill Patients With COVID-19 With Convalescent Plasma. JAMA doi:10.1001/jama.2020.4783.

15. Wan Y, Shang J, Sun S, Tai W, Chen J, Geng Q, He L, Chen Y, Wu J, Shi Z, Zhou Y, Du L, Li F. 2020. Molecular Mechanism for Antibody-Dependent Enhancement of Coronavirus Entry. J Virol 94.
16. Mair-Jenkins J, Saavedra-Campos M, Baillie JK, Cleary P, Khaw FM, Lim WS, Makki S, Rooney KD, Nguyen-Van-Tam JS, Beck CR, Convalescent Plasma Study G. 2015. The effectiveness of convalescent plasma and hyperimmune immunoglobulin for the treatment of severe acute respiratory infections of viral etiology: a systematic review and exploratory meta-analysis. J Infect Dis 211:80-90.
17. Crowe JE, Jr., Firestone CY, Murphy BR. 2001. Passively acquired antibodies suppress humoral but not cell-mediated immunity in mice immunized with live attenuated respiratory syncytial virus vaccines. J Immunol 167:3910-8.
18. Roback JD, Guarner J. 2020. Convalescent Plasma to Treat COVID-19: Possibilities and Challenges. JAMA doi:10.1001/jama.2020.4940.

CONVALESCENT PLASMA TO LIMIT CORONAVIRUS ASSOCIATED COMPLICATIONS: A RANDOMIZED BLINDED PHASE 2 STUDY COMPARING THE EFFICACY AND SAFETY OF ANTI-SARS-COV-2 PLASMA TO PLACEBO IN COVID-19 HOSPITALIZED PATIENTS (CONTAIN COVID-19)

Protocol Version: 3.2

Protocol Date: April 4, 2021

IND 20427

ClinicalTrials.gov Identifier: NCT 04364737

LIST OF ABBREVIATIONS

ADR: Adverse Drug Reaction
ADE: Antibody-mediated enhancement of infection
AE: Adverse Event/Adverse Experience
AFib: Atrial fibrillation
CBC: Complete Blood Count
CDC: United States Centers for Disease Control and Prevention
CFR: Code of Federal Regulations
CHF: Chronic heart failure
CLIA: Clinical Laboratory Improvement Amendment of 1988
COI: Conflict of Interest
COPD: Chronic obstructive pulmonary disease
COVID-19: Coronavirus Disease
CRF: Case Report Form
CRP: C-Reactive Protein
CP: Convalesced Plasma
DMC: Data Management Center
DSMB: Data and Safety Monitoring Board
EUA: Emergency Use Authorization
FDA: Food and Drug Administration
GCP: Good Clinical Practice
HBV: Hepatitis B virus
HCV: Hepatitis C virus
HFNC: High flow nasal cannula
HIV: Human immunodeficiency virus
HTLV: Human T-cell lymphotropic virus
IB: Investigator's Brochure
ICF: Informed Consent (Informed Consent Form)
ICH: International Conference on Harmonization
ICU: Intensive Care Unit
IEC: Independent Ethics Committee
ILD: Interstitial lung disease
IND: Investigational New Drug Application
IRB: Institutional Review Board
ISBT: International Society of Blood Transfusion
ISM: Independent Safety Monitor
IWRS: Interactive Web Response System
LOS: Length of Stay
MERS: Middle East Respiratory Syndrome
NP: Nasopharyngeal
NYBC: New York Blood Center
SS: Saline Solution (defined as half-, quarter- or normal saline)
OHRP: Office of Human Research Protections
OP: Oropharyngeal
OSA: Obstructive sleep apnea
RT-PCR: Reverse Transcriptase Real-Time Polymerase Chain Reaction
PK: Pharmacokinetic
SAE: Serious Adverse Event
SAP: Statistical Analysis Plan
SARS: Severe Acute Respiratory Syndrome

SARS-CoV-2: Severe Acute Respiratory
TACO: Transfusion-associated Circulatory Overload
T. cruzi: *Trypanosoma cruzi*
TRALI: Transfusion-related Acute Lung Injury
UP: Unanticipated Problem
UPnonAE: Unanticipated Problem that is not an Adverse Event

PROTOCOL SUMMARY

Long title: Convalescent Plasma to Limit Coronavirus Associated Complications: A Randomized Blinded Phase 2 Study Comparing the Efficacy and Safety of Anti-SARS-CoV-2 Plasma to Placebo in COVID-19 hospitalized patients (CONTAIN COVID-19)

Clinical Phase: 2 Blinded

Sample Size: Enrollment will be adapted based on continuous Bayesian monitoring as detailed in the SAP. The estimated total sample size for the trial is >1000 subjects

Study Population: Hospitalized COVID-19 patients aged ≥18 years of age with respiratory symptoms within 7 days from the onset of illness OR within 3 days of hospitalization.

Study Composition: NYU Langone Heath will serve as the Clinical and Data Coordinating Center for the study under IND 20427. Collaborating sites will contribute recruitment and conduct the study per the FDA approved protocol with oversight by their individual IRBs.

Study Duration: April 17, 2020 to January 31, 2023

Study Design: This trial design is built as a process with the possibility of multiple interventions being investigated. The trial is designed to be flexible, and these flexible aspects are designed and planned as part of the protocol. This trial may incorporate a flexible number of interventions, with the possibility of these numbers evolving as the science evolves. Each period of the study where intervention arms are added or dropped will be considered a separate study *Stage*, though the model will analyze all stages simultaneously.

Study Stages and Interventions: The first stage of the study has been determined. In Stage 1, there will be two intervention arms: (1) placebo control, Saline Solution (SS is defined as half-,quarter-, or normal saline), and (2) SARS-CoV-2 Convalescent Plasma (1 unit). Stage 2 has not yet been defined given that results from Stage 1 will guide how to structure the two arms for Stage 2 and subsequent Stages. Once each study Stage is completed and prior to initiating the subsequent study Stage, a protocol modification describing the specific arms and approach will be made to the IRB for review and approval.

Randomization: Randomization assignments are performed for patients at baseline. Randomization is performed separately by two strata, treatment site and risk of severe disease (high versus lower) as defined below. The randomization scheme will be determined by the study *Stage* and its associated group of intervention arms (For example, in Stages 1 and 2, subjects will be randomized to 1:1 ratio). Randomization should obviate the need for additional adjustment factors but if pre-specified demographic or clinical characteristics are unbalanced with respect to treatment group, we will consider adjustment; these characteristics include but are not limited to age, sex, race, ethnicity, BMI and COVID severity at baseline.

Risk for severe COVID-19 based on baseline characteristics:

- <u>High risk</u>: Subjects with age ≥60 years or, age <60 and at least one of the following:
  - Chronic pulmonary conditions (COPD, OSA, ILD, etc)
  - Chronic heart conditions (CHF with NYHA>= class 2, AFib, ischaemic heart disease, etc)
  - Hypertension
  - Chronic kidney disease with eGFR < 60 mL/min
  - Body Mass Index >=35
  - Diabetes mellitus

- Immunosuppression (CD4<200, on immunosuppressive medications for autoimmune conditions, cancers, solid or stem cell transplants, steroids such as prednisone >10mg/day or equivalent)

- Lower risk: Subjects with age <60 and without the presence of any high risk factors listed above.

Data Collection:
The following will be assessed in all subjects: (detailed schedule of assessments is found in Table 1)
I. Clinical, Laboratory and Imaging Data

1. Date of Symptom Onset and history of presenting illness
2. Demographics: Age, sex, comorbidities, zip code, race/ethnicity, BMI
3. Vital Signs: Temperature, respiratory rate, blood pressure, oxygen saturation, oxygen requirements
4. Laboratory Data:
    - Hematologic Markers: CBC with differential (neutrophil, lymphocyte counts and platelet count explicitly recorded), PTT, LDH, D-dimer, fibrinogen, ferritin
    - Metabolic Markers: Complete metabolic panel, LFT
    - Cardiac Markers: Troponin, (pro-)BNP
    - Inflammatory Markers: CRP, procalcitonin
5. Chest imaging (CT or Chest x-ray), EKG, echocardiogram if done: Day 0, Day 3, and Day 14 or discharge whichever comes first and times obtained as part of standard care
6. Venous duplex of lower extremities as clinically indicated
7. Evaluation for pulmonary embolism as clinically indicated

II. Safety and Efficacy

1. Day 0 (baseline), 1, 2, 3, 7, 14, and 28 and once at 2-3 months.

III. SARS-CoV-2 Viral and Antibody Response – to be done at selected Sites

1. Serum or plasma antibody titer[1] to SARS-CoV-2: Day 0, 1, 7, 14, 28, 90
2. SARS-CoV-2 PCR from nasopharyngeal swab, quantitative if available: Day 0, 7, 14, 28, 90

IV. Outcome measures:
Primary Outcome: Status at 14 days using the WHO 11-point ordinal scale for clinical improvement which ranges from 0 (uninfected) to 10 (death). Effect size will be measured as the cumulative odds ratio comparing treatment to placebo control, estimated using a cumulative proportional odds model that adjusts for initial status (indicator for status = 5 or status = 6).

Study Product:

- SARS-CoV-2 CP (1-2 units; ~250-500 mL with antibodies to SARS-CoV-2[1] per May 1, 2020 directive https://www.fda.gov/vaccines-blood-biologics/investigational-new-drug-ind-or-device-exemption-ide-process-cber/recommendations-investigational-covid-19-convalescent-plasma, obtained from New York Blood Center (NYBC) collected per their protocol (https://www.nybc.org/donate-blood/covid-19-and-blood-donation-copy/convalescent-plasma/).
- Equivalent volume of SS

Primary Objective: Evaluate the efficacy of convalescent plasma from people who have recovered from COVID-19 containing antibodies to SARS-CoV-2 versus control (SS) to prevent worsening respiratory status or death in hospitalized patients with COVID-19 who are within 3 days of presentation to the hospital or within 7 days of symptom onset.

[1] Vetted for SARS-CoV-2 IgG by platform used by NYBC.

Primary Endpoint:
Primary Outcome: Status at 14 days using the WHO 11-point ordinal scale for clinical improvement which ranges from 0 (uninfected) to 10 (death). Effect size will be measured as the cumulative odds ratio comparing treatment to placebo control, estimated using a cumulative proportional odds model that adjusts for initial status (indicator for status = 5 or status = 6).

WHO ordinal scale for clinical improvement

Patient State	Score	Descriptor
Uninfected	0	Uninfected; no viral RNA detected
Ambulatory	1	Asymptomatic; viral RNA detected
	2	Symptomatic; Independent
	3	Symptomatic; assistance needed
Hospitalized: Mild disease	4	Hospitalized; no oxygen therapy
	5	Hospitalized; oxygen by mask or nasal prongs
Hospitalized: Severe disease	6	Hospitalized; oxygen by NIV or High flow
	7	Intubation & Mechanical ventilation; pO2/FIO2 >/= 150 or SpO2/FIO2 >/=200
	8	Mechanical ventilation pO2/FIO2 < 150 (SpO2/FIO2 <200) or vasopressors
	9	Mechanical ventilation pO2/FIO2 < 150 and vasopressors, dialysis or ECMO
Death	10	Dead

Secondary Objectives:
Secondary outcome; same as above at 28 days.

Exploratory objectives (To be performed on selected sites):
1. Evaluation of the anti-SARS-CoV-2 titers in convalescent (donor) and patient serum or plasma
2. Analysis of SARS-CoV-2 antibody profiles and functional assays in convalescent (donor) and patient serum or plasma
3. Analysis of the, levels of SARS-CoV-2 RNA in NP swabs
4. Analysis of SARS CoV2 sequences
5. Clinical status (WHO score, symptoms) at other visit days, mortality, and rates of discharge
6. Comparison of selected labs at various visits
7. Assessment of role of concomitant medications in clinical outcome

Safety objectives:
- Safety monitoring will be per DSMB

Study population:
Inclusion Criteria
1. Patients ≥18 years of age
2. Hospitalized with laboratory confirmed COVID-19
3. One or more of the following respiratory signs or symptoms: cough, chest pain, shortness of breath, fever, oxygen saturation ≤ 94%, abnormal CXR/CT imaging
4. Hospitalized for ≤ 72 hours OR within 7 days from first signs of illness
5. On supplemental oxygen, non-invasive ventilation or high-flow oxygen
6. Patients may be on other randomized controlled trials of pharmaceuticals for COVID -19 and patients who meet eligibility criteria will not be excluded on this basis.

Exclusion Criteria

1. Receipt of any Covid-19 vaccine or participation in a Covid-19 vaccine study as a subject.
2. Receipt of pooled immunoglobulin in past 30 days
3. Contraindication to transfusion or history of prior reactions to transfusion blood products
4. Invasive mechanical ventilation or extracorporeal membrane oxygenation (ECMO)
5. Volume overload secondary to congestive heart failure or renal failure
6. Unlikely to survive past 72 hours from screening based on the assessment of the investigator
7. Unlikely to follow-up or clinical assessment will be hindered by patient's poor functional status

RATIONALE/BACKGROUND:

1. **Background and scientific rationale**

There are currently no proven treatment options for coronavirus disease (COVID-19) and the related pneumonia, caused by Severe Acute Respiratory Syndrome Coronavirus 2 (SARS-CoV-2) beyond supportive care. Human convalescent plasma is a treatment option for COVID-19 and could be rapidly available when there are enough people who have recovered and donate high titer (anti- SARS-CoV-2) neutralizing immunoglobulin-containing plasma. As more individuals contract COVID-19 and recover, the number of potential donors will continue to increase, to allow greater use.

Use of convalescent plasma is a form of passive antibody therapy that involves the administration of antibodies to a given agent to a susceptible individual for the purpose of preventing or treating the infectious disease it causes. In contrast, active vaccination requires the induction of an immune response that takes time to develop and varies in efficacy depending on the vaccine recipient. Some immunocompromised patients fail to achieve an adequate immune response to active vaccination and at present, there are no vaccines to prevent COVID-19. When given to a susceptible person, antibody used for therapy will circulate in the blood, reach tissues and hopefully mediate a beneficial effect by anti-microbial anti-inflammatory activity (1). Depending on antibody amount and composition, protection conferred by transferred immunoglobulin can last from weeks to months.

Passive antibody administration is the only means of providing immediate immunity to susceptible persons and immunity of any measurable kind for susceptible, including immunocompromised patients with COVID-19. This kind of antibody therapy has a storied history going back to the 1890s and was the only means of treating certain infectious diseases prior to the development of antimicrobial therapy in the 1940s (2, 3). Experience from prior outbreaks with other coronaviruses, such as SARS-CoV-1 shows that such convalescent plasma contains neutralizing antibodies to the relevant virus (4). In the case of SARS-CoV-2, the anticipated mechanism of action by which passive antibody therapy would mediate protection is viral neutralization. However, other mechanisms may be possible, such as antibody dependent cellular cytotoxicity and/or phagocytosis. Convalescent serum was also used in the 2013 African Ebola epidemic. A small non-randomized study in Sierra Leone revealed a significant increase in survival for those treated with convalescent whole blood relative to those who received standard treatment (5).

When used for therapy, antibody is most effective when administered shortly after the onset of symptoms. The reason for temporal variation in efficacy is not well understood but could reflect that passive antibody works by neutralizing the initial inoculum, which is likely to be much smaller than that of established disease. Another explanation is that antibody works by modifying the inflammatory response, which is also easier during the initial immune response, which may be asymptomatic (6). For example, passive antibody therapy for pneumococcal pneumonia was most effective when administered shortly after the onset of symptoms and there was no benefit if antibody administration was delayed past the third day of disease (2). Clinical outcomes after convalescent antibody therapy were better when it was administered to ill patients SARS-CoV-1 within 14 days after onset of symptoms (discussed below) (7). Our goal is to treat patients who are sick enough to warrant hospitalization but do not have severe respiratory disease and/or ARDS.

2. Experience with the use of convalescent plasma against coronavirus diseases

In the 21st century, there were two other epidemics with coronaviruses that were associated with high mortality, SARS1 in 2003 and MERS in 2012. In both outbreaks, the high mortality and absence of effective therapies led to the use of convalescent plasma. The largest study involved the treatment of 80 patients in Hong Kong with SARS (7). Consistent with historical data that earlier administration of antibody is more likely to be effective, 30 patients treated a mean of 11.7 (+/- 2.3) days after symptom onset had improved prognosis defined by discharge from hospital before day 22, whereas 47 patients who received plasma a mean of 16 days after symptom onset died before day 22 or had a late discharge. The mortality rates in the two groups were 6.3% and 21.9%, respectively (P=0.08), and those who were nasal swab PCR positive and seronegative for coronavirus at the time of therapy had improved prognosis. There is also some anecdotal information on the use of convalescent plasma in seriously ill individuals. Three patients with SARS in Taiwan were treated with 500 ml of convalescent plasma, resulting in a reduction in plasma virus titer and each survived (8). Three patients with MERS in South Korea were treated with convalescent plasma, but only two of the recipients had neutralizing antibody in their plasma (9). The latter study highlights a challenge in using convalescent plasma; some who recover may not have high titers of neutralizing antibody (10). An analysis of 99 samples of convalescent sera from patients with MERS showed 87 had neutralizing antibody with a geometric mean titer of 1:64. This suggests that antibody declines with time and/or only a few patients make high titer responses. Our study addresses this issue by screening plasma for antibody titers to SARS-CoV-2 and using high titer antibody for treatment. Although the optimal titer for treatment of SARS-CoV-2 is not established, plasma with a neutralizing titer of at least 1:64 should be administered. However, it is possible non-neutralizing antibodies may also contribute to protection as described for other viral diseases (11, 12).

A recently performed pilot study in Wuhan, China collected convalescent plasma from COVID-19 positive patients 3 weeks following the onset of illness and 4 days post-discharge and treated patients diagnosed with ‘severe COVID-19’ as defined by WHO Interim Guidance and the Guideline of Diagnosis and Treatment of COVID-19 National Health Commission of China (13). Ten patients were treated with one dose of convalescent plasma (200ml, >1:640 titer by neutralization assay) at a median of 16.5 days (11-19.3 days) post-onset of symptoms. A COVID-19 positive control cohort was retrospectively identified and matched by demographics, comorbidities, and severity of illness. There were no serious adverse reactions or safety events recorded with convalescent plasma, including no reported transfusion related reactions, transfusion-related acute lung injury, or antibody-mediated enhancement of infection. In the treatment group, there were 0 deaths, 3 discharges and 7 patients improved, whereas there were 3 deaths and 7 patients who improved in the control group ($p < 0.001$). In addition, 2 of 3 patients in the treatment on mechanical ventilation were weaned to high flow nasal canula, which was discontinued in one patient. There was a reduction in blood RNA viral load in 7 of 10 patients on day 6 post-convalescent plasma therapy as well as improvement in laboratory markers. There were also varying degrees of improvement in pulmonary lesions on chest CT after convalescent plasma therapy. In another case series from China, five severely ill patients with COVID-19, all on mechanical ventilation received convalescent plasma within 22 days of admission (14). Temperatures normalized in 4 of 5 patients within 3 days, and there was improvement in oxygenation and ARDS resolution. All survived, with 3 discharged home and 2 in stable condition. These reports suggest convalescent plasma may hold promise for ameliorating the severity of COVID-19 and deserves immediate investigation for this indication.

There are limited data on use of convalescent plasma in pregnancy. A non-randomized comparative study that evaluated use of convalescent plasma for Ebola Virus Disease reported that eight out of 84 participants were pregnant and that mortality was 25% among pregnant women and 32% among non-pregnant individuals after receiving plasma treatment (15). A case series of 4 Chinese patients, among whom one was pregnant, received convalescent plasma and had recovered from SARS-CoV-2 infection (16). We do not have robust data of using

convalescent plasma in pregnancy as most trials have excluded pregnant patients. IVIG therapy however is safe to give during pregnancy and is often used in those with inflammatory disorder and/or autoimmune conditions.

INVESTIGATIONAL PLAN:

1. Study Objectives

Primary Objective: Evaluate the efficacy of convalescent plasma from people who have recovered from COVID-19 containing antibodies to SARS-CoV-2 versus control (SS) to prevent worsening respiratory status or death in hospitalized patients with COVID-19 who are within 3 days of presentation to the hospital or within 7 days of symptom onset.

Primary Endpoint:
Primary Outcome: Status at 14 days using the WHO 11-point ordinal scale for clinical improvement which ranges from 0 (uninfected) to 10 (death). Effect size will be measured as the cumulative odds ratio comparing treatment to placebo control, estimated using a cumulative proportional odds model that adjusts for initial status (indicator for status = 5 or status = 6).

WHO ordinal scale for clinical improvement

Patient State	Score	Descriptor
Uninfected	0	Uninfected; no viral RNA detected
Ambulatory	1	Asymptomatic; viral RNA detected
	2	Symptomatic; Independent
	3	Symptomatic; assistance needed
Hospitalized: Mild disease	4	Hospitalized; no oxygen therapy
	5	Hospitalized; oxygen by mask or nasal prongs
Hospitalized: Severe disease	6	Hospitalized; oxygen by NIV or High flow
	7	Intubation & Mechanical ventilation; pO2/FIO2 >/= 150 or SpO2/FIO2 >/=200
	8	Mechanical ventilation pO2/FIO2 < 150 (SpO2/FIO2 <200) or vasopressors
	9	Mechanical ventilation pO2/FIO2 < 150 and vasopressors, dialysis or ECMO
Death	10	Dead

Secondary Objectives:
Secondary outcome; same as above at 28 days.

Exploratory objectives (To be performed at selected sites):
1. Evaluation of the anti-SARS-CoV-2 titers in convalescent (donor) and patient serum or plasma
2. Analysis of SARS-CoV-2 antibody profiles and functional assays in convalescent (donor) and patient serum or plasma
3. Analysis of the, levels of SARS-CoV-2 RNA in NP swabs
4. Analysis of SARS CoV2 sequences
5. Clinical status (WHO score, symptoms) at other visit days, mortality, and rates of discharge
6. Comparison of selected labs at various visits
7. Assessment of role of concomitant medications in clinical outcome

2. Definitions

- *Enrolled*: From time consented to participate until designated as (i) ineligible based on the inclusion/exclusion criteria or withdraws, (ii) been discontinued from the study or (iii) completed the study.
- *Randomized*: when a randomization number is assigned.
- *Screen Failures*: signed informed consent, but then determined to be ineligible or withdraws before being randomized.
- *Discontinued*: randomized, but then withdrawn by investigator or subject withdraws consent
- *Completed:* Subjects are considered completed when they are followed through to day 28, had an adverse event or death occurred prior to day 28. Patients will be asked to have day 60 and day 90 study visits as well.

3. Study Population

Inclusion Criteria:
1. Patients ≥18 years of age
2. Hospitalized with laboratory confirmed COVID-19
3. One or more of the following respiratory signs or symptoms: cough, chest pain, shortness of breath, fever, oxygen saturation ≤ 94%, abnormal CXR/CT imaging
4. Hospitalized for ≤ 72 hours OR within 7 days from first signs of illness
5. On supplemental oxygen, non-invasive ventilation or high-flow oxygen
6. Patients may be on other randomized controlled trials of pharmaceuticals for COVID -19 and patients who meet eligibility criteria will not be excluded on this basis.

Exclusion Criteria:
1. Receipt of any Covid-19 vaccine or participation in a Covid-19 vaccine study as a subject.
2. Receipt of pooled immunoglobulin in past 30 days
3. Contraindication to transfusion or history of prior reactions to transfusion blood products
4. Invasive mechanical ventilation or extracorporeal membrane oxygenation (ECMO)
5. Volume overload secondary to congestive heart failure or renal failure
6. Unlikely to survive past 72 hours from screening based on the assessment of the investigator
7. Unlikely to follow-up or clinical assessment will be hindered by patient's poor functional status

4. Subject Withdrawal

- Subjects can terminate study participation and/or withdraw consent at any time without prejudice.
- Randomized subjects who withdraw from the study will not be replaced.
- The investigator may withdraw subjects if the investigator determines that continued participation in the study would be harmful to the subject or the integrity of the study data

Table 1: Schedule of Assessments

Study period	Screen	Baseline	Transfusion	Follow up[1]						
Day	-3 to 0	0	0	1	3	7	14	28	60	90
Eligibility										

[1] Inpatient labs can be performed ±1 day from visit. If patient is discharged before day 14, the next visits will be days 14, 28, 90. Outpatient labs can be performed if able to be obtained in outpatient setting ideally ±7 days from visit. Follow-up visits after discharge can be done ±7 days from the time-point. If visits are done beyond the 7 day window, clinical status should be assessed retrospectively to the target date. If labs are done beyond the 7 day window, it must be done prior to the next follow-up visit.

Informed consent	x									
Demographic and Medical history	x									
COVID-19 symptom screen	x									
SARS-CoV-2 RT-PCR for eligibility	x[2]									
Pregnancy test	x									
ABO for plasma compatibility[3]	x									
Chest imaging (CXR or CT scan)	x[4]									
Oxygenation Level	x									
(Pro-)BNP	x[6]									
Study Drug Administration										
Randomization		x								
Drug infusion			x							
Study Procedures										
Vital signs	x	x	xxxx[5]	x	x	x				
Physical examination	x		x	x	x	x	x[6]			
Symptom screen	x	x	x	x	x	x	x	x	x	x
Concomitant medications	x	x				x	x	x	x	x
Assessment with 11-point ordinal scale		x		x	x[7]	x[7]	x[7]	x[7]	x	x
Chest imaging (CXR or CT scan)					x[6]		X[6]			
EKG	x[6]				x[6]		x[6]			
Echocardiogram and/or left heart catherization data					x[6]					
Duplex US of extremities[8]				x[8]	x[8]	x[8]	x[8]			
INR monitoring[9]				x[9]	x[9]	x[9]	x[9]			

[2] Performed within prior 7 days
[3] Prior active results may be used if available
[4] Chest imaging on admission or thereafter, before randomization. Optional repeat at day 14 or discharge, whichever comes first
[5] Vital sign testing: Immediately prior to infusion, 10-20 minutes after start of infusion, at completion of infusion and 30-60 minutes after the end of the infusion.
[6] If able to be obtained when not performed as part of standard clinical care
[7] To be done additionally at discharge day
[8] As dictated by clinical indications (suspicion of thromboembolism)
[9] As indicated clinically for patients on coumadin

Adverse event monitoring		x	x	x	x	x	x	x	x	x
Laboratory testing										
CBC with differential		x		x	x	x	x			
CMP (or BMP)		x		x	x	x	x			
LFT		x		x	x	x	x			
D-dimer[10]		x		x[10]	x[10]	x[10]	x[10]			
LDH[10]		x		x[10]	x[10]	x[10]	x[10]			
CRP[10]		x		x[10]	x[10]	x[10]	x[10]			
Ferritin[10]		x		x[10]	x[10]	x[10]	x[10]			
CPK		x[6]		x[6]	x[6]	x[6]	x[6]			
Fibrinogen		x[6]		x[6]	x[6]	x[6]	x[6]			
Procalcitonin		x[6]		x[6]	x[6]	x[6]	x[6]			
PTT		x[6]		x[6]	x[6]	x[6]	x[6]			
Troponin		x[6]		x[6]	x[6]	x[6]	x[6]			
ABG		x[6]		x[6]	x[6]	x[6]	x[6]			
(Pro-)BNP		x[6]			x[6]					
SARS-CoV-2 RT-PCR[11]						x[11]	x[1]	x[1]		x[1]
SARS-CoV-2 antibody[13,10]		x[13]	x[12,13]	x[13]		x[13]	x[1,13]	x[1,13]		x[1,13]
Lymphocyte subset[14]		x[6]		x[6]		x[6]	x[1,6]	x[1,6]		x[1,6]
Cytokine panel[15]		x[6]		x[6]		x[6]	x[1,6]	x[1,6]		x[1,6]
Blood for future testing (To be collected by selected sites)		x[6]		x[6]		x[6]	x[1,6]	x[1,6]		x[1,6]

5. Treatment

1. Subjects will be randomized in a 1:1 ratio to receive 1 unit of study product (CP) or placebo control (equivalent volume of SS) randomization will be stratified at each site by risk in Stage 1. In Stage 2, randomization will also be based on a 1:1 ratio but study arms have not yet been defined and will depend on the outcome in Stage 1.
2. Study drug: The investigational product is anti-SARS-CoV-2 convalescent plasma. Patients identified as having recovered from COVID-19 will serve as potential donors as per FDA guidelines (https://www.fda.gov/vaccines-blood-biologics/investigational-new-drug-ind-or-device-exemption-ide-process-cber/recommendations-investigational-covid-19-convalescent-plasma) and NYBC protocol (https://www.nybc.org/donate-blood/covid-19-and-blood-donation-copy/convalescent-plasma/).

---

10 If unable to obtain at all visits, can collect at baseline and then at selected visits during hospitalization
11 Repeat during discharge if able
12 Blood bank collects and ships segment from plasma bag tail for antibody testing
13 Samples to be shipped to Einstein/Montefiore Biorepository
14 ARUP labs (https://www.aruplab.com/) - 95892
15 ARUP labs (https://www.aruplab.com/) – 51394

Donors will be assessed for eligibility by the NYBC: https://www.nybc.org/donate-blood/covid-19-and-blood-donation-copy/convalescent-plasma/

3. Potential donors and samples will be screened per FDA May 1, 2020 guidelines (https://www.fda.gov/vaccines-blood-biologics/investigational-new-drug-ind-or-device-exemption-ide-process-cber/recommendations-investigational-covid-19-convalescent-plasma) and NYBC protocols (https://www.nybc.org/donate-blood/covid-19-and-blood-donation-copy/convalescent-plasma/).
4. Treatment arm will receive 1-2 units of CP antibodies to SARS-CoV-2, Stage 1.
5. Control arm will receive equivalent volume of SS, Stage 1.
6. CP will be in standard plasma unit bags, with a study specific International Society of Blood Transfusion (ISBT) label
7. Both study drugs will be blinded to minimize unblinding of the bedside nurse, research staff, and the patient.

Randomization assignments are performed for patients at baseline. Randomization is performed separately by two strata, treatment site and risk of severe disease (high versus lower) as defined below. The randomization scheme will be determined by the study *Stage* and its associated group of intervention arms (For example, in Stages 1 and 2, subjects will be randomized to 1:1 ratio). Randomization should obviate the need for additional adjustment factors but if pre-specified demographic or clinical characteristics are unbalanced with respect to treatment group, we will consider adjustment; these characteristics include but are not limited to age, sex, race, ethnicity, BMI and COVID severity at baseline. The collaborating sites will be using the interactive web response system (IWRS) developed for randomization.

6. Rationale for Dosing

One to two units of convalescent plasma (250-500mL) containing anti-SARS2-CoV-19 IgG, as determined by the NYBC protocol prior to dispensing the plasma as "convalescent plasma," will be used in the treatment group. The NYBC tests potential plasma for SARS-CoV-2 IgG prior to distributing it as "convalescent plasma." Given that the NYBC testing platform provides a qualitative SARS-CoV-2 determination, SARS-CoV-2 IgG and neutralizing titers will be determined retrospectively as per FDA guidelines (https://www.fda.gov/vaccines-blood-biologics/investigational-new-drug-ind-or-device-exemption-ide-process-cber/recommendations-investigational-covid-19-convalescent-plasma). At the discretion of the treating physician, two units (~500 mL) may be administered.

Based on previous use of CP therapy in SARS-1, 5 mL/kg of plasma at titer ≥ 1:160 was utilized (7), for a 70 Kg person. For CP therapy for COVID-19, plasma volume is estimated to be 2800mL (40 mL/kg x70 kg) with baseline anti-SARS-CoV-2 titer of 0; therefore, if protective titer was 1:25 and each unit had titer of 1:160, 500mL can achieve this ([500/(2800+500)] x 1:160>1:25). If titer is ≥1:320, 250mL can achieve this titer.

Convalescent plasma units come from single individuals with anti-SARS-CoV-2 IgG. It is not a pooled product. If a patient receives one unit it will have come from a single person. If two units are given, it will come from one person or two different people. This is not something we will know. We will know that the samples have anti-SARS-CoV-2 IgG.

The control group will receive an equivalent volume (250-500ml) of SS.

Attempts will be made to blind the appearance of the solution being administered, in order to minimize the risk of unblinding the treatment the patient, bedside nurse, and research staff.

7. Study product administration

1. Study product will be administered within 24 hours of randomization

2. Infusion rate ≤ 500 mL/hour
3. Pretreatment to minimize transfusion reactions (e.g. acetaminophen, diphenhydramine) may be given. Individual institutional guidelines/SOPs for the administration of plasma should be followed, including the use of any premeditations, such as acetaminophen and diphenhydramine.
4. If an AE develops during infusion, the infusion may be slowed or stopped as per investigator's decision in discussion with the blood bank.
    - o Most reactions to plasma are relatively minor and the infusion can generally be continued. Infusion site burning and non-allergic systemic effects can generally be managed with slowing of the infusion. Infusion can generally be continued in cases of itching or hives after pausing the transfusion, administering antihistamines, and observing the patient for worsening.
    - o Severe allergic reactions, such as bronchospasm and hypotension, require discontinuation of the infusion.
5. Post-treatment management of fluid overload (i.e. need for furosemide) as per supervising physician on a case-by-case basis.
6. Concomitant medications will be documented on the Case Report Form (CRF)
    - Prescription medications
    - Over the counter medications
    - Herbal treatments/nutritional supplements
    - Blood products
    - Any medications with established activity against SARS-CoV-2 that subject is receiving.

STATISTICAL PLAN

1. **Statistical Modeling**

   Inferences in this trial are based on a Bayesian statistical model, which estimates the posterior probability for each intervention based on the evidence that has accumulated during the trial in terms of the observed WHO ordinal outcome and assumed prior knowledge in the form of a prior distribution. The statistical model considers the variation in outcomes by site, strata, and phase of the trial.

   Details of the modeling, prior distribution assumptions, decision rules, and proposed secondary/exploratory analyses are provided in the Statistical Analysis Plan.

2. **Sample Size and Power Considerations**

   The planned total sample size for the trial is 1000 subjects, stratified by site and risk of severe disease (high versus lower), but target enrollment will be adapted based on continuous Bayesian monitoring as detailed in the SAP.

   We estimated an initial sample size for Stage 1 study design using simulations assuming a two-sided Type I error rate (alpha) of 0.05 and 80% power. We made the following additional assumptions:

    a. 30% incidence of worsening respiratory status (10% death and 20% on invasive mechanical ventilation or ECMO, respectively) and 10% of discharged alive in the control group estimated by current data from our hospital,
    b. 1.8 odds ratio (OR) of worsening respiratory status between the control group and the anti-SARS-CoV-2 convalescent plasma group, this approximately corresponds to an 13% absolute reduction in incidence of worsening respiratory status (5% death and 12% on invasive mechanical ventilation or ECMO, respectively) and 5% absolute increase of discharged alive using anti-SARS-CoV-2 convalescent plasma.
    c. Very few subjects will be randomized and fail to receive study plasma infusion or will be lost to follow-up and have missing data for the primary endpoint.

We initially estimated that a sample size of 300 patients (150 in each arm) would be sufficient to detect the specified difference in clinical status between the two arms with a power of at least 0.8; therefore, the planned total sample size of 1000 subjects is more than sufficient. Stage 1 will complete according to the monitoring rules described above, and may end with fewer patients due to futility, equivalence or superiority. Stage 2 of the study will proceed in a similar manner.

EXPLORATORY OBJECTIVES

1. Evaluation of the anti-SARS-CoV-2 titers in convalescent (donor) and patient serum or plasma.
   Evaluation of plasma IgG, IgM, and IgA titers and SARS-CoV-2 neutralizing titers will be conducted. The analysis will primarily be descriptive, comparing titers between the randomized arms using a measure of central tendency as indicated by the distribution of the data. It is also of interest to describe the distributions of anti-SARS-CoV-2 titers in each randomized arm and identify relationships between antibody isotype, neutralization, and effector cell dependent (e.g. antibody dependent cellular cytotoxicity (ADCC), antibody dependent phagocytosis (ADCP) antibodies in patient outcomes in each randomized arm and compared between arms. In addition, analyses will seek to identify minimum antibody titers and/or functional attributes of convalescent (donor) plasma and/or patient (endogenous) serum or plasma antibodies needed to achieve a clinical response. These studies may also assess the ability of SARS-CoV-2 antibodies to bind an array of SARS-CoV-2 carbohydrate, protein, or peptide determinants, and/or stimulate T cells. Similar analysis will also be applied to lymphocyte and neutrophil counts, hematological measurements (D-dimer, fibrinogen) and T and B cell subsets.

2. Analysis of SARS-CoV-2 antibody profiles and functional assays in convalescent (donor) and patient serum or plasma
   Analysis of SARS-CoV-2 antibody profiles including antibody isotype, and other serological measurements at days 0, 1, 7, 14, 28, and 90 will be done to understand how antibody response may correlate with disease trajectory. Additional serological measurements include Ab-directed functional assays including antibody-dependent cellular phagocytosis (ADCP), antibody-dependent neutrophil phagocytosis (ADNP), antibody-dependent complement deposition (ADCD), antibody dependent NK degranulation (NK Degran). Additional studies may include antibody glycosylation and antibody subsets and populations that may be explored as correlates of antibody efficacy, such as autoantibodies. These studies may provide insight into the mechanisms by which SARS-CoV-2 antibodies may mediate protection against COVID-19 and inform design of vaccines and monoclonal antibody therapies.

3. Analysis of the levels of SARS-CoV-2 RNA in NP swabs
   This exploratory analysis will be primarily descriptive. Like the analysis of anti-SARS-CoV-2 titers, the goal of this secondary aim is to describe the distribution of SARS-CoV-2 RNA between randomized arms. Therefore, we will use the same approach as for the anti-SARS-CoV-2 titers. Because the exact day that an individual becomes negative is not known, a minimum and maximum amount of positive time will be used to describe the positive duration of each individual. If the sample is adequate, we will describe the duration of positivity using a non-parametric approach for time-to-event analysis.

4. Analysis of SARS CoV2 sequences
   NP swabs that were collected from participants and stored in clinical labs will be subjected to whole virus and/or spike protein sequencing to characterize the participants' viral strains. This will make it possible to test convalescent plasma units for their reactivity with wild-type and variant strains and evaluate the contribution of participant infection with viral variants to the efficacy of convalescent plasma that contains antibodies to defined, wild-type or variant strains.

5. Clinical status (WHO score) at other visit days, mortality, and rates of discharge.
   Evaluation of the clinical status at days 3, 7, 60, and 90 will be done to learn about the rates of improvement of our cohort. Furthermore, time to clinical improvement by 2 points and 4 points in the WHO scale will be calculated, in addition to time to discharge and time to death.

6. Lymphocytes, neutrophils, and cytokines
   Will be collected on days 0, 3, 7, 14 or as obtained in care; Hematological measurements (D-dimer, fibrinogen, etc) on days 0, 3, 7, 14 or as obtained in care; and Lymphocyte cell subsets and cytokine panel on days 0, 1, 7, 14, 28, 90. Comparison of values throughout visits will be done.

7. Concomitant medications
   Medications administered to participants from enrollment through the study period will recorded and evaluated to determine if particular drugs were added, enhanced, or were detrimental to the effect of convalescent plasma or placebo.

STUDY PROCEDURES

Study Protocol by Day:

Day -3 to 0:

A. Screening (must be completed before randomization)
B. Informed consent (obtained before performing study related activities)
C. Baseline Evaluation (at screening) (much of the information will be obtained from the medical record)

1. Demographics:
   - Age, sex, race, ethnicity
2. Medical history:
   - Timing of exposure to COVID-19 source patient
   - Acute and chronic medical conditions
   - Medications, allergies
   - Any medical condition arising after consent to be recorded as AE.
3. COVID-19 symptom screen:
   - Symptoms: Fevers, cough, shortness of breath, chest pain
   - History of illness: Onset of symptoms, source of contagion
4. Vital signs
5. COVID-19 testing (RT-PCR)
   - Nasopharyngeal, oropharyngeal, tracheal aspirate, bronchoalveolar lavage. Results from laboratory tests obtained up to 7 days before enrollment may be used.
6. Baseline Basic Lab Testing and imaging
   - Blood typing (any prior active results may be used if available), (pro-)BNP (if part of standard of care)
   - Chest imaging (CXRAY or CT scan), EKG (if part of standard of care)
7. Urine or serum pregnancy test
   - For females of childbearing potential or not menopausal
   - Results from laboratory tests obtained up to 7 days before enrollment may be used for the pregnancy test
8. Determination of eligibility
   - Inclusion/exclusion criteria age
   - Consent

- Documentation of positive for COVID-19 in the prior 7 days
- Symptoms of cough, shortness of breath, chest pain, fever, or oxygen saturation ≤ 94%, not already an ICU patient
- Oxygen supplementation
- Within 7 days from first sign of illness or within 72 hours of admission

<u>Day 0:</u>

1. Randomization of eligible subject in IWRS
2. Study Plasma Administration:
    a. 1-2 units of plasma will be transfused
    b. Time at start and end of infusion will be recorded
    c. Vital signs will be measured immediately prior to infusion, 10-20 minutes after start of infusion, at completion of infusion and 30-60 minutes after the end of the infusion
    d. Blood bank will collect plasma bag tail segment for SARS-CoV-2 antibody titers
3. COVID-19 symptom screen: fevers, cough, shortness of breath
4. Assessment of clinical status (WHO ordinal scale)
5. New medical conditions, concomitant medication, AE evaluation
6. Physical examination (standard of care/per protocol)
7. CBC, CMP or BMP, CRP, LFT, D-dimer, LDH, ferritin.
8. Serological testing: anti-SARS CoV-2 titers
9. If able to be obtained when not part of standard of care: (pro-)BNP, Fibrinogen, procalcitonin, CPK, PTT, troponin, ABG, Lymphocyte subsets, and Cytokine panel.
10. Stored samples for future studies at selected sites

<u>Day 1-14 (as inpatient or for duration of hospitalization):</u>

1. Vital signs daily
2. COVID-19 symptom screen (fevers, cough, shortness of breath)
3. Assessment of clinical status (WHO ordinal scale)
4. New medical conditions, concomitant medications, AE evaluation
5. Physical examination (standard of care/per protocol)
6. CBC, CMP or BMP, LFT, CRP, LDH, ferritin, and D-Dimer
7. Serological testing: anti-SARS CoV-2 titers
8. Nasopharyngeal or throat: SARS-CoV-2 PCR
9. If able to be obtained when not part of standard of care: (pro-)BNP, Fibrinogen, procalcitonin, CPK, PTT, troponin, ABG, Lymphocyte subset, and Cytokine panel.
10. Stored samples for future studies at selected sites
11. CXR or CT (day 3, 14 or at discharge if part of standard care)
12. EKG (day 3 and and 14 if patient is still hospitalized and clinically indicated)
13. Echocardiogram parameters and right and/or left heart cardiac catheterization data, if performed for clinical care
14. Duplex ultrasound of extremities if performed for clinical care

<u>Day of Discharge:</u>

1. Define disposition (home or other)
2. Assessment of clinical status (WHO ordinal scale)

<u>Day 14 (as outpatient, phone call or in-person follow-up):</u>

1. COVID-19 symptom screen (fevers, cough, shortness of breath)
2. Assessment of clinical status (WHO ordinal scale)

3. New medical conditions, concomitant medications, AE evaluation
4. Serological testing: anti-SARS CoV-2 titers (if able to be obtained)
5. Nasopharyngeal or throat: SARS-CoV-2 PCR (if able to be obtained)
6. Lymphocyte subsets (if able to be obtained)
7. Cytokine panel (if able to be obtained)
8. Define disposition (home, hospital, status)
9. Pulmonary status (supplemental oxygen)
10. Stored samples for future studies at selected sites (if able to be obtained)

Day 28 (as outpatient, phone call or in-person follow-up):

1. COVID-19 symptom screen (fevers, cough, shortness of breath)
2. Assessment of clinical status (WHO ordinal scale)
3. New medical conditions, concomitant medications, AE evaluation
4. Serological testing: anti-SARS CoV-2 titers (if able to be obtained)
5. Nasopharyngeal or throat: SARS-CoV-2 PCR (if able to be obtained)
6. Lymphocyte subsets (if able to be obtained)
7. Cytokine panel (if able to be obtained)
8. Define disposition (home, hospital, status)
9. Pulmonary status (supplemental oxygen)
10. Stored samples for future studies at selected sites (if able to be obtained)

Day 60 (as outpatient, phone call or in-person follow-up):

1. COVID-19 symptom screen (fevers, cough, shortness of breath)
2. Assessment of clinical status (WHO ordinal scale)
3. New medical conditions, concomitant medications, AE evaluation
4. Define disposition (home, hospital, status)
5. Pulmonary status (supplemental oxygen)

Day 90 (as outpatient, phone call or in-person follow-up):

1. COVID-19 symptom screen (fevers, cough, shortness of breath)
2. Assessment of clinical status (WHO ordinal scale)
3. New medical conditions, concomitant medications, AE evaluation
4. Nasopharyngeal or throat: SARS-CoV-2 PCR (if able to be obtained)
5. Serological testing: anti-SARS CoV-2 titers (if able to be obtained)
6. Lymphocyte subsets (if able to be obtained)
7. Cytokine panel (if able to be obtained)
8. Define disposition (home, hospital, status)
9. Pulmonary status (supplemental oxygen)
10. Stored samples for future studies at selected sites (if able to be obtained)

HUMAN SUBJECTS PROTECTIONS

RISK/BENEFIT ASSESSMENT

1. **Known potential risks**

A. The protocol involves blood sample collection (see Table). Blood is collected as part of the routine care of nearly all hospitalized patients. The risk of blood collection, or phlebotomy is bruising or fainting. Some samples required for this protocol are collected as part of the usual care of the patient

and others will be specifically collected for the protocol (see Table). The latter will be collected at the same time as the former. A separate phelebotomy will only be required for post-discharge samples.

B. A theoretical risk of administration of convalescent plasma is the phenomenon of antibody-mediated enhancement of infection (ADE). ADE can occur in viral diseases, such as dengue and involves an enhancement of disease in the presence of certain antibodies. For coronaviruses, several mechanisms of ADE have been described, including the theoretical concern that antibodies to one type of coronavirus could enhance infection to another strain (17). It may be possible to predict the risk of ADE in SARS-CoV-2 experimentally, as proposed for MERS (17). Since the proposed use of convalescent plasma in the COVID-19 epidemic would rely on preparations with high titers of antibody against the same virus, SARS2-CoV-2, ADE may be unlikely. Available evidence from the use of convalescent plasma in patients with SARS1 and MERS (18) demonstrated it is safe and there were no adverse effects in a pilot study of patients with COVID-19 (13). Nevertheless, caution and vigilance will be exercised to use clinical and laboratory measures to detect evidence of enhanced infection.

C. Another theoretical risk is that antibody administration to those exposed to SARS-CoV-2 may prevent disease but modify the immune response such that those who are treated may mount attenuated immune responses. This may leave them vulnerable to subsequent re-infection. Passive antibody administration before vaccination with respiratory syncytial virus attenuated humoral but not cellular immunity (19). This will be investigated as part of this clinical trial by comparing immune responses in those who receive standard plasma and convalescent plasma. If responses differ, those with attenuated levels could be vaccinated against COVID-19 when a vaccine becomes available. Nonetheless, these concerns are modest compared to the possible benefit of reducing the risk of respiratory failure and avoiding mechanical ventilation.

D. There are also risks associated with any transfusion of plasma including transmission of transfusion transmitted viruses (e.g. HIV, HBV, HCV, etc.), allergic transfusion reactions, anaphylaxis to transfusion, febrile transfusion reaction, transfusion related acute lung injury (TRALI), transfusion associated cardiac overload (TACO), and hemolysis should ABO incompatible plasma be administered. To minimize the risks of disease transmission, all plasma will be screened for blood borne pathogens, and pathogen reduction techniques will be utilized to prepare the plasma using standardly accepted FDA guidelines that oversee plasma collection. In addition, donors will fulfill donor requirements which require a history of COVID-19 illness, a positive COVID-19 test, a two-week period of being asymptomatic post infection and a negative nasopharyngeal swab for SARS-CoV2 by PCR.

E. COVID-19 can be complicated by coagulopathy, including disseminated intravascular coagulation (DIC), which has risk of venous thromboembolism. The incidence of venous thromboembolism among COVID-19 patients may be somewhat higher than in other disease conditions (20). One study found a 31% incidence of thrombotic complications in ICU patients with COVID-19 infections (21). Transfusion with fresh frozen plasma (FFP) can increase risk of thromboembolism, but it has also been shown to protect thrombosis in some patients. A retrospective study of trauma patients who received blood products for traumatic hemorrhage had an increased risk of venous thromboembolism if they received concomitant packed red blood cell (pRBC) transfusions with fresh frozen plasma (22) may have the potential to increase risk of thromboembolism. This is unproven and this was not described in reports of CP use in China or Korea (14, 16, 23). It may also reduce the risk of COVID-19-associated risk of thrombosis if it is effective therapy. INR will be monitored in patients on coumadin.

2. Known potential benefits

The most important potential benefit of CP is that it may reduce progression to respiratory failure in patients with COVID-19, particularly in patients with early symptoms of respiratory involvement, such as cough and shortness of breath and/or pulmonary infiltrates. The benefit of CP is expected to include an improvement in symptoms, oxygenation, the need for mechanical ventilation and possibly reduced mortality. Based on historical experience with antibody administration, antibody administration is expected to be effective relatively early in disease (1). CP was safe, reduced symptoms, and improved oxygenation in a non-randomized open label study of patients with more advanced disease in Wuhan, China (13).

3. Assessment of potential risks and benefits

Given historical data showing CP was safe and possibly effective in patients with SARS1 (7, 18), and emerging data from China suggest it is safe and possibly effective in patients with severe COVID-19. Neither the safety nor the risks of CP have been established in randomized, controlled trials. The potential benefits of CP amid a humanitarian crisis warrant urgent studies of the efficacy of CP. Effective therapies are desperately needed for COVID-19. At present, none exist. Thus, in view of the lack of any proven therapy, the benefits of CP outweigh its potential risks. However, for all patients in whom CP is considered, a risk-benefit assessment will be conducted to assess individual variables. This protocol will use a randomized controlled study design to assess the efficacy of CP. The placebo control poses minimal risk, that of additional volume. We note that a recent JAMA editorial by experts noted the importance of randomized clinical trials to demonstrate efficacy of this approach and change the course of the epidemic (20).

There are limited data on the potential risks and/or benefits of CP in pregnant woman and fetus. Pregnancy can cause changes in the coagulation and fibrinolytic systems and CP may potentially benefit some individuals by providing the coagulation factors. CP was given to pregnant women in studies of patients with Ebola (Van et al. Evaluation of Convalescent Plasma for Ebola Virus Disease in Guinea. 2016. *N Engl J Med* 374:33-42) and with COVID-19 in China (Zhang et al. Treatment with convalescent plasma for critically ill patients with SARS-CoV-2 infection. 2020. *Chest* doi:10.1016/j.chest.2020.03.039.

Alternatives: The alternative to participation in this study is routine care

<u>SAFETY</u>

1. Safety measures

1. Clinical evaluations: Vital signs and symptom screen on days 0-7, 14 if still hospitalized, and symptom screens on days 28, 60, and 90.
2. Laboratory evaluations to include chest radiography (chest x-rays and/or chest CT), EKG
   Safety laboratory tests (ABO typing, urine or serum pregnancy testing, CBC, comprehensive metabolic panel, LFT, D-dimer, PTT, LDH, fibrinogen, ferritin, CRP, procalcitonin, troponin) will be performed at the local CLIA-certified clinical laboratory on days 0-7 and 14 as specified by above plan.
3. Monitoring for development of venous thromboembolism if related to plasma infusion.

2. Definitions

1. **Adverse Event (AE):** Any untoward medical occurrence in a clinical investigation subject who has received a study intervention and that does not necessarily have to have a causal relationship with the

study product. An AE can, therefore, be any unfavorable and unintended sign (including an abnormal laboratory finding, for example), symptom, or disease temporally associated with the use of the study product, whether or not considered related to the study product.

2. **Serious Adverse Event (SAE):** Any adverse event that results in any of the following outcomes:
    a. Death
    b. Life-threatening (immediate risk of death)
    c. Prolongation of existing hospitalization
    d. Persistent or significant disability or incapacity
    e. Important medical events that may not result in death, be life threatening, or require intervention or escalation of care may be considered a serious adverse event when, based upon appropriate medical judgment, they may jeopardize the subject and may require medical or surgical intervention to prevent one of the outcomes listed in this definition. Examples of such medical events include allergic bronchospasm requiring intensive treatment in an emergency room or at home, blood dyscrasias or convulsions that do not result in inpatient hospitalization.

3. **Unexpected Adverse Event (UAE):** An adverse reaction, the nature or severity of which is not consistent with the investigator's brochure.

4. **Serious and Unexpected Suspected Adverse Reaction (SUSAR):** An adverse reaction, the nature of which is not consistent with the investigator's brochure with severity as defined by SAE above.

5. **Unanticipated Problem (UP):** Unanticipated Problem that is not an Adverse Event (e.g. breaches of confidentiality, accidental destruction of study records, or unaccounted-for study drug).

6. **Protocol Deviation:** Deviation from the IRB-approved study procedures. Designated serious and non-serious

7. **Serious Protocol Deviation:** Protocol deviation that is also an SAE and/or compromises the safety, welfare or rights of subjects or others

3. Safety Reporting Requirements

1. **Reporting Interval**
   All AEs and SAEs will be documented from enrollment. All AEs and SAEs that are unexpected and directly attributable to study drug transfusion will be followed until study end point (Day 90).

   Resolution of an adverse event is defined as the return to pre-treatment status or stabilization of the condition with the expectation that it will remain chronic.

   All SAEs will be reported to the DSMB within 24hr if:
    - The event has a reasonable possibility of having been related to the study drug infusion
    - All unexpected deaths

2. **Investigator's Assessment of Adverse Events**
   The determination of seriousness, severity, and causality will be made by an on-site investigator who is qualified (licensed) to diagnose adverse event information, provide a medical evaluation of adverse events, and classify adverse events based upon medical judgment. This includes but is not limited to physicians, physician assistants, research nurses, and nurse practitioners.

For tests that are not reflective of progression of COVID-19 (e.g. liver function tests, electrolytes, neutrophil counts, bacterial cultures, BNP or pro-BNP), laboratory abnormalities will be reported as AEs if they are 5 times the upper limit of normal. Acute kidney injury will be reported if they are 1.5 times the upper limit of normal. Examples of laboratory abnormalities that pertain to progression of COVID-19 but do not need to be reported as AEs at any level unless requested by the DSMB are: lymphocyte counts and lymphocyte subset abnormalities, cytokines, D-dimer, platelet count, ferritin, fibrinogen, CRP.

Assessment of Seriousness
1. Event seriousness will be determined according to the protocol definition of an SAE
2. Assessment of Severity

Event severity will be assigned according to the grade scale scale below
1 = Mild: Transient or mild discomfort (<48 hours); no medical intervention/therapy required.
2 = Moderate: Some worsening of symptoms but no or minimal medical intervention/therapy required)
3 = Severe or Medically Significant: Escalation of medical intervention/therapy required
4 = Life-threatening: Marked escalation of medical intervention/therapy required.
5 = Death

3. **Assessment of Study Product Association**
   1. The association assessment categories that will be used for this study are:
      - **Definitely Related:** The event follows: (a) a reasonable, temporal sequence from study drug or a study procedure; and (b) cannot be explained by the known characteristics of the participant's clinical state or other therapies; and (c) evaluation of the participant's clinical state indicates to the investigator that the experience is definitely related to study procedures.
      - **Probably or Possibly Related:** The event should be assessed following the same criteria for "Definitely Related". If in the investigator's opinion at least one or more of the criteria are not present, then "probably" or "possibly" associated should be selected.
      - **Probably Not Related:** The event occurred while the participant was receiving study drug/intervention or undergoing study procedures but can reasonably be explained by the known characteristics of the participant's clinical state or other therapies.
      - **Definitely Not Related:** The event is definitely produced by the participant's clinical state or by other therapies administered to the participant.
      - **Uncertain Relationship:** The event does not meet any of the criteria previously outlined.

   2. The investigator must provide an assessment of association or relationship of AEs to the study product based on:
      - Temporal relationship of the event to the administration of study product
      - Whether an alternative etiology has been identified
      - Biological plausibility
      - Existing therapy and/or concomitant medications.

4. Safety Oversight

Monitoring Plan
1. All AEs and SAEs will be reviewed by protocol team regularly, or more often if needed.
2. A data safety monitoring board (DSMB) composed of independent experts, without conflict of interests will be established. The DSMB reports will be disseminated to all other participating sites at least annually. If there is any information that suggests the changed risk of the study or lack of benefit, the report will be distributed to other sites within 2 days. The DSMB will review the study

before initiation, and then regularly thereafter. The DSMB will review study data to evaluate the safety, enrollment, efficacy, study progress, and conduct of the study.

STUDY MODIFICATION

1. Halting Criteria for the Study: The DSMB charter and charter addendum specifies the halting criteria for this study. Termination may be suggested by the DSMB at any time.

2. Halting Criteria/Rules for Subject Infusion: Infusion of study drug will be halted if any of the following manifestations of anaphylaxis develop and will not be restarted:

- Skin or mucous membrane manifestations: hives, pruritus, flushing, swollen lips, tongue or uvula
- Respiratory compromise: dyspnea, wheezing, stridor, hypoxemia
- A decrease in systolic blood pressure to < 90 mmHg or >30% decrease from baseline or a diastolic drop of >30% from baseline.
- Tachycardia with an increase in resting heart rate to > 130 beats per minute; or bradycardia <40 that is associated with dizziness, nausea or feeling faint.
- Any other symptom or sign which in the good clinical judgment of the study clinician or supervising physician warrants halting the infusion. For example, the rapid onset of gastrointestinal symptoms, such as nausea, vomiting, diarrhea, and cramps, for instance, may be manifestations of anaphylaxis and may warrant an immediate halt prior to meeting full SAE criteria

3. Unblinding procedure

1. Unblinding of study product should be coordinated at the local site level.
2. The site PI, involved sub-investigator, and unblinded personnel are responsible for unblinding procedures. No other study team member should become unblinded.
3. Unblinding should be done only when the intervention information (convalescent plasma or saline placebo) is <u>critical</u> to the clinical management of the patient. Examples of valid reasons for unblinding:
    a. Signs and symptoms of anaphylaxis, transfusion reaction, transfusion-related acute lung injury (TRALI) or transfusion-associated circulatory overload (TACO) during or within 24 hours after the end of transfusion.
    b. Unexpected cardiac arrest or death during or within 24 hours of the end of infusion.
    c. Covid-19 vaccine administration and convalenscent plasma donation after disease recovery: all current participants will be unblinded on or after Day 29 (after receipt of treatment or placebo). All former participants (> Day 90) will be unblinded upon request.
4. Unblinding should <u>not</u> be performed for the following reasons:
    a. Clinician or investigator curiosity.
    b. Family request < Day 90.
    c. Other request not associated directly with patient care.
5. If the local site PI grants an unblinding request, s/he should notify all study PIs and the NYU DSMB in accordance with reporting guidelines. The local site PI also should submit a protocol violation reporting form to the IND holder (NYU PI) within 24 hours. Once a site's IRB approves unblinding for vaccine and plasma donation purposes, the site no longer needs to notify all study PIs and NYU DSMB, and no longer needs to submit a protocol violation form.

ETHICS

1. Ethical Standard

All sites conducting this study are committed to the integrity and quality of the clinical studies it coordinates and implements according with local institutional and regulatory requirements.

All sites participating in this research have a Federal wide Assurance (FWA) number on file with the Office for Human Research Protections (OHRP).

This assurance commits a research facility to conduct all human subjects' research in accordance with the ethical principles in The Belmont Report and any other ethical standards recognized by OHRP. Finally, per OHRP regulations, the research facility will ensure that the mandatory renewal of this assurance occurs at the times specified in the regulations.

2. Institutional Review Board

Site specific IRB will review this protocol and all protocol-related documents and procedures as required by OHRP and local requirements before subject enrollment.

3. Informed Consent Process

Each site will follow institution specific policy and process for consenting participants of legally authorized representatives for the study. A site specific protocol addendum will be provided by each site to the respective IRB. For each study Stage, a separate informed consent form will be developed for which participants will consent separately. The collaborating sites may use the electronic consent database developed for this study.

The informed consent process will be initiated before a volunteer agrees to participate in the study and should continue throughout the individual's study participation. The subject will sign the informed consent document before any procedures are undertaken for the study. A copy of the signed informed consent document will be given to the subject for their records. The consent will explain that subjects may withdraw consent at any time throughout the course of the trial. Extensive explanation and discussion of risks and possible benefits of this investigation will be provided to the subjects in understandable language. Adequate time will be provided to ensure that the subject has time to consider and discuss participation in the protocol. The consent will describe in detail the study interventions/products/procedures and risks/benefits associated with participation in the study. The rights and welfare of the subjects will be protected by emphasizing that their access to and the quality of medical care will not be adversely affected if they decline to participate in this study.

For subjects who may not have capacity due to acuity of illness or other reasons the participant's legally authorized decision maker may be contacted. If the legally authorized decision maker is willing to consent for the participant to enter the trial and is able to provide the participant's medical history, such participants will be included in the study. Subjects will be regularly assessed throughout the study with discussions with the clinical care team to determine whether or not they have regained or lost the capacity to consent. If subjects regain the capacity to consent during the study, they will be provided again with the full informed consent form to indicate that they are willing to continue in the study and allow the use of their data.

4. Subject Confidentiality

Subject confidentiality is strictly held in trust by the participating investigators, their staff, and the sponsors and their agents. No information concerning the study or the data will be released to any unauthorized third party without prior written approval of the sponsor. The results of the research study may be published, but subjects' names or identifiers will not be revealed. Records will remain confidential. To maintain confidentiality, the PI will be responsible for keeping records in a locked area and results of tests coded to prevent association with subjects' names. Data entered into computerized files will be accessible only by authorized personnel directly involved with the study and will be coded. Subjects' records will be available to the FDA, the NYBC or FDA

registered blood establishment from which plasma was obtained and their representatives, investigators at the site involved with the study, and the IRB.

5. Other Studies

Patients will also be notified of the use of de-identified data in other studies, such as pooling analyses, sub-analyses, and/or meta-analyses.

A third party/honest broker (HB) (i.e. DSMB unblinded statistitian) who has access to the data as part of their role in compiling the study's data for the DSMB and is not involved as a listed researcher on the study will access the desired records and provide researchers of other studies with de-identified data or limited data sets (where applicable and approved by the IRB and Privacy Board). As requested HB will either strip the data of all identifiers and the data set is anonymized or will assign a code to the data. The researchers of the CONTAIN trial and any other researchers who will receive the data will not have access to the information linking the code to the identifiers of the research subjects. Using the code, researchers can request, through the HB, additional information corresponding to the research subject. When the honest broker provides coded data to the research but not the method to de-code the data, then the information provided will be considered de-identified or a limited data set depending upon the data elements included in the data set.

6. Future Use of de-Identified Patient Information and Stored Specimens

During the consent process, subjects will be asked for permission to use their data and samples for future research. The confidentiality of the subject will be maintained as per HIPAA regulations. Each participant will be assigned a unique identifier at the time of consent. The identifier links the information it contains about the patient and the specimen. De-identified patient information can be used for sub-group analyses after the conclusion of the trial and/or upon permission of the DSMB if the trial is still ongoing.

No human genetic testing will be performed on the samples. Five ml of blood samples will be collected at 5 time points (See Schedule of Events). Blood samples from all trial participating sites will be sent to the Biorepository at Albert Einstein College of Medicine. Samples will be de-identified and coded upon receipt. Plasma will be separated from whole blood, aliquoted, frozen at -20°F and stored in the Biorepository and stored until use for future research.

Samples will not be shared with investigators other than investigators included in this protocol. The specimens will remain linked and at Albert Einstein College of Medicine/Montefiore Medical Center for at least 5 years. Storage beyond the study length will be an option. Any use of these specimens not specified in the current protocol will be reviewed by the IRB. There will be no plans to re-contact participants or to inform them of results.

7. Data management and monitoring

a. Source Documents

The primary source documents for this study will be the subjects' medical records. If the investigators maintain separate research records, both the medical record and the research records will be considered the source documents for the purposes of auditing the study. The investigator will retain a copy of source documents. The investigator will permit monitoring and auditing of these data, and will allow the IRB and regulatory authorities access to the original source documents. The investigator is responsible for ensuring that the data collected are complete, accurate, and recorded in a timely manner. Source documentation (the point of initial recording of information) should support the data collected and entered in to the study database and must be signed and dated by the person recording and/or reviewing the data. All data submitted should be reviewed by the site investigator and signed as required with

written or electronic signature, as appropriate. Data entered into the study database will be collected directly from subjects during study visits or will be abstracted from subjects' medical records. The subjects' medical records must record their participation in the clinical trial and what medications (with doses and frequency) or other medical interventions or treatments were administered, as well as any AEs experienced during the trial.

b. Data Management Plan

Study data will be collected at the study site(s) and entered into the study database by the study team or by transfer of data from the electronic health record. Data entry is to be completed on an ongoing basis during the study.

c. Data Capture Methods

Clinical data will be entered into a 21 CFR 11-compliant Internet Data Entry System (IDES). The data system includes password protection and internal quality checks to identify data that appear inconsistent, incomplete, or inaccurate. The collaborating sites will be using the electronic data capture (EDC) system developed by NYU for this study. For specific details regarding CRFs, please refer to the Trial's CRF instruction manual.

d. Study Record Retention

The PI is responsible for retaining all essential documents listed in the ICH GCP Guidelines. The FDA requires study records to be retained for up to 2 years after marketing approval or disapproval (21 CFR 312.62), or until at least 2 years have elapsed since the formal discontinuation of clinical development of the investigational agent for a specific indication. These records are also to be maintained in compliance with IRB/IEC, state, and federal medical records retention requirements, whichever is longest. All stored records are to be kept confidential to the extent provided by federal, state, and local law.

No study document should be destroyed. Should the investigator wish to assign the study records to another party and/or move them to another location, the site investigator must provide written notification of such intent to sponsor with the name of the person who will accept responsibility for the transferred records and/or their new location. The sponsor must be notified in writing and written permission must be received by the site prior to destruction or relocation of research records.

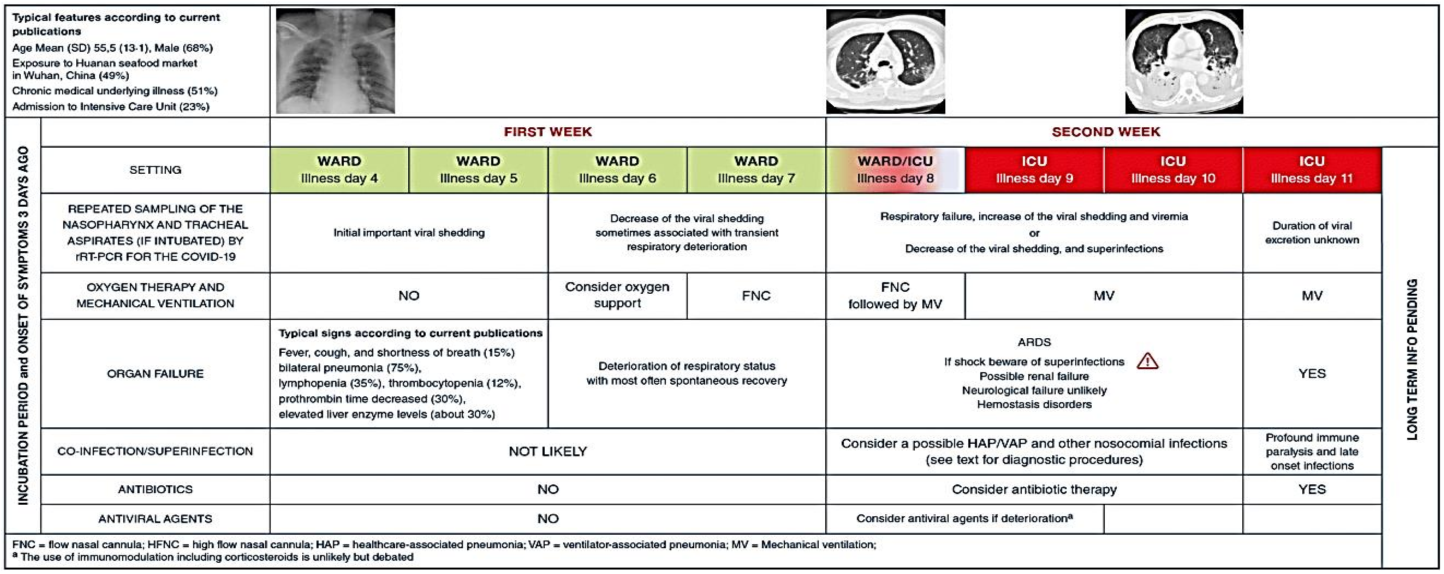

Typical features according to current publications
Age Mean (SD) 55,5 (13·1), Male (68%)
Exposure to Huanan seafood market in Wuhan, China (49%)
Chronic medical underlying illness (51%)
Admission to Intensive Care Unit (23%)

INCUBATION PERIOD and ONSET OF SYMPTOMS 3 DAYS AGO

	FIRST WEEK				SECOND WEEK			
SETTING	WARD<br>Illness day 4	WARD<br>Illness day 5	WARD<br>Illness day 6	WARD<br>Illness day 7	WARD/ICU<br>Illness day 8	ICU<br>Illness day 9	ICU<br>Illness day 10	ICU<br>Illness day 11
REPEATED SAMPLING OF THE NASOPHARYNX AND TRACHEAL ASPIRATES (IF INTUBATED) BY rRT-PCR FOR THE COVID-19	Initial important viral shedding		Decrease of the viral shedding sometimes associated with transient respiratory deterioration		Respiratory failure, increase of the viral shedding and viremia<br>or<br>Decrease of the viral shedding, and superinfections			Duration of viral excretion unknown
OXYGEN THERAPY AND MECHANICAL VENTILATION	NO		Consider oxygen support	FNC	FNC followed by MV	MV		MV
ORGAN FAILURE	**Typical signs according to current publications**<br>Fever, cough, and shortness of breath (15%)<br>bilateral pneumonia (75%),<br>lymphopenia (35%), thrombocytopenia (12%),<br>prothrombin time decreased (30%),<br>elevated liver enzyme levels (about 30%)		Deterioration of respiratory status with most often spontaneous recovery		ARDS<br>If shock beware of superinfections ⚠<br>Possible renal failure<br>Neurological failure unlikely<br>Hemostasis disorders			YES
CO-INFECTION/SUPERINFECTION	NOT LIKELY				Consider a possible HAP/VAP and other nosocomial infections (see text for diagnostic procedures)			Profound immune paralysis and late onset infections
ANTIBIOTICS	NO				Consider antibiotic therapy			YES
ANTIVIRAL AGENTS	NO				Consider antiviral agents if deterioration[a]			

LONG TERM INFO PENDING

FNC = flow nasal cannula; HFNC = high flow nasal cannula; HAP = healthcare-associated pneumonia; VAP = ventilator-associated pneumonia; MV = Mechanical ventilation;
[a] The use of immunomodulation including corticosteroids is unlikely but debated

Figure 1 *Describes what is known about the potential course of patients with COVID-19 pneumonia. The goal of this protocol is to administer convalescent plasma in the "green" area to evaluate its ability to improve the clinical respiratory status of the patient and avoid the need for respiratory support, mechanical ventilation and/or ICU admission. (from Bouadma et al Int Care Med 2020)*

REFERENCES

1. Casadevall A, Pirofski LA. 2020. The convalescent sera option for containing COVID-19. J Clin Invest doi:10.1172/JCI138003.
2. Casadevall A, Scharff MD. 1994. Serum therapy revisited: animal models of infection and development of passive antibody therapy. Antimicrob Agents Chemotherap 38:1695-1702.
3. Casadevall A, Dadachova E, Pirofski LA. 2004. Passive antibody therapy for infectious diseases. Nat Rev Microbiol 2:695-703.
4. Zhang JS, Chen JT, Liu YX, Zhang ZS, Gao H, Liu Y, Wang X, Ning Y, Liu YF, Gao Q, Xu JG, Qin C, Dong XP, Yin WD. 2005. A serological survey on neutralizing antibody titer of SARS convalescent sera. J Med Virol 77:147-50.
5. Sahr F, Ansumana R, Massaquoi TA, Idriss BR, Sesay FR, Lamin JM, Baker S, Nicol S, Conton B, Johnson W, Abiri OT, Kargbo O, Kamara P, Goba A, Russell JB, Gevao SM. 2017. Evaluation of convalescent whole blood for treating Ebola Virus Disease in Freetown, Sierra Leone. J Infect 74:302-309.
6. Casadevall A, Pirofski L. 2003. The damage-response framework of microbial pathogenesis. Nat Rev Microbiol 1:17-24.
7. Cheng Y, Wong R, Soo YO, Wong WS, Lee CK, Ng MH, Chan P, Wong KC, Leung CB, Cheng G. 2005. Use of convalescent plasma therapy in SARS patients in Hong Kong. Eur J Clin Microbiol Infect Dis 24:44-6.
8. Yeh KM, Chiueh TS, Siu LK, Lin JC, Chan PK, Peng MY, Wan HL, Chen JH, Hu BS, Perng CL, Lu JJ, Chang FY. 2005. Experience of using convalescent plasma for severe acute respiratory syndrome among healthcare workers in a Taiwan hospital. J Antimicrob Chemother 56:919-22.
9. Ko JH, Seok H, Cho SY, Ha YE, Baek JY, Kim SH, Kim YJ, Park JK, Chung CR, Kang ES, Cho D, Muller MA, Drosten C, Kang CI, Chung DR, Song JH, Peck KR. 2018. Challenges of convalescent plasma infusion therapy in Middle East respiratory coronavirus infection: a single centre experience. Antivir Ther 23:617-622.
10. Arabi YM, Hajeer AH, Luke T, Raviprakash K, Balkhy H, Johani S, Al-Dawood A, Al-Qahtani S, Al-Omari A, Al-Hameed F, Hayden FG, Fowler R, Bouchama A, Shindo N, Al-Khairy K, Carson G, Taha Y, Sadat M, Alahmadi M. 2016. Feasibility of Using Convalescent Plasma Immunotherapy for MERS-CoV Infection, Saudi Arabia. Emerg Infect Dis 22:1554-61.
11. van Erp EA, Luytjes W, Ferwerda G, van Kasteren PB. 2019. Fc-Mediated Antibody Effector Functions During Respiratory Syncytial Virus Infection and Disease. Front Immunol 10:548.
12. Gunn BM, Yu WH, Karim MM, Brannan JM, Herbert AS, Wec AZ, Halfmann PJ, Fusco ML, Schendel SL, Gangavarapu K, Krause T, Qiu X, He S, Das J, Suscovich TJ, Lai J, Chandran K, Zeitlin L, Crowe JE, Jr., Lauffenburger D, Kawaoka Y, Kobinger GP, Andersen KG, Dye JM, Saphire EO, Alter G. 2018. A Role for Fc Function in Therapeutic Monoclonal Antibody-Mediated Protection against Ebola Virus. Cell Host Microbe 24:221-233 e5.
13. Duan K, Liu B, Li C, Zhang H, Yu T, Qu J, Zhou M, Chen L, Meng S, Hu Y, Peng C, Yuan M, Huang J, Wang Z, Yu J, Gao X, Wang D, Yu X, Li L, Zhang J, Wu X, Li B, Xu Y, Chen W, Peng Y, Hu Y, Lin L, Liu X, Huang S, Zhou Z, Zhang L, Wang Y, Zhang Z, Deng K, Xia Z, Gong Q, Zhang W, Zheng X, Liu Y, Yang H, Zhou D, Yu D, Hou J, Shi Z, Chen S, Chen Z, Zhang X, Yang X. 2020. Effectiveness of convalescent plasma therapy in severe COVID-19 patients. Proceedings of the National Academy of Sciences doi:10.1073/pnas.2004168117:202004168.
14. Shen C, Wang Z, Zhao F, Yang Y, Li J, Yuan J, Wang F, Li D, Yang M, Xing L, Wei J, Xiao H, Yang Y, Qu J, Qing L, Chen L, Xu Z, Peng L, Li Y, Zheng H, Chen F, Huang K, Jiang Y, Liu D, Zhang Z, Liu Y, Liu L. 2020. Treatment of 5 Critically Ill Patients With COVID-19 With Convalescent Plasma. JAMA doi:10.1001/jama.2020.4783.

15. van GJ, Edwards T, de L, X, Semple MG, Gallian P, Baize S, Horby PW, Raoul H, Magassouba N, Antierens A, Lomas C, Faye O, Sall AA, Fransen K, Buyze J, Ravinetto R, Tiberghien P, Claeys Y, De CM, Lynen L, Bah EI, Smith PG, Delamou A, De WA, Haba N. 2016. Evaluation of Convalescent Plasma for Ebola Virus Disease in Guinea. N Engl J Med 374:33-42.
16. Zhang B, Liu S, Tan T, Huang W, Dong Y, Chen L, Chen Q, Zhang L, Zhong Q, Zhang X, Zou Y, Zhang S. 2020. Treatment with convalescent plasma for critically ill patients with SARS-CoV-2 infection. Chest doi:10.1016/j.chest.2020.03.039.
17. Wan Y, Shang J, Sun S, Tai W, Chen J, Geng Q, He L, Chen Y, Wu J, Shi Z, Zhou Y, Du L, Li F. 2020. Molecular Mechanism for Antibody-Dependent Enhancement of Coronavirus Entry. J Virol 94.
18. Mair-Jenkins J, Saavedra-Campos M, Baillie JK, Cleary P, Khaw FM, Lim WS, Makki S, Rooney KD, Nguyen-Van-Tam JS, Beck CR, Convalescent Plasma Study G. 2015. The effectiveness of convalescent plasma and hyperimmune immunoglobulin for the treatment of severe acute respiratory infections of viral etiology: a systematic review and exploratory meta-analysis. J Infect Dis 211:80-90.
19. Crowe JE, Jr., Firestone CY, Murphy BR. 2001. Passively acquired antibodies suppress humoral but not cell-mediated immunity in mice immunized with live attenuated respiratory syncytial virus vaccines. J Immunol 167:3910-8.
20. Roback JD, Guarner J. 2020. Convalescent Plasma to Treat COVID-19: Possibilities and Challenges. JAMA doi:10.1001/jama.2020.4940.

Summary of Changes in Protocol

Protocol Version	Changes Made	Justification	NYU IRB Approval Date
V 1.0_ April.13.2020	N/A	N/A	Apr.16.2021
V 2.0_ April.20.2020	1. Study Objectives: A) The control arm was changed to Lactated Ringer's (LR) Solution or Saline Solution (SS)<br>B) Primary outcome measure was modified from WHO 5-point ordinal scale for clinical improvement to the WHO 8-point ordinal scale.<br>2. Safety: Modified the cumulative incidence of serious adverse events.<br>3. Study Population: The inclusion and exclusion criteria were modified.<br>4. Treatment: A) Placebo control has been modified to equivalent volume of lactated ringers (LR) solution B) Both study drugs will be placed in opaque bags or foil for administration.<br>5. Randomization: A) Modified "Average Risk" to "Lower Risk"<br>B) Added the comorbidity of morbid obesity as "High Risk."<br>6. Study Procedures: A) Day 0: added CPK. B) Day 1-7: added two additional clinical data points.<br>7. Known Potential risks: included potential risk of venous thromboembolism.<br>8. Known Potential benefits: clarified the language in regard to potential benefits to	1. Study Objectives: The DSMB was concerned that standard plasma was an active comparator rather than a placebo. The change to LR or SS was made to eliminate exposure of the control group to the potential risks of plasma transfusion.<br>2. Safety: SAEs modified to include the outcomes due to severe COVID-19 and that from transfusion reactions.<br>3. Study Population: A) inclusion criteria to include patients on other randomized controlled trials of pharmaceuticals for COVID -19. We clarified that patients who meet eligibility criteria would not be excluded on this basis. B) exclusion criteria to include intracranial bleeding because of the theoretical concern that Lactated Ringer's solution may potentially exacerbate bleeding.<br>4. Treatment: Opaque bags or foil utilized to minimize unblinding of staff and patients.<br>5. Randomization: A) Lower risk is defined as "subjects with age <60 and without the presence of any high-risk factors." This will be patients <60 years of age AND absence of immune compromise, diabetes mellitus, cardiovascular, pulmonary comorbidities, or HIV (CD4<200)" B) Included morbid obesity as "High Risk" given the known correlation of obesity and worse outcomes in COVID-19 patients.<br>6. Study Procedures: A) CPK, a marker of muscle damage, added given its association with a more severe COVID-19 disease. B) Added venous duplex ultrasound of lower extremities and evaluation for pulmonary embolism as clinically indicated.	Apr.21.2020

	be more specific about the theoretical versus known benefits of convalescent plasma.<br>9. Monitoring Plan: modified our monitoring plan to report AE/SAE and clarified the DSMB procedures.<br>10. Halting Criteria for the Study: added additional halting criteria<br>11. Informed Consent Process: included guidelines for the consent of subjects who may be decisionally incompetent<br>Minor edits in wording throughout the protocol	7. Known Potential risks: COVID-19 can be complicated by coagulopathy, including disseminated intravascular coagulation (DIC), which has a risk of venous thromboembolism. The incidence of venous thromboembolism among COVID-19 patients may be somewhat higher than in other disease conditions.<br>8. Known Potential benefits: The most important potential benefit of CP is that it may reduce progression to respiratory failure in patients with COVID-19, particularly in patients with early symptoms of respiratory involvement, such as cough and shortness of breath, and/or pulmonary infiltrates. Neither the safety nor the risks of CP have been established in randomized, controlled trials. The potential benefits of CP amid a humanitarian crisis warrant urgent studies of the efficacy of CP.<br>9. Monitoring plan: changes made per DSMB recommendations<br>10. Halting Criteria: modified for the safety of the patient who develops transfusion-related or thromboembolic events<br>11. ICF: For subjects who may be decisionally-incompetent due to acuity of illness or other reasons, the participant's legally authorized decision-maker may be contacted and may consent on behalf of the subject	
V2.1_ April.23.2020	1. Study Procedures: removed the required collection of the vital signs for the outpatient visits at Day 14, Day 28, Day 60, and Day 90. Will keep the Physical Exam at the outpatient visit on Day 14. The Schedule of Evaluations changed to reflect this change<br>2. Safety Oversight: included the definition and rate of unacceptable toxicity.<br>Minor edits in wording throughout the protocol	1. Study Procedures: removed collection of vital signs and physical exams from outpatient visits as these visits will likely be done via phone/telehealth and will not be feasible to do the procedures.<br>2. Safety Oversight: modified with additional details to the interim monitoring plan for safety	Apr.24.2020

V 2.2_ May.4.2020	1. Study Design: high-risk and low-risk stratification updated.<br>2. Exploratory Objectives: A) included a statement specifying that certain blood work will be performed on selected sites. B) updated collection days for specific samples.<br>3. Study Objectives: Additional clarification statements made regarding primary, secondary, and exploratory objectives.<br>4. Schedule of Evaluations: updated to reflect changes in lab collections.<br>5. Randomization: included use of NYU-developed IWRS for randomization.<br>6. The rationale for Dosing: included clarifications on which donors the convalescent plasma will come from.<br>7. Data Capture Methods: inclusion of the use of NYU-created EDC system.<br>Minor edits in wording throughout the protocol.	1. Study Design: updated to be more specific about risk stratification.<br>2. Exploratory Objectives: minor corrections to labs and outpatient visits to correlate with each other.<br>3. Study Objectives: clarifications made to be consistent throughout the protocol.<br>4. Schedule of Evaluations: clarifications made to be consistent throughout the protocol<br>5. Randomization: for consistency among collaborating sites.<br>6. The rationale for Dosing: clarification that CP is not a pooled product and that donors are screened by NYBC protocol to reduce additional risk.<br>7. Data Capture Methods: for consistency among collaborating sites.	May.5.2020
V 2.3_ May.20.2020	1. Study Design and Interventions: Specified study as an adaptive study where subsequent stages of the trial will be defined after conclusions from the previous Stage.<br>2. Randomization: Randomization assignment process updated.<br>3. Primary Endpoint: Primary outcome measure updated to WHO 11-point ordinal scale for clinical improvement.<br>4. Safety: safety monitoring to be per DSMB	1. Study Design and Interventions: study built as a flexible process with the possibility of multiple interventions being investigated and with the possibility of the interventions evolving as the science evolves<br>2. Randomization: randomization based on the study Stage and its associated group of intervention arms<br>3. Primary Endpoint: DSMB recommended to update the WHO ordinal scale from 8-point to 11-point scale once it was updated by WHO as an objective tool to measure patient trajectory and resource use over the course of clinical illness.<br>4. Safety: updated per DSMB recommendations.	May.29.2020

	5. Study Population: Inclusion and Exclusion criteria updated<br>6. Treatment: A) Clarified that Stage 2 arms will be defined by Stage 1 outcome. B) Clarified that donors will be screened per updated FDA guidelines.<br>7. Statistical Plan: Updated statistical model for analysis of multiple stages.<br>8. Study Procedures: updated with minor edits.<br>9. Safety Reporting: addition of safety reporting information to DSMB and clarification on AE assessment and associations.<br>10. Informed Consent: clarification on re-assessing subjects who may have regained capacity to consent.<br>Minor edits in wording throughout the protocol.	5. Study Population: clarification of clinical status for inclusion/exclusion criteria including hospitalization length, respiratory status, survivability, and functional status to increase compliance with study endpoints.<br>6. Treatment: updated for consistency.<br>7. Statistical Plan: Inferences based on a Bayesian statistical model, which estimates the posterior probability for each intervention based on the evidence accumulated during the trial with respect to the observed WHO ordinal score outcome and prior knowledge in the form of a prior distribution. The statistical model considers the variation in outcomes by site, strata, and phase of the trial.<br>8. Study Procedures: updated for consistency.<br>9. Safety Reporting: updated for consistency.<br>10. Informed Consent: subjects who may regain the capacity to consent during the study will be provided ICF again to indicate that they are willing to continue in the study and allow the use of their data.	
V 2.4_ Aug.3.2020	1. Study Title: added acronym "CONTAIN COVID-19"<br>2. Study Product and Treatment: added New York Blood Center protocol (web link) for collection and testing of convalescent plasma<br>3. The rationale for Dosing: updated per New York Blood Center protocol<br>4. Study Procedures: added ferritin to Day 1-14 procedures<br>5. Future Use of de-Identified Patient Information and Stored Specimens:	1. Study Title: We have created the name of our clinical trial to CONTAIN COVID-19.<br>2. Study Product and Treatment: including a link for additional information<br>3. The rationale for Dosing: elaborated on NYBC protocol for testing and retrospective determination of SARS-CoV-2 IgG and neutralizing titers<br>4. Study Procedures: correction of typo (omission) to align with information already included in Table 1.<br>5. Future Use of de-Identified Patient Information and Stored Specimens: Clarification to bring in line with currently IRB-approved ICF	Aug.11.2020

	clarification that stored specimens and information will be de-identified<br>Minor edits in wording throughout the protocol		
V 2.5_ Aug.18.2020	1. Study Procedures: updates on lab collection procedures<br>2. Other Studies: Patients will be notified of the use of de-identified data in other studies, such as pooling analyses, sub-analyses, and/or meta-analyses.	1. Study Procedures: clarification made on which labs are not required if not standard of care<br>2. Other studies: clarification on de-identified data sharing and use of an honest broker	Aug.18.2020
V 2.6_ Nov.1.2020	1. Sample Size: Enrollment increased to 1000 subjects (from 300) with the expansion of the trial to sites in Miami, Houston, and New Haven.<br>2. Study Composition: updated NYU Langone Health to serve as the Clinical and Data Coordinating Center for the study under IND 20427<br>3. Exploratory Analysis: specified additional lab assays for exploratory analysis<br>4. Potential Risks: addition of risk of blood sample collection<br>5. Future Use of de-Identified Patient Information and Stored Specimens: clarification that blood samples from all participating sites will be sent to the Biorepository at Albert Einstein College of Medicine. Samples will be de-identified and coded upon receipt<br>Minor edits in wording throughout the protocol	1. Sample Size: Enrollment adapted based on continuous Bayesian monitoring as detailed in the SAP<br>2. Study Composition: clarification of the role of NYU Langone Health and that the collaborating sites will contribute recruitments as parallel but independent studies using identical protocol but operating under their site-specific IRB.<br>3. Exploratory Analysis: additional analysis to provide insights into the role of isotype, neutralizing, non-neutralizing, and effector cell dependent antibodies in patient outcomes; identify minimum required titers and functional attributes of antibodies needed to achieve a clinical response; understand how antibody response may correlate with disease trajectory; provide insight into the mechanisms by which SARS-CoV-2 antibodies may mediate protection against COVID-19 and inform design of vaccines and monoclonal antibody therapies.<br>4. Potential Risks: updated to align with ICF<br>5. Future Use of de-Identified Patient Information and Stored Specimens: consolidation of Biorepository information.	Nov.2.2020

V 3.0_ Feb.11.2021	1. Study composition: rephrased collaborating site responsibilities<br>2. Exclusion Criteria: addition of any COVID-19 vaccine or participation in a COVID-19 vaccine study as a subject<br>3. Statistical Plan: Statistical plan moved to separate statistical analysis plan<br>4. Schedule of Assessments: discharge chest imaging changed to optional<br>5. Investigator's Assessment of Adverse Events: Update to lab abnormalities and ranges that are considered AEs<br>Minor edits in wording throughout the protocol	1. Study composition: Clarification that collaborating sites will contribute recruitment and conduct the study per the FDA approved protocol with oversight by their IRBs<br>2. Exclusion Criteria: eliminate any confounding data from the effect of the vaccine<br>3. Statistical Plan: addition of separate working SAP draft<br>4. Schedule of Assessments: optional chest imaging due to feasibility of obtaining the test<br>5. Investigator's Assessment of Adverse Events: change to definition of lab abnormalities to reflect expected elevated labs	Feb.23.2021
V 3.1_ Mar.4.2021	1. Unblinding procedure: included unblinding procedures for sites and reasoning for unblinding<br>Minor edits in wording throughout the protocol	Unblinding procedure: unblinding to be coordinated at the site level by the investigator and unblinded personnel and only when unblinding is critical to the management of patient	Mar.9.2021
V 3.2_ Apr.2.2021	1. Exploratory objectives: A) reinserting Exploratory Analysis description under Statistical Plan section B) reworded throughout the protocol to match exploratory analysis description. C) adding sequencing to Exploratory Objectives	1. Exploratory objectives: A) Reinserting items erroneously deleted in prior drafts (human error) B) Reworded for consistency. C) Added to the protocol the recent data that COVID variants have emerged and may influence the efficacy of convalescent plasma	Apr.12.2021
