# Supplementary material for: Efficacy and Safety of COVID-19 Convalescent Plasma in Hospitalized Patients: A Randomized Clinical Trial
Source: JAMA Intern Med. 2021 Dec 13;182(2):1–12. doi: 10.1001/jamainternmed.2021.6850 (PMC8669605; doi:10.1001/jamainternmed.2021.6850)
Supplement: Supplement 2. — eMethods. Expanded Methods eTable 1. Summary of Posterior Predictive Checks for 10 Test Statistics Based on 10 000 Replicated Data sets eTable 2. Baseline Characteristics by Enrollment Sites and Treatment Group eTable 3. Baseline Patient Characteristics by Enrollment Quarter eTable 4. Cumulative Odds Model of WHO Scores at Day 14 by Subgroups eTable 5. Odds Ratios for Mortality at Day 14 by Subgroups eTable 6. Cumulative Odds Model of WHO Scores at Day 28 by Subgroups eTable 7. Odds Ratio for Mortality at Day 28 by Subgroups eTable 8. Baseline Patient Characteristics by Remdesivir Use at Randomization eTable 9. Baseline Patient Characteristics by Corticosteroids Use at Randomization eTable 10. CCP SARS-CoV-2 IgG and Neutralizing Titers by Quarters of Enrollment eTable 11. Baseline Characteristics and Day 14/28 Outcomes by Baseline SARS-CoV-2 IgG Status and Treatment Group eTable 12. Adverse Events and Serious Adverse Events eFigure 1. Trace Plots of Model Convergence eFigure 2. Observed Cumulative Probability With Predicted 95% Credible Interval by Treatment Group eFigure 3. Clinical Outcomes Among Patients Treated With Convalescent Plasma and Placebo 14 and 28 Days After Randomization by Enrollment Quarter eFigure 4. Cumulative OR for WHO Ordinal Scale and OR for Mortality at Day 14 in Indicated Subgroups eFigure 5. Cumulative OR for WHO Ordinal Scale and OR for Mortality at Day 14 in Indicated Subgroups eFigure 6. Posterior Probability of Mortality at Day 14 of Placebo and CCP Recipients in Indicated Subgroups Without Adjustment for any Covariates eFigure 7. Posterior Probability of Mortality at Day 28 of Placebo and CCP Recipients in Indicated Subgroups Without Adjustment for any Covariates eFigure 8. Clinical Outcome in Placebo and CCP Groups Dichotomized by Median CCP SARS-CoV-2 IgG EC50 at 14 and 28 Days After Randomization eReferences [file jamainternmed-e216850-s002.pdf]

Supplementary Online Content

Ortigoza MB, Yoon H, Goldfeld KS, et al; CONTAIN COVID-19 Consortium for the CONTAIN COVID-19 Study Group. Efficacy and safety of COVID-19 convalescent plasma in hospitalized patients: a randomized clinical trial. *JAMA Intern Med*. Published online December 13, 2021. doi:10.1001/jamainternmed.2021.6850

eMethods. Expanded Methods
eTable 1. Summary of Posterior Predictive Checks for 10 Test Statistics Based on 10,000 Replicated Datasets
eTable 2. Baseline Characteristics by Enrollment Sites and Treatment Group
eTable 3. Baseline Patient Characteristics by Enrollment Quarter
eTable 4. Cumulative Odds Model of WHO Scores at Day 14 by Subgroups
eTable 5. Odds Ratios for Mortality at Day 14 by Subgroups
eTable 6. Cumulative Odds Model of WHO Scores at Day 28 by Subgroups
eTable 7. Odds Ratio for Mortality at Day 28 by Subgroups
eTable 8. Baseline Patient Characteristics by Remdesivir Use at Randomization
eTable 9. Baseline Patient Characteristics by Corticosteroids Use at Randomization
eTable 10. CCP SARS-CoV-2 IgG and Neutralizing Titers by Quarters of Enrollment
eTable 11. Baseline Characteristics and Day 14/28 Outcomes by Baseline SARS-CoV-2 IgG Status and Treatment Group
eTable 12. Adverse Events and Serious Adverse Events
eFigure 1. Trace Plots of Model Convergence
eFigure 2. Observed Cumulative Probability With Predicted 95% Credible Interval by Treatment Group
eFigure 3. Clinical Outcomes Among Patients Treated With Convalescent Plasma and Placebo 14 and 28 Days After Randomization by Enrollment Quarter
eFigure 4. Cumulative OR for WHO Ordinal Scale and OR for Mortality at Day 14 in Indicated Subgroups
eFigure 5. Cumulative OR for WHO Ordinal Scale and OR for Mortality at Day 14 in Indicated Subgroups
eFigure 6. Posterior Probability of Mortality at Day 14 of Placebo and CCP Recipients in Indicated Subgroups Without Adjustment for any Covariates
eFigure 7. Posterior Probability of Mortality at Day 28 of Placebo and CCP Recipients in Indicated Subgroups Without Adjustment for any Covariates
eFigure 8. Clinical Outcome in Placebo and CCP Groups Dichotomized by Median CCP SARS-CoV-2 IgG EC_{50} at 14 and 28 Days After Randomization
eReferences

This supplementary material has been provided by the authors to give readers additional information about their work.

© 2021 American Medical Association. All rights reserved.

eMethods. (Expanded methods)

1. **Race and ethnicity data sources**

Collection of race and ethnicity data in this study was required by the funding agency, the National Center for Advancing Translational Sciences of the National Institutes of Health, consistent with the inclusion of women and minorities policy. Race and ethnicity data were obtained from entries in the medical record, as reported by the participants, using fixed categories. Individuals participating in the study were categorized as Asian, Hispanic, non-Hispanic Black, non-Hispanic White, other, and unknown. 'Other' included mixed race, American Indian or Alaska Native, and Native Hawaiian or other Pacific Islander. Race and ethnicity data were included to provide additional information about participants included in the study and the potential generalizability of the results.

2. **Primary and secondary outcome assessments**

The primary outcome was clinical status 14 days after randomization assessed with the 11-point WHO Ordinal Scale for Clinical Improvement[1]. For patients who remained hospitalized 14 days after randomization, primary outcome ascertainment was determined by medical record review. For patients who were discharged prior to 14 days after randomization, primary outcome ascertainment was determined through a telephone follow up. Patients who could not be reached by telephone for the primary outcome assessment at day 14 had the ordinal score carried forward from the date of discharge. The secondary outcome was clinical status 28 days after randomization assessed with the 11-point WHO Ordinal Scale for Clinical Improvement. As above, the secondary outcome ascertainment was determined by medical record review for patients hospitalized or by telephone follow up for discharged participants. Participants who

© 2021 American Medical Association. All rights reserved.

could not be reached by telephone for the secondary outcome assessment at day 28 were marked as “not done”.

3. COVID-19 Convalescent Plasma (CCP) procurement, storage and transfusion

CCP used at Montefiore Medical Center/Albert Einstein College of Medicine (Montefiore/Einstein) was obtained by an institution donor program conducted in March-April 2020 as described previously[2]. Briefly, after obtaining informed consent, blood was collected between March and April 2020 from otherwise healthy adult volunteers residing in Westchester County, Rockland County, and the Bronx, New York, who had recovered from mild to moderate COVID-19 that did not require hospitalization. Potential donors had a documented positive nasopharyngeal swab by polymerase chain reaction (PCR) for severe acute respiratory syndrome coronavirus 2 (SARS-CoV-2) during illness and had been asymptomatic for at least 14 days prior to sample collection. Serum was obtained by venipuncture (BD Vacutainer, serum), aliquoted, heat-inactivated at 56°C for 30 minutes and stored at 4°C prior to antibody screening by enzyme-linked immunosorbent assay (ELISA). Donors with SARS-CoV-2 spike protein titers > 1:1,000 on an in-house full length spike protein ELISA[3] were referred for apheresis at the New York Blood Center (NYBC). CCP units were sent from the NYBC to the Montefiore Medical Center (MMC) blood bank on the Moses Campus for storage. These units were used at MMC from May 4, 2020, when the trial launched at Einstein/Montefiore until January 15, 2021, when the trial switched to NYBC high titer plasma having a signal-to-cutoff ratio ≥12 on the Ortho V platform[4].

© 2021 American Medical Association. All rights reserved.

For all other CONTAIN COVID-19 sites, CCP was provided by the NYBC and was collected from donors with a positive anti-SARS-CoV-2 antibody test measured by the New York SARS-CoV-2 Microsphere Immunoassay at the NYBC[5]. Beginning in January 2021, all sites used CCP that was qualified by the NYBC as 'high titer' using the Ortho-Clinical Diagnostics VITROS Anti-SARS-CoV-2 IgG platform with signal-to-cutoff ratio ≥12 as per FDA guidance[4].

Plasma recipients were transfused with 1 unit (approximately 200-250 mL) of ABO-type matched CCP over 2–3 hours and monitored before, during, and after infusion for signs of transfusion-related reactions per standard transfusion protocol. Individual institutional guidelines or standard operating procedure (SOP) for the administration of CCP were followed, including the use of any pre-medications, such as acetaminophen and diphenhydramine. Post-treatment management of fluid overload was provided on a case-by-case basis.

4. **SARS-CoV-2 spike protein IgG titers of convalescent (donor) plasma and patient plasma before transfusion of study product**

CCP bag segments (CCP segments, remnant tubing-containing plasma) were removed locally at the blood bank of each site during thawing of CCP bag and kept at 4°C. CCP segments were used to avoid CCP bag compromise which would pose a risk of contamination to participants. These segments were shipped from local trial sites to the Montefiore/Einstein Biorepository where they were aliquoted, frozen, and thawed for retrospective determination of spike protein IgG and pseudovirus neutralization. The SARS-CoV-2 spike ectodomain IgG titer in 406 administered CCP units was determined retrospectively by the Montefiore/Einstein in house ELISA[3,6,7].

© 2021 American Medical Association. All rights reserved.

Participant SARS-CoV-2 spike protein-binding IgG titers were determined on plasma samples obtained prior to transfusion of CCP or placebo using the Montefiore/Einstein spike ectodomain protein ELISA as described previously[2]. Titers are reported as half-maximal effective concentration (EC_{50}) values derived by a curve fitting model as follows: First, each experimental repeat was processed independently by subtracting the corresponding background noise and normalizing the Optical Densities (OD) relative to a positive control. For each experimental repeat, background noise was inferred by considering the lowest OD readout. Following this, we jointly processed denoised and normalized ODs to fit a single sigmoidal curve (using least-squares minimization) and estimate the corresponding EC_{50}/IC_{50}:

$$y = y_{min} + \ (y_{max} - y_{min})/\left[1 + 10^{\left((log_{10}EC_{50} - x)\times Hill\right)}\right]$$

Where y corresponds to the OD (denoised and normalized); y_{min} and y_{max} are the minimum and maximum ODs, respectively; EC_{50} is the titer that gives half-maximum absorbance, y_{max}; Hill describes the slope of the curve, and x is the $\log_{10}$ (1/dilution). Curve fitting of ELISA readouts was performed by constraining y_{min} to 0.

5. Recombinant vesicular stomatitis virus (VSV)-SARS-CoV-2 S neutralization assay

The neutralization assay was performed as previously described[8]. Results are reported as EC_{50} values as done with SARS-CoV-2 IgG above with the exception that in the case of neutralization readouts, we constrained y_{min} and y_{max} to be between 0-100 and 0-150 respectively.

© 2021 American Medical Association. All rights reserved.

6. Statistical analysis of CCP and participant SARS-CoV-2 IgG and CCP neutralization titers

All randomized participants in the placebo group, and all CCP recipients who had CCP IgG titer measured were included in the analysis of SARS-CoV-2 IgG titers in accordance with their randomization arm, except for participants who fully withdrew informed consent or those whose blood samples could not be obtained for SARS-CoV-2 IgG testing. We evaluated the association between treatment arm and the WHO ordinal outcome using a cumulative odds model and mortality using a logistic regression model at days 14 and 28. To investigate the dose response, we analyzed the log transformed IgG titer, neutralization data, and segmentation EC_{50} adjusting for pre-specified covariates, age, sex, WHO score at randomization, and symptom duration. For neutralization data, we dichotomized patients in the CCP group using different cut off points (1:80/1:160/1:320). We also performed subgroup analysis within the patient groups that were not receiving remdesivir or corticosteroids, tested the interactions between the high/low neutralization group with remdesivir or corticosteroids use, and enrollment quarter respectively. For segment EC_{50}, CCP group was dichotomized at the median IgG EC_{50} value and association between the three treatment arms (placebo, intervention group with CCP EC_{50} below the median (low EC_{50}), and intervention group with CCP EC_{50} above the median (high EC_{50})) with the outcome was evaluated.

In addition, we evaluated the association between baseline SARS-CoV-2 IgG EC_{50} and outcomes. Participants were categorized as seronegative or seropositive using a baseline IgG EC_{50} cutoff of 1:100. IgG EC_{50}s were log-transformed for the analysis. To investigate the effect of baseline antibody on treatment benefit, we tested the interaction between baseline antibody status with the CCP treatment.

© 2021 American Medical Association. All rights reserved.

7. Assessing the proportional odds assumption

To validate the goodness of fit for our Bayesian models, we drew replications of data from the joint posterior predictive distribution and compared these samples to observed data. We used the cumulative proportions of participants in each of the WHO score categories to measure the discrepancy between the original dataset and outcomes reconstructed from our Bayesian model[9]. From the primary cumulative odds Bayesian model that we fitted, we sampled 10,000 simulations from the posterior density of the set of parameters. Then we generated one hypothetical replicated dataset of outcomes using each simulated set of parameters and the identical explanatory variables from original dataset. Finally, we estimated the Bayesian *P* value by calculating the proportion of these 10,000 replicated datasets for which the test quantities equal or exceeds its realized value.

© 2021 American Medical Association. All rights reserved.

eTables and eFigures

eTable 1: Summary of posterior predictive checks for ten test statistics based on 10,000 replicated datasets.

**Treatment**	**CCP**			**Control**		
**Test quantity: % subjects**	T(Data^{ori})	95% CrI for T(Data^{rep})	Bayesian P value	T(Data^{ori})	95% CrI for T(Data^{rep})	Bayesian *P* value
WHO >= 10	0.08	[0.04, 0.11]	0.32	0.08	[0.04, 0.12]	0.30
WHO >= 9	0.11	[0.07, 0.17]	0.48	0.14	[0.07, 0.19]	0.21
WHO >= 8	0.15	[0.10, 0.21]	0.39	0.17	[0.10, 0.23]	0.27
WHO >= 7	0.16	[0.10, 0.23]	0.38	0.19	[0.11,0.25]	0.30
WHO >= 6	0.23	[0.14, 0.29]	0.26	0.23	[0.15,0.32]	0.40
WHO >= 5	0.29	[0.20, 0.37]	0.35	0.31	[0.21,0.39]	0.35
WHO >= 4	0.31	[0.22, 0.40]	0.38	0.34	[0.23,0.42]	0.35
WHO >= 3	0.43	[0.32, 0.53]	0.42	0.46	[0.34,0.56]	0.39
WHO >= 2	0.76	[0.64, 0.83]	0.33	0.77	[0.67,0.85]	0.42
WHO >= 1	0.92	[0.84, 0.95]	0.29	0.93	[0.86,0.96]	0.29

Abbreviations: CCP, COVID-19 Convalescent Plasma; CrI, credible interval; WHO, World Health Organization.

© 2021 American Medical Association. All rights reserved.

eTable 2: Baseline Characteristics by Enrollment Sites and Treatment Group

	NYU	Einstein	Yale	Miami	UT-Houston	UT-Tyler	JHU	Wisc
n	380	160	47	106	146	100	1	1
Enrollment quarters (%)								
2020 Q2	140 (36.8)	30 (18.8)	0 (0.0)	0 (0.0)	0 (0.0)	0 (0.0)	0 (0.0)	0 (0.0)
2020 Q3	12 (3.2)	20 (12.5)	4 (8.5)	9 (8.5)	17 (11.6)	51 (51.0)	0 (0.0)	0 (0.0)
2020 Q4	130 (34.2)	58 (36.2)	37 (78.7)	66 (62.3)	76 (52.1)	40 (40.0)	0 (0.0)	0 (0.0)
2021 Q5	98 (25.8)	52 (32.5)	6 (12.8)	31 (29.2)	53 (36.3)	9 (9.0)	1 (100.0)	1 (100.0)
Age (mean (SD))	65.5 (15.1)	62.3 (15.4)	64.6 (13.7)	61.6 (14.8)	54.6 (13.6)	61.8 (14.6)	83.0 (NA)	47.0 (NA)
Age (categorical) (%)								
<45 years	34 (8.9)	26 (16.2)	4 (8.5)	15 (14.2)	34 (23.3)	13 (13.0)	0 (0.0)	0 (0.0)
45-64 years	138 (36.3)	59 (36.9)	19 (40.4)	42 (39.6)	83 (56.8)	34 (34.0)	0 (0.0)	1 (100.0)
65-80 years	140 (36.8)	53 (33.1)	20 (42.6)	40 (37.7)	23 (15.8)	45 (45.0)	0 (0.0)	0 (0.0)
>80 years	68 (17.9)	22 (13.8)	4 (8.5)	9 (8.5)	6 (4.1)	8 (8.0)	1 (100.0)	0 (0.0)
Sex, Female (%)	143 (37.6)	74 (46.2)	17 (36.2)	37 (34.9)	70 (47.9)	43 (43.0)	1 (100.0)	0 (0.0)
Blood type (%)								
O	179 (47.1)	89 (55.6)	26 (55.3)	59 (55.7)	93 (63.7)	42 (42.0)	1 (100.0)	0 (0.0)
A	114 (30.0)	39 (24.4)	16 (34.0)	28 (26.4)	36 (24.7)	41 (41.0)	0 (0.0)	0 (0.0)
B	66 (17.4)	23 (14.4)	2 (4.3)	19 (17.9)	11 (7.5)	13 (13.0)	0 (0.0)	1 (100.0)
AB	20 (5.3)	9 (5.6)	3 (6.4)	0 (0.0)	5 (3.4)	4 (4.0)	0 (0.0)	0 (0.0)
Unknown	1 (0.3)	0 (0.0)	0 (0.0)	0 (0.0)	1 (0.7)	0 (0.0)	0 (0.0)	0 (0.0)
Time between symptom onset and randomization (%)								
<4 days	46 (12.1)	34 (21.2)	3 (6.4)	27 (25.5)	19 (13.0)	23 (23.0)	0 (0.0)	1 (100.0)
4-7 days	154 (40.5)	84 (52.5)	18 (38.3)	45 (42.5)	74 (50.7)	60 (60.0)	1 (100.0)	0 (0.0)
8-11 days	125 (32.9)	31 (19.4)	18 (38.3)	25 (23.6)	34 (23.3)	14 (14.0)	0 (0.0)	0 (0.0)
12-15 days	39 (10.3)	6 (3.8)	2 (4.3)	7 (6.6)	13 (8.9)	3 (3.0)	0 (0.0)	0 (0.0)
>15 days	16 (4.2)	5 (3.1)	5 (10.6)	2 (1.9)	6 (4.1)	0 (0.0)	0 (0.0)	0 (0.0)
NA	0 (0.0)	0 (0.0)	1 (2.1)	0 (0.0)	0 (0.0)	0 (0.0)	0 (0.0)	0 (0.0)
WHO score at randomization, 5 (%)	279 (73.4)	135 (84.4)	26 (55.3)	69 (65.1)	84 (57.5)	78 (78.0)	1 (100.0)	1 (100.0)

© 2021 American Medical Association. All rights reserved.

High Risk (%)	273 (71.8)	148 (92.5)	42 (89.4)	99 (93.4)	119 (81.5)	94 (94.0)	1 (100.0)	1 (100.0)
Corticosteroids (%)	236 (62.1)	117 (73.1)	46 (97.9)	102 (96.2)	132 (90.4)	87 (87.0)	1 (100.0)	0 (0.0)
Remdesivir (%)	126 (33.2)	111 (69.4)	45 (95.7)	95 (89.6)	114 (78.1)	45 (45.0)	1 (100.0)	0 (0.0)
Diabetes (%)	115 (30.3)	58 (36.2)	17 (36.2)	34 (32.1)	65 (44.5)	42 (42.0)	0 (0.0)	1 (100.0)
Pulmonary (%)	35 (9.2)	14 (8.8)	7 (14.9)	14 (13.2)	4 (2.7)	22 (22.0)	0 (0.0)	1 (100.0)
Cardiovascular (%)	194 (51.1)	59 (36.9)	22 (46.8)	33 (31.1)	57 (39.0)	38 (38.0)	0 (0.0)	1 (100.0)

Abbreviations: JHU, Johns Hopkins University; NA, not available; NYU, New York University; Q, quarter; SD, standard deviation; UT, University of Texas; WHO, World Health Organization; Wisc, Wisconsin.

© 2021 American Medical Association. All rights reserved.

eTable 3: Baseline Patient Characteristics by Enrollment Quarter

	Enrollment quarters			
	**2020Q2**	**2020Q3**	**2020Q4**	**2021Q5**
n	170	113	407	251
Sites (merged) (%)				
New York University	140 (82.4)	12 (10.6)	130 (31.9)	98 (39.0)
Einstein/Montefiore	30 (17.6)	20 (17.7)	58 (14.3)	52 (20.7)
Yale University	0 (0.0)	4 (3.5)	37 (9.1)	6 (2.4)
University of Miami	0 (0.0)	9 (8.0)	66 (16.2)	31 (12.4)
UT-Houston	0 (0.0)	17 (15.0)	76 (18.7)	53 (21.1)
UT-Tyler	0 (0.0)	51 (45.1)	40 (9.8)	9 (3.6)
Johns Hopkins University	0 (0.0)	0 (0.0)	0 (0.0)	1 (0.4)
Medical College of Wisconsin & Froedtert Hospital	0 (0.0)	0 (0.0)	0 (0.0)	1 (0.4)
Age (mean (SD))	69.7 (14.6)	59.0 (15.2)	61.3 (15.3)	60.7 (14.0)
Age (categorical) (%)				
<45 years	10 (5.9)	20 (17.7)	63 (15.5)	33 (13.1)
45-64 years	54 (31.8)	47 (41.6)	158 (38.8)	117 (46.6)
65-80 years	57 (33.5)	39 (34.5)	145 (35.6)	80 (31.9)
>80 years	49 (28.8)	7 (6.2)	41 (10.1)	21 (8.4)
Sex, Female (%)	68 (40.0)	45 (39.8)	156 (38.3)	116 (46.2)
Blood type (%)				
O	84 (49.4)	54 (47.8)	225 (55.3)	126 (50.2)
A	52 (30.6)	43 (38.1)	111 (27.3)	68 (27.1)
B	27 (15.9)	15 (13.3)	48 (11.8)	45 (17.9)
AB	7 (4.1)	1 (0.9)	21 (5.2)	12 (4.8)
Unknown	0 (0.0)	0 (0.0)	2 (0.5)	0 (0.0)
Time between symptom onset and randomization (%)				
<4 days	30 (17.6)	27 (23.9)	65 (16.0)	31 (12.4)
4-7 days	44 (25.9)	56 (49.6)	186 (45.7)	150 (59.8)
8-11 days	45 (26.5)	22 (19.5)	122 (30.0)	58 (23.1)
12-15 days	34 (20.0)	6 (5.3)	22 (5.4)	8 (3.2)
>15 days	17 (10.0)	2 (1.8)	11 (2.7)	4 (1.6)
NA	0 (0.0)	0 (0.0)	1 (0.2)	0 (0.0)
WHO score at randomization, 5 (%)	135 (79.4)	70 (61.9)	288 (70.8)	180 (71.7)
High Risk (%)	106 (62.4)	101 (89.4)	348 (85.5)	222 (88.4)
Corticosteroids (%)	40 (23.5)	96 (85.0)	366 (89.9)	219 (87.3)
Remdesivir (%)	2 (1.2)	53 (46.9)	285 (70.0)	197 (78.5)
Diabetes (%)	60 (35.3)	46 (40.7)	128 (31.4)	98 (39.0)
Pulmonary (%)	22 (12.9)	13 (11.5)	37 (9.1)	25 (10.0)
Cardiovascular (%)	98 (57.6)	47 (41.6)	159 (39.1)	100 (39.8)

Note: Corticosteroids include IV and PO corticosteroids at randomization.
Abbreviations: NA, not available; Q, quarter; SD, standard deviation; UT, University of Texas Health Science Center; WHO, World Health Organization.

© 2021 American Medical Association. All rights reserved.

eTable 4: Cumulative Odds Model of WHO Scores at Day 14 by Subgroups

	Posterior distribution of OR				
**Subgroup**	**2.5%**	**Median**	**97.5%**	**P(OR<1)**	**P(OR<0.8)**
Age < 65 (n=496)	0.740	0.995	1.339	0.513	0.074
Age ≥ 65 (n=430)	0.657	0.891	1.206	0.773	0.247
Symptom duration, 0-3 days (n=149)	0.583	0.919	1.461	0.638	0.279
Symptom duration, 4-7 days (n=432)	0.785	1.072	1.464	0.332	0.033
Symptom duration, > 7 days (n=345)	0.645	0.907	1.279	0.720	0.233
Baseline WHO score, 5 (n=660)	0.711	0.926	1.213	0.717	0.134
Baseline WHO score, 6 (n=266)	0.665	0.966	1.396	0.568	0.161
Corticosteroids = No & Remdesivir = No (n=181)	0.475	0.736	1.152	0.915	0.642
Corticosteroids = No & Remdesivir = Yes (n=37)	0.651	1.216	2.270	0.270	0.095
Corticosteroids = Yes & Remdesivir = No (n=217)	0.471	0.709	1.059	0.952	0.721
Corticosteroids = Yes & Remdesivir = Yes (n=491)	0.891	1.194	1.602	0.120	0.003
Q2 (Apr-June 2020) (n=168)	0.519	0.811	1.265	0.823	0.476
Q3 (July-Sept 2020) (n=112)	0.542	0.897	1.489	0.664	0.325
Q4 (Oct-Dec 2020) (n=401)	0.717	0.978	1.361	0.554	0.109
Q5 (Jan-Mar 2021) (n=245)	0.747	1.092	1.607	0.317	0.054
Baseline SARS-CoV-2 IgG, Negative (n=239)	0.775	1.147	1.693	0.249	0.035
Baseline SARS-CoV-2 IgG, Positive (n=482)	0.716	0.960	1.287	0.601	0.109
Overall (n=926)	0.748	0.936	1.175	0.721	0.081

Note: 2020 Q2 had NY sites only; the model for the group (Steroids = No & Remdesivir = Yes) controls for age and sex.
Abbreviations: OR, odds, ratio; Q, quarter; SARS-CoV-2, severe acute respiratory syndrome coronavirus 2; WHO, World Health Organization.

© 2021 American Medical Association. All rights reserved.

eTable 5: Odds Ratios for Mortality at Day 14 by Subgroups

	Posterior distribution of OR				
**Subgroup**	**2.5%**	**Median**	**97.5%**	**P(OR<1)**	**P(OR<0.8)**
Age < 65 (n=496)	0.594	1.096	1.995	0.384	0.161
Age ≥ 65 (n=430)	0.563	0.907	1.462	0.655	0.300
Symptom duration, 0-3 days (n=149)	0.565	1.053	1.915	0.433	0.196
Symptom duration, 4-7 days (n=432)	0.644	1.117	1.952	0.345	0.123
Symptom duration, > 7 days (n=345)	0.457	0.810	1.429	0.757	0.483
Baseline WHO score, 5 (n=660)	0.508	0.891	1.543	0.659	0.351
Baseline WHO score, 6 (n=266)	0.636	1.068	1.776	0.404	0.134
Corticosteroids = No & Remdesivir = No (n=181)	0.514	0.936	1.756	0.580	0.306
Corticosteroids = No & Remdesivir = Yes (n=37)	0.529	1.035	2.042	0.461	0.228
Corticosteroids = Yes & Remdesivir = sNo (n=217)	0.539	1.003	1.876	0.496	0.242
Corticosteroids = Yes & Remdesivir = Yes (n=491)	0.675	1.195	2.078	0.265	0.079
Q2 (Apr-June 2020) (n=168)	0.453	0.827	1.503	0.727	0.458
Q3 (July-Sept 2020) (n=112)	0.557	1.029	1.967	0.461	0.211
Q4 (Oct-Dec 2020) (n=401)	0.542	0.975	1.746	0.536	0.251
Q5 (Jan-Mar 2021) (n=245)	0.557	1.024	1.824	0.467	0.216
Baseline SARS-CoV-2 IgG, Negative (n=239)	0.572	1.063	2.034	0.426	0.187
Baseline SARS-CoV-2 IgG, Positive (n=482)	0.786	1.366	2.388	0.132	0.029
Overall (n=926)	0.639	0.986	1.533	0.528	0.171

Note: 2020 Q2 had NY sites only; the model for the group (Steroids = No & Remdesivir = Yes) controls for age and sex.
Abbreviations: OR, odds, ratio; Q, quarter; SARS-CoV-2, severe acute respiratory syndrome coronavirus 2; WHO, World Health Organization.

© 2021 American Medical Association. All rights reserved.

eTable 6: Cumulative Odds Model of WHO Scores at Day 28 by Subgroups

	Posterior distribution of OR				
**Subgroup**	**2.5%**	**Median**	**97.5%**	**P(OR<1)**	**P(OR<0.8)**
Age < 65 (n=496)	0.761	1.026	1.383	0.431	0.048
Age ≥ 65 (n=430)	0.618	0.839	1.141	0.868	0.378
Symptom duration, 0-3 days (n=149)	0.541	0.861	1.387	0.729	0.382
Symptom duration, 4-7 days (n=432)	0.758	1.030	1.398	0.430	0.055
Symptom duration, > 7 days (n=345)	0.649	0.914	1.283	0.694	0.227
Baseline WHO score, 5 (n=660)	0.685	0.885	1.145	0.824	0.227
Baseline WHO score, 6 (n=266)	0.684	1.000	1.467	0.499	0.121
Corticosteroids = No & Remdesivir = No (n=181)	0.409	0.651	1.020	0.968	0.815
Corticosteroids = No & Remdesivir = Yes (n=37)	0.662	1.248	2.299	0.247	0.081
Corticosteroids = Yes & Remdesivir = No (n=217)	0.561	0.841	1.268	0.789	0.407
Corticosteroids = Yes & Remdesivir = Yes (n=491)	0.851	1.143	1.541	0.192	0.009
Q2 (Apr-June 2020) (n=168)	0.464	0.722	1.127	0.926	0.671
Q3 (July-Sept 2020) (n=112)	0.499	0.830	1.386	0.765	0.440
Q4 (Oct-Dec 2020) (n=401)	0.720	0.989	1.368	0.524	0.095
Q5 (Jan-Mar 2021) (n=245)	0.814	1.183	1.744	0.188	0.020
Baseline SARS-CoV-2 IgG, Negative (n=238)	0.810	1.200	1.791	0.184	0.021
Baseline SARS-CoV-2 IgG, Positive (n=482)	0.699	0.931	1.240	0.680	0.155
Overall (n=926)	0.741	0.924	1.156	0.759	0.100

Note: 2020 Q2 had NY sites only; the model for the group (Steroids = No & Remdesivir = Yes) controls for age and sex.
Abbreviations: OR, odds, ratio; Q, quarter; SARS-CoV-2, severe acute respiratory syndrome coronavirus 2; WHO, World Health Organization.

© 2021 American Medical Association. All rights reserved.

eTable 7: Odds Ratio for Mortality at Day 28 by Subgroups

	Posterior distribution of OR				
**Subgroup**	**2.5%**	**Median**	**97.5%**	**P(OR<1)**	**P(OR<0.8)**
Age < 65 (n=496)	0.570	0.959	1.633	0.559	0.249
Age ≥ 65 (n=430)	0.532	0.813	1.234	0.832	0.472
Symptom duration, 0-3 days (n=149)	0.537	0.962	1.709	0.552	0.268
Symptom duration, 4-7 days (n=432)	0.540	0.884	1.425	0.695	0.345
Symptom duration, > 7 days (n=345)	0.510	0.872	1.495	0.690	0.373
Baseline WHO score, 5 (n=660)	0.515	0.833	1.362	0.767	0.432
Baseline WHO score, 6 (n=266)	0.598	0.946	1.487	0.596	0.228
Corticosteroids = No & Remdesivir = No (n=181)	0.436	0.774	1.356	0.813	0.545
Corticosteroids = No & Remdesivir = Yes (n=37)	0.519	1.004	1.961	0.496	0.256
Corticosteroids = Yes & Remdesivir = No (n=217)	0.489	0.864	1.525	0.690	0.398
Corticosteroids = Yes & Remdesivir = Yes (n=491)	0.676	1.131	1.904	0.320	0.096
Q2 (Apr-June 2020) (n=168)	0.481	0.832	1.438	0.739	0.448
Q3 (July-Sept 2020) (n=112)	0.493	0.925	1.751	0.593	0.329
Q4 (Oct-Dec 2020) (n=401)	0.592	0.981	1.642	0.526	0.227
Q5 (Jan-Mar 2021) (n=245)	0.469	0.823	1.445	0.744	0.461
Baseline SARS-CoV-2 IgG, Negative (n=238)	0.534	0.938	1.647	0.588	0.292
Baseline SARS-CoV-2 IgG, Positive (n=482)	0.713	1.190	1.961	0.254	0.069
Overall (n=926)	0.595	0.863	1.245	0.782	0.342

Note: 2020 Q2 had NY sites only; the model for the group (Steroids = No & Remdesivir = Yes) controls for age and sex.
Abbreviations: OR, odds, ratio; Q, quarter; SARS-CoV-2, severe acute respiratory syndrome coronavirus 2; WHO, World Health Organization.

© 2021 American Medical Association. All rights reserved.

eTable 8: Baseline Patient Characteristics by Remdesivir use at Randomization

	Remdesivir at Randomization		
	**No**	**Yes**	***P***
n	404	537	
Enrollment quarters (%)			<0.001
2020 Q2	168 (41.6)	2 (0.4)	
2020 Q3	60 (14.9)	53 (9.9)	
2020 Q4	122 (30.2)	285 (53.1)	
2021 Q5	54 (13.4)	197 (36.7)	
Age (mean (SD))	65.4 (15.2)	60.1 (14.8)	<0.001
Age (categorical) (%)			<0.001
<45 years	43 (10.6)	83 (15.5)	
45-64 years	139 (34.4)	237 (44.1)	
65-80 years	148 (36.6)	173 (32.2)	
>80 years	74 (18.3)	44 (8.2)	
Sex, Female (%)	170 (42.1)	215 (40.0)	0.573
Blood type (%)			0.520
O	202 (50.0)	287 (53.4)	
A	124 (30.7)	150 (27.9)	
B	63 (15.6)	72 (13.4)	
AB	14 (3.5)	27 (5.0)	
Unknown	1 (0.2)	1 (0.2)	
Time between symptom onset and randomization (%)			<0.001
<4 days	81 (20.0)	72 (13.4)	
4-7 days	153 (37.9)	283 (52.8)	
8-11 days	103 (25.5)	144 (26.9)	
12-15 days	42 (10.4)	28 (5.2)	
>15 days	25 (6.2)	9 (1.7)	
WHO score at randomization, 5 (%)	322 (79.7)	351 (65.4)	<0.001
High Risk (%)	313 (77.5)	464 (86.4)	<0.001
Diabetes (%)	149 (36.9)	183 (34.1)	0.411
Pulmonary (%)	47 (11.6)	50 (9.3)	0.293
Cardiovascular (%)	205 (50.7)	199 (37.1)	<0.001

Abbreviations: Q, quarter; SD, standard deviation; WHO, World Health Organization.

© 2021 American Medical Association. All rights reserved.

eTable 9: Baseline Patient Characteristics by Corticosteroids use at Randomization

	Corticosteroids at Randomization		
	No	Yes	*P*
n	220	721	
Enrollment quarters (%)			<0.001
2020 Q2	130 (59.1)	40 (5.5)	
2020 Q3	17 (7.7)	96 (13.3)	
2020 Q4	41 (18.6)	366 (50.8)	
2021 Q5	32 (14.5)	219 (30.4)	
Age (mean (SD))	67.8 (15.3)	60.7 (14.8)	<0.001
Age (categorical) (%)			<0.001
<45 years	19 (8.6)	107 (14.8)	
45-64 years	73 (33.2)	303 (42.0)	
65-80 years	77 (35.0)	244 (33.8)	
>80 years	51 (23.2)	67 (9.3)	
Sex, Female (%)	87 (39.5)	298 (41.3)	0.694
Blood type (%)			0.739
O	115 (52.3)	374 (51.9)	
A	67 (30.5)	207 (28.7)	
B	30 (13.6)	105 (14.6)	
AB	7 (3.2)	34 (4.7)	
Unknown	1 (0.5)	1 (0.1)	
Time between symptom onset and randomization (%)			<0.001
<4 days	48 (21.8)	105 (14.6)	
4-7 days	81 (36.8)	355 (49.3)	
8-11 days	46 (20.9)	201 (27.9)	
12-15 days	30 (13.6)	40 (5.6)	
>15 days	15 (6.8)	19 (2.6)	
WHO score at randomization, 5 (%)	187 (85.0)	486 (67.4)	<0.001
High Risk (%)	162 (73.6)	615 (85.3)	<0.001
Diabetes (%)	83 (37.7)	249 (34.5)	0.431
Pulmonary (%)	22 (10.0)	75 (10.4)	0.964
Cardiovascular (%)	126 (57.3)	278 (38.6)	<0.001

Note: Corticosteroids include IV and PO corticosteroids at randomization.
Abbreviations: Q, quarter; SD, standard deviation; WHO, World Health Organization.

© 2021 American Medical Association. All rights reserved.

eTable 10: CCP SARS-CoV-2 IgG and Neutralizing titers by Quarters of Enrollment

	Total	2020 Q2	2020 Q3	2020 Q4	2021 Q5	*P* value [a]
Number randomized to CCP	468	84	60	199	125	
CCP IgG EC_{50}, median (IQR)	1:2,016 (916-4,229; n=359)	1:2,047 (677-5,400); n=69	1:1,610 (1,018-2,679); n=50	1:1,439 (611-3,054); n=146	1:3,596 (2,179-6,097); n=94	<0.0001
CCP Nt, median (IQR)	1:93 (48-213; n=352)	1:175 (76-379); n=58	1:73 (49-103); n=46	1:79 (35-178); n=166	1:106 (63-235); n=82	<0.0001

Abbreviations: CCP, COVID-19 Convalescent Plasma; EC_{50}, half-maximal effective concentration; IQR, interquartile range; Nt, neutralizing titer; Q, quarters; SARS-CoV-2, severe acute respiratory syndrome coronavirus.
[a] Kruskal-Wallis rank sum test

© 2021 American Medical Association. All rights reserved.

eTable 11: Baseline Characteristics and Day 14/28 Outcomes by Baseline SARS-CoV-2 IgG status and Treatment Group

	Baseline SARS-CoV-2 IgG Negative		Baseline SARS-CoV-2 IgG Positive	
	Placebo	CCP	Placebo	CCP
**Baseline characteristics**				
n	117	125	258	228
Enrollment quarters (%)				
2020 Q2	3 (2.6)	7 (5.6)	31 (12.0)	21 (9.2)
2020 Q3	17 (14.5)	21 (16.8)	27 (10.5)	28 (12.3)
2020 Q4	56 (47.9)	61 (48.8)	130 (50.4)	118 (51.8)
2021 Q5	41 (31.5)	36 (28.8)	70 (27.1)	61 (26.8)
Age (mean (SD))	63.8 (14.0)	61.2 (15.0)	60.7 (15.1)	60.4 (15.4)
Age (categorical) (%)				
<45 years	11 (9.4)	17 (13.6)	42 (16.3)	37 (16.2)
45-64 years	47 (40.2)	54 (43.2)	104 (40.3)	98 (43.0)
65-80 years	46 (39.3)	45 (36.0)	86 (33.3)	66 (28.9)
>80 years	13 (11.0)	9 (7.2)	26 (10.1)	27 (11.8)
Sex, Female (%)	59 (50.4)	44 (35.2)	105 (40.7)	88 (38.6)
Blood type (%)				
O	66 (56.4)	57 (45.6)	142 (55.0)	119 (52.2)
A	33 (28.2)	42 (33.6)	71 (27.5)	60 (26.3)
B	14 (12.0)	22 (17.6)	35 (13.6)	35 (15.4)
AB	4 (3.4)	4 (3.2)	9 (3.5)	14 (6.1)
Unknown	0 (0.0)	0 (0.0)	1 (0.4)	0 (0.0)
Time between symptom onset and randomization (%)				
<4 days	24 (20.5)	19 (15.2)	34 (13.2)	36 (15.8)
4-7 days	60 (51.3)	74 (59.2)	121 (46.9)	96 (42.1)
8-11 days	31 (26.5)	25 (20.0)	71 (27.5)	74 (32.5)
12-15 days	1 (0.9)	3 (2.4)	22 (8.5)	14 (6.1)
>15 days	1 (0.9)	4 (3.2)	10 (3.9)	7 (3.1)
NA	0 (0.0)	0 (0.0)	0 (0.0)	1 (0.4)
WHO score at randomization, 5 (%)	89 (76.1)	94 (75.2)	175 (67.8)	153 (67.1)
High Risk (%)	106 (90.6)	114 (91.2)	219 (84.9)	194 (85.1)
Remdesivir/Corticosteroids (%)				
None	13 (11.1)	10 (8.0)	32 (12.4)	27 (11.8)
Corticosteroids only	38 (32.5)	32 (25.6)	56 (21.7)	53 (23.2)
Remdesivir only	6 (5.1)	9 (7.2)	9 (3.5)	4 (1.8)
Both	60 (51.3)	74 (59.2)	161 (62.4)	144 (63.2)
Diabetes (%)	45 (38.5)	43 (34.4)	87 (33.7)	77 (33.8)
Pulmonary (%)	16 (13.7)	17 (13.6)	23 (8.9)	22 (9.6)
Cardiovascular (%)	55 (47.0)	46 (36.8)	103 (39.9)	90 (39.5)
Primary outcome				
WHO score at day 14 (%)				
0	6 (5.1)	7 (5.6)	19 (7.4)	22 (9.6)
1	18 (15.4)	15 (12.0)	46 (17.8)	43 (18.9)
2	38 (32.5)	37 (29.6)	88 (34.1)	79 (34.6)
3	14 (12.0)	18 (14.4)	29 (11.2)	24 (10.5)
4	2 (1.7)	5 (4.0)	11 (4.3)	2 (0.9)
5	4 (3.4)	6 (4.8)	22 (8.5)	13 (5.7)

© 2021 American Medical Association. All rights reserved.

6	8 (6.8)	12 (9.6)	7 (2.7)	10 (4.4)
7	2 (1.7)	3 (2.4)	1 (0.4)	1 (0.4)
8	5 (4.3)	8 (6.4)	6 (2.3)	7 (3.1)
9	9 (7.7)	6 (4.8)	14 (5.4)	6 (2.6)
10	9 (7.7)	7 (5.6)	13 (5.0)	19 (8.3)
NA	2 (1.7)	1 (0.8)	2 (0.8)	2 (0.9)
Secondary outcome				
WHO score at day 28 (%)				
0	20 (17.1)	18 (14.4)	55 (21.3)	56 (24.6)
1	22 (18.8)	16 (12.8)	51 (19.8)	46 (20.2)
2	30 (25.6)	38 (30.4)	72 (27.9)	65 (28.5)
3	9 (7.7)	16 (12.8)	27 (10.5)	14 (6.1)
4	1 (0.9)	4 (3.2)	4 (1.6)	1 (0.4)
5	1 (0.9)	4 (3.2)	5 (1.9)	4 (1.8)
6	2 (1.7)	1 (0.8)	4 (1.6)	4 (1.8)
7	3 (2.6)	1 (0.8)	2 (0.8)	2 (0.9)
8	3 (2.6)	1 (0.8)	4 (1.6)	1 (0.4)
9	2 (1.7)	7 (5.6)	6 (2.3)	5 (2.2)
10	21 (17.9)	18 (14.4)	26 (10.1)	28 (12.3)
NA	3 (2.6)	1 (0.8)	2 (0.8)	2 (0.9)

Note: Corticosteroids include IV and PO corticosteroids at randomization.
Abbreviations: NA, not available; Q, quarter; SD, standard deviation; WHO, World Health Organization.

© 2021 American Medical Association. All rights reserved.

eTable 12: Adverse events and Serious Adverse events

	Placebo	CCP	*P* value
n	473	468	
Adverse events (%)			
Transfusion reaction			
TACO	0 (0.0)	0 (0.0)	
TRALI	0 (0.0)	0 (0.0)	
Transfusion reaction (other than TRALI or TACO) (%)	2 (0.4)	8 (1.7)	0.06
Arterial thromboembolism	11 (2.3)	8 (1.7)	0.64
Venous thromboembolism	31 (6.6)	37 (7.9)	0.45
Patients with any adverse events (excluding other transfusion reactions)	39 (8.2)	44 (9.4)	0.57
Bleeding	35 (7.4)	41 (8.8)	0.47
Infection	124 (26.2)	110 (23.5)	0.37
Hospital readmission	67 (14.2)	59 (12.6)	0.50

Abbreviations: CCP, COVID-19 convalescent plasma; TACO, transfusion-associated circulatory overload; TRALI, transfusion-related acute lung injury.

© 2021 American Medical Association. All rights reserved.

eFigure 1: Trace Plots of Model Convergence

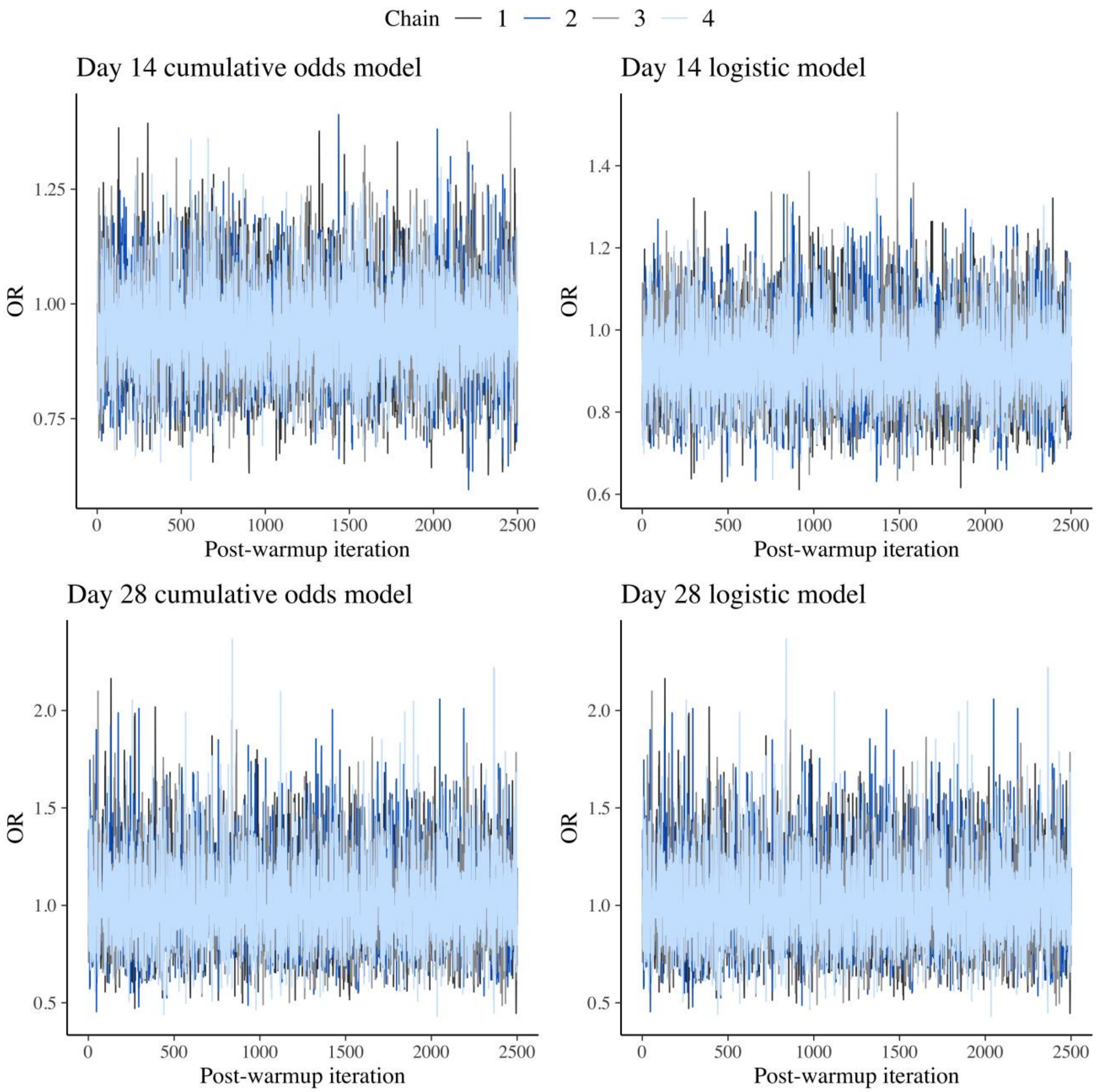

© 2021 American Medical Association. All rights reserved.

eFigure 2. Observed cumulative probability with predicted 95% credible interval by treatment group.

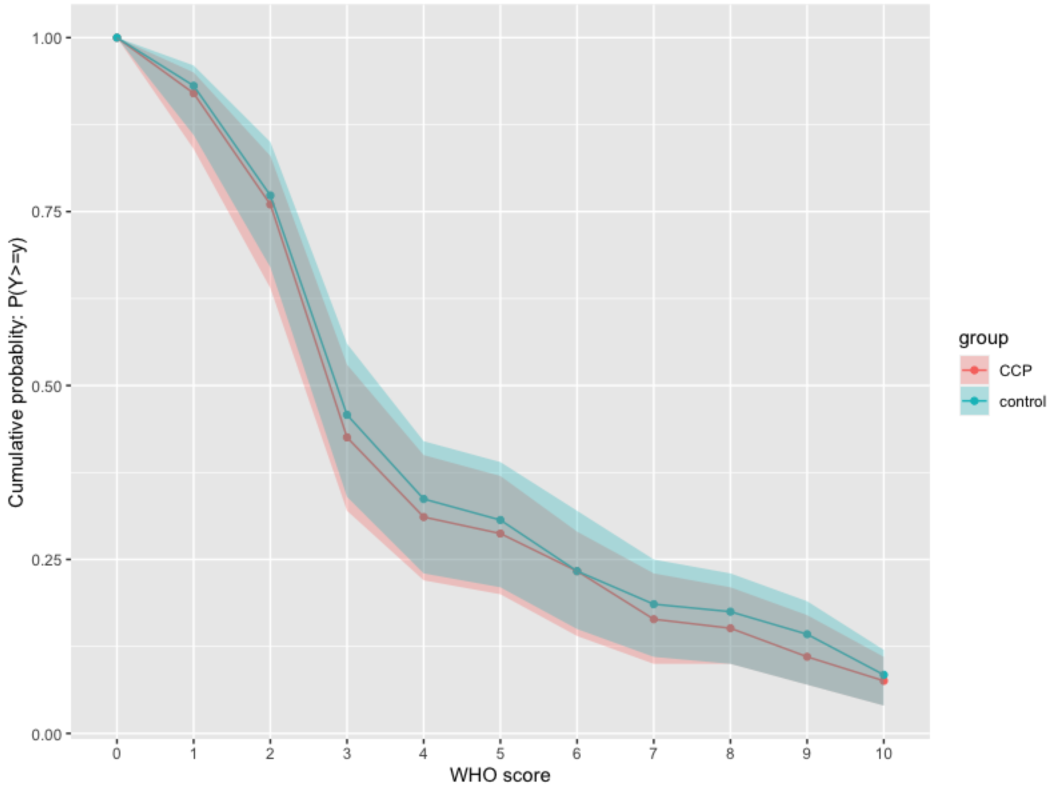

© 2021 American Medical Association. All rights reserved.

eFigure 3. Clinical Outcomes among Patients Treated with Convalescent Plasma and Placebo 14 and 28 Days after Randomization by Enrollment Quarter

Distribution of clinical status at 14 and 28 days after randomization by enrollment quarter based on 11-point ordinal scale shown by cumulative OR (curves) and 11 point WHO scores (stacked bars). ECMO, extracorporeal membrane oxygenation; HFNC, high flow nasal cannula; MV, mechanical ventilation; NIV, non-invasive ventilation Stacked bar blank cells < 3%.

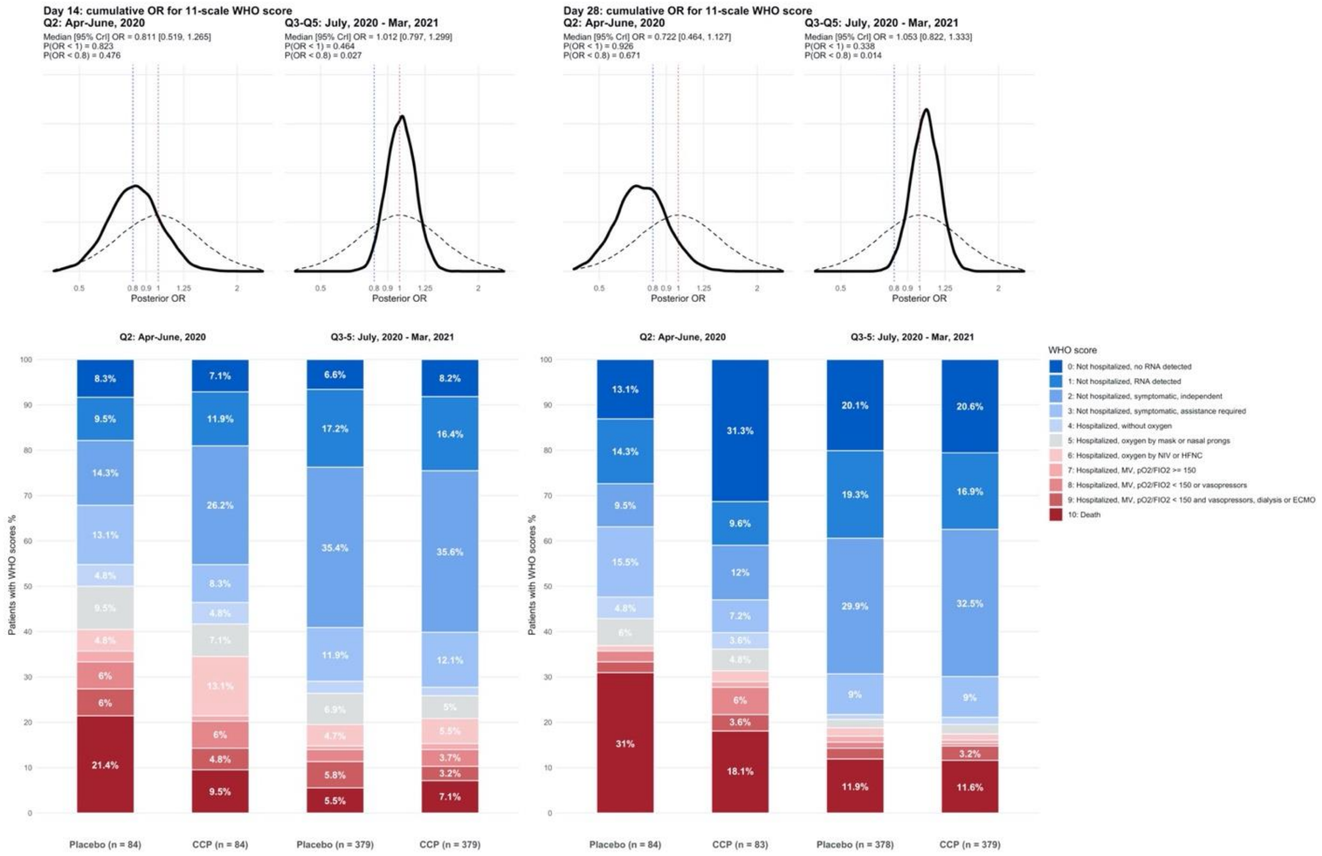

© 2021 American Medical Association. All rights reserved.

eFigure 4: Cumulative OR for WHO ordinal scale and OR for mortality at Day 14 in indicated subgroups

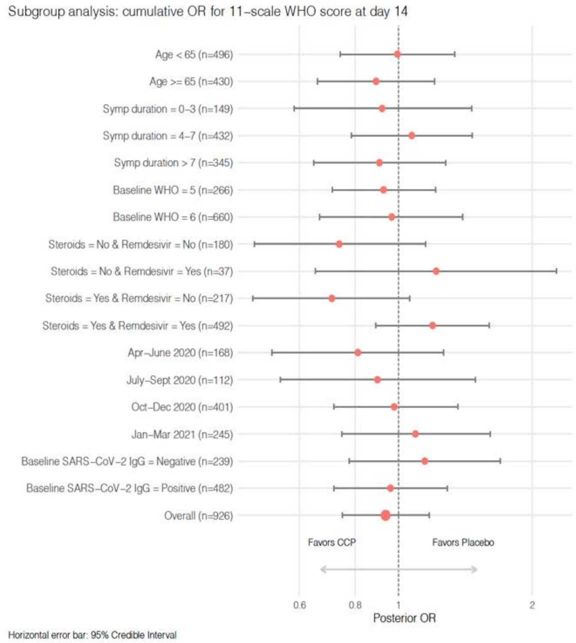

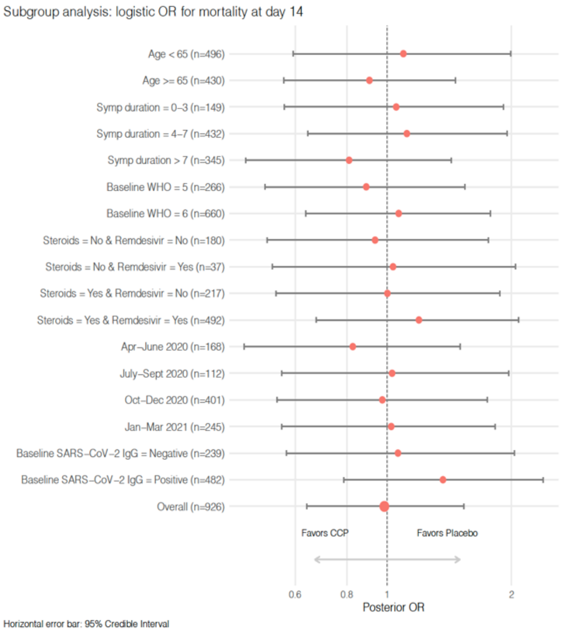

© 2021 American Medical Association. All rights reserved.

eFigure 5: Cumulative OR for WHO ordinal scale and OR for mortality at Day 28 in indicated subgroups

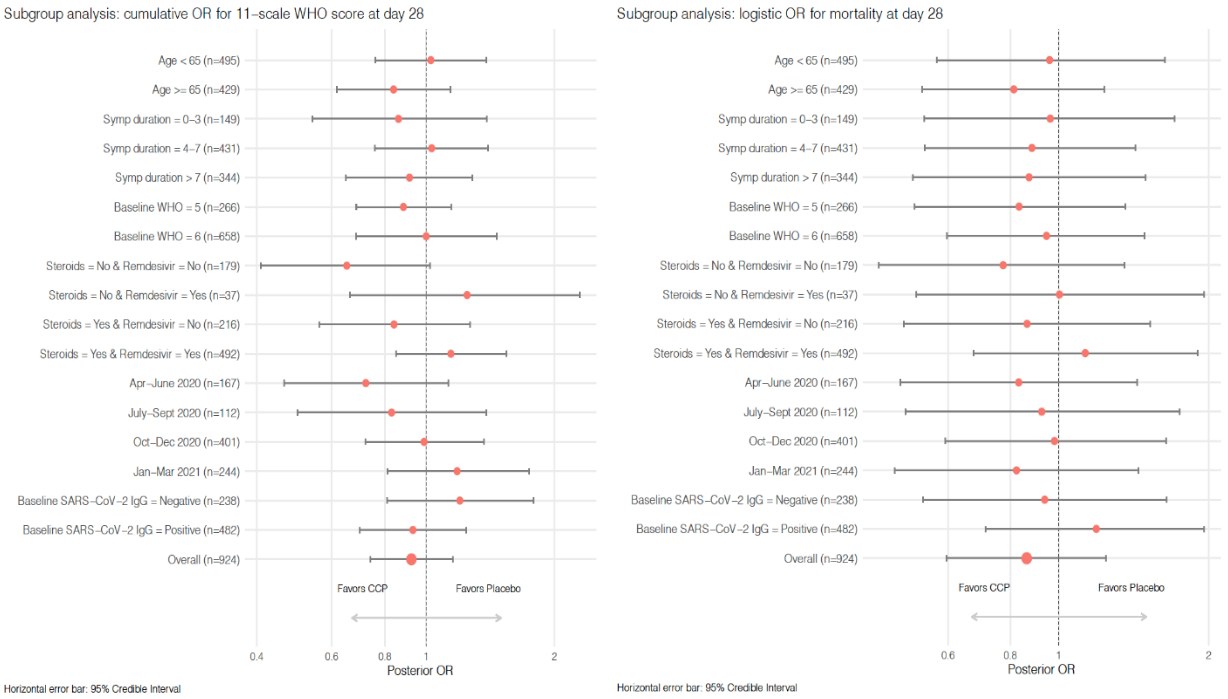

© 2021 American Medical Association. All rights reserved.

eFigure 6: Posterior Probability of Mortality at Day 14 of placebo and CCP recipients in indicated subgroups without adjustment for any covariates

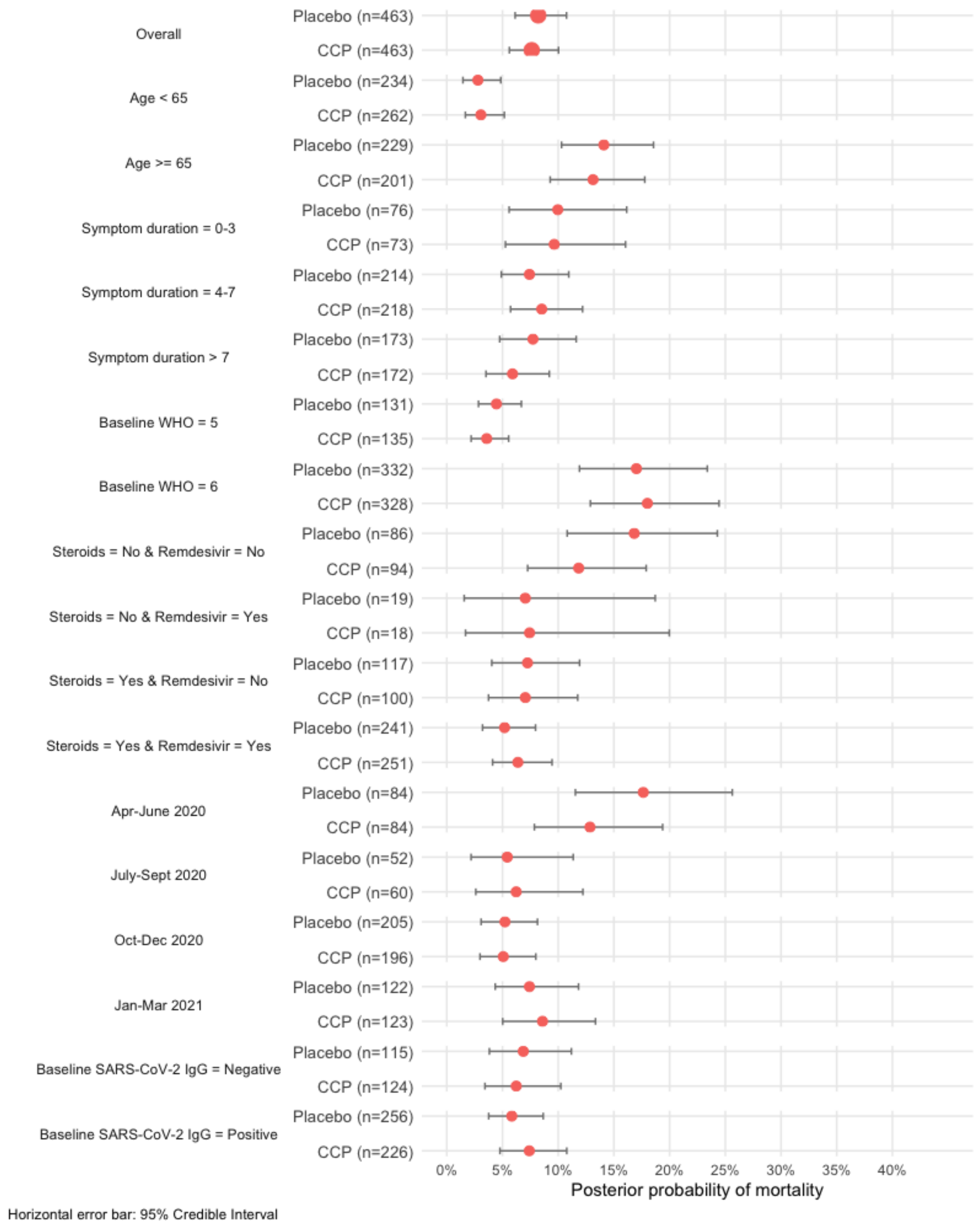

© 2021 American Medical Association. All rights reserved.

eFigure 7: Posterior Probability of Mortality at Day 28 of placebo and CCP recipients in indicated subgroups without adjustment for any covariates

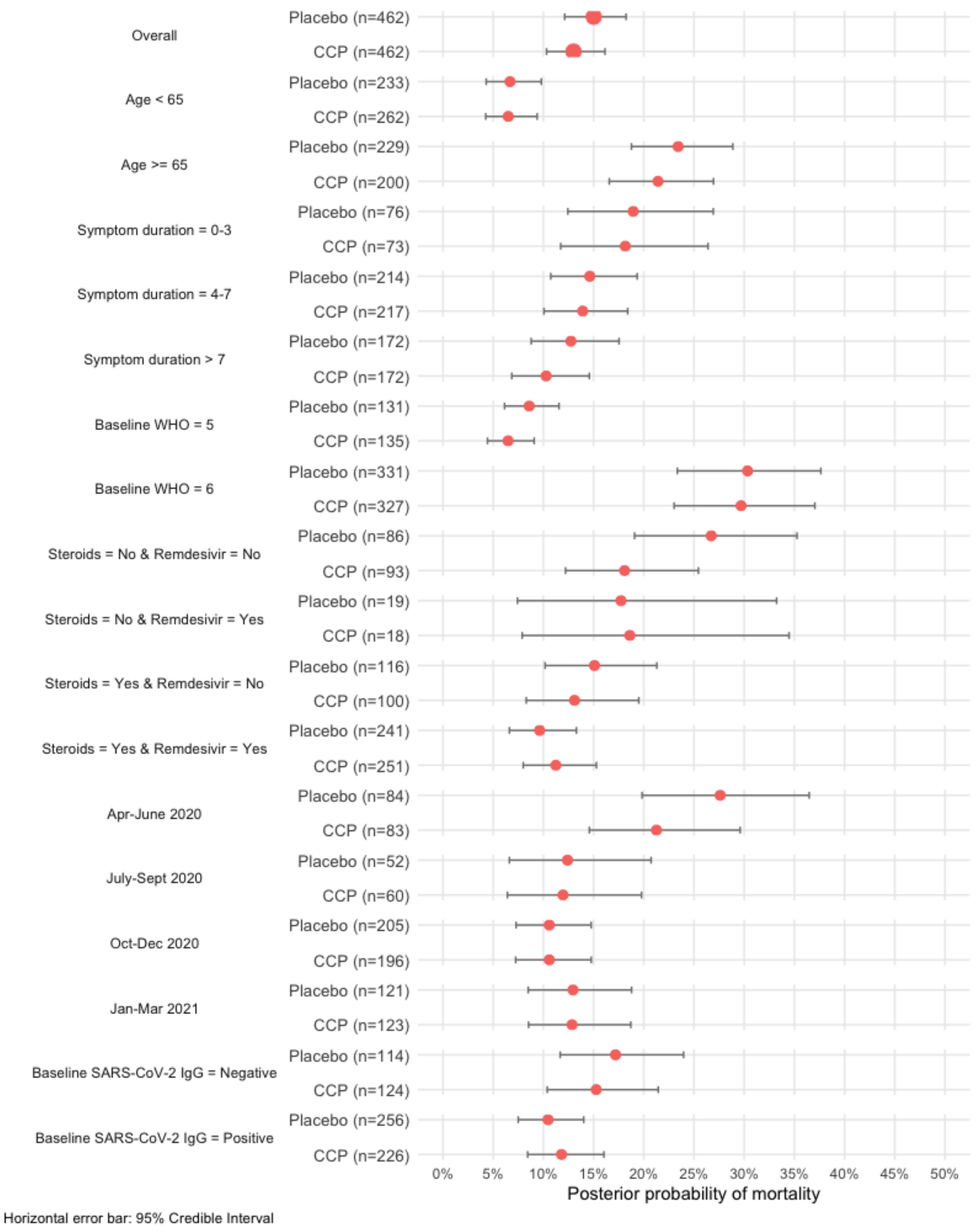

© 2021 American Medical Association. All rights reserved.

eFigure 8: Clinical Outcome in Placebo and CCP Groups Dichotomized by Median CCP SARS-CoV-2 IgG EC_{50} at 14 and 28 days after randomization

Distribution of clinical status of placebo and CCP recipients based on WHO score at 14 (top) and 28 (bottom) days after randomization. SARS-CoV-2 IgG EC_{50} was dichotomized at the median (1:2,016); values <1:2,016 were shown as low EC_{50}; values >1:2,016 shown as high EC_{50}. N=473 (placebo); 468 (CCP); 180 (low EC_{50}); 179 (high EC_{50}). EC_{50} indicates half-maximal effective concentration. Blank cells < 3%.

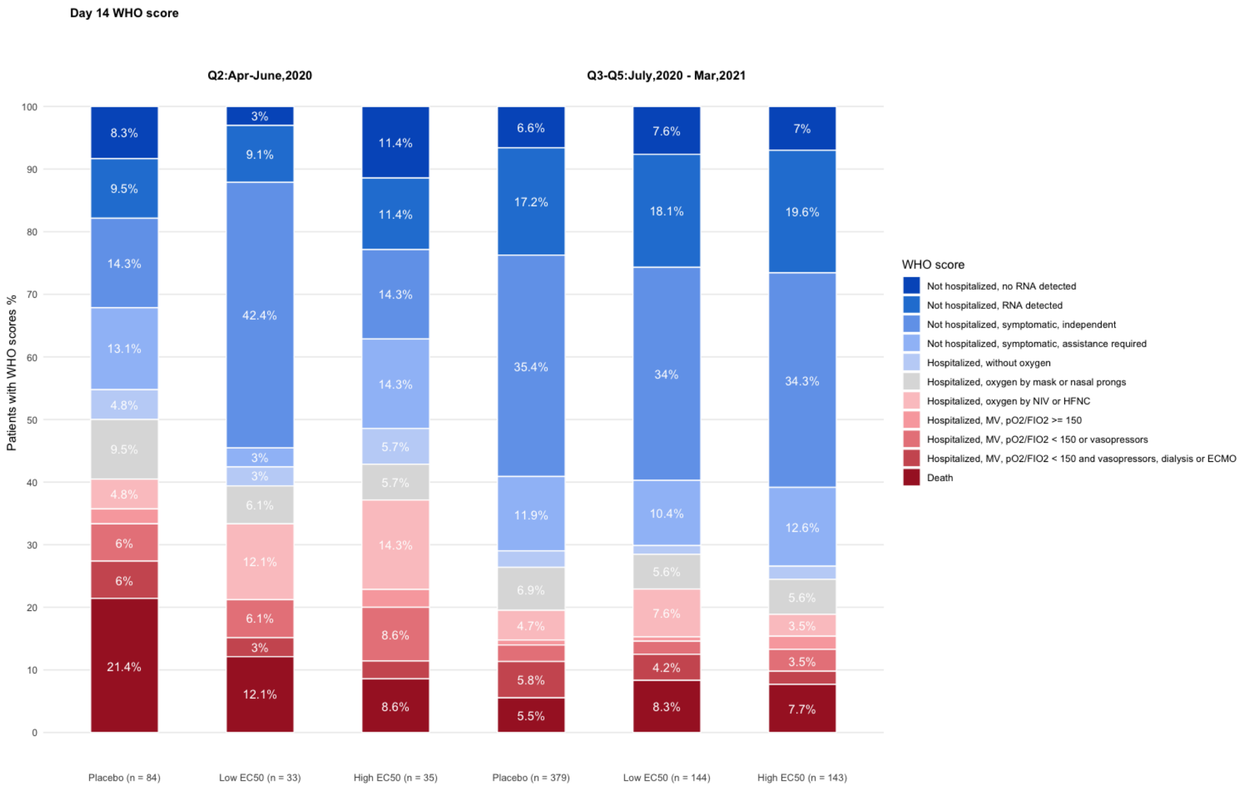

© 2021 American Medical Association. All rights reserved.

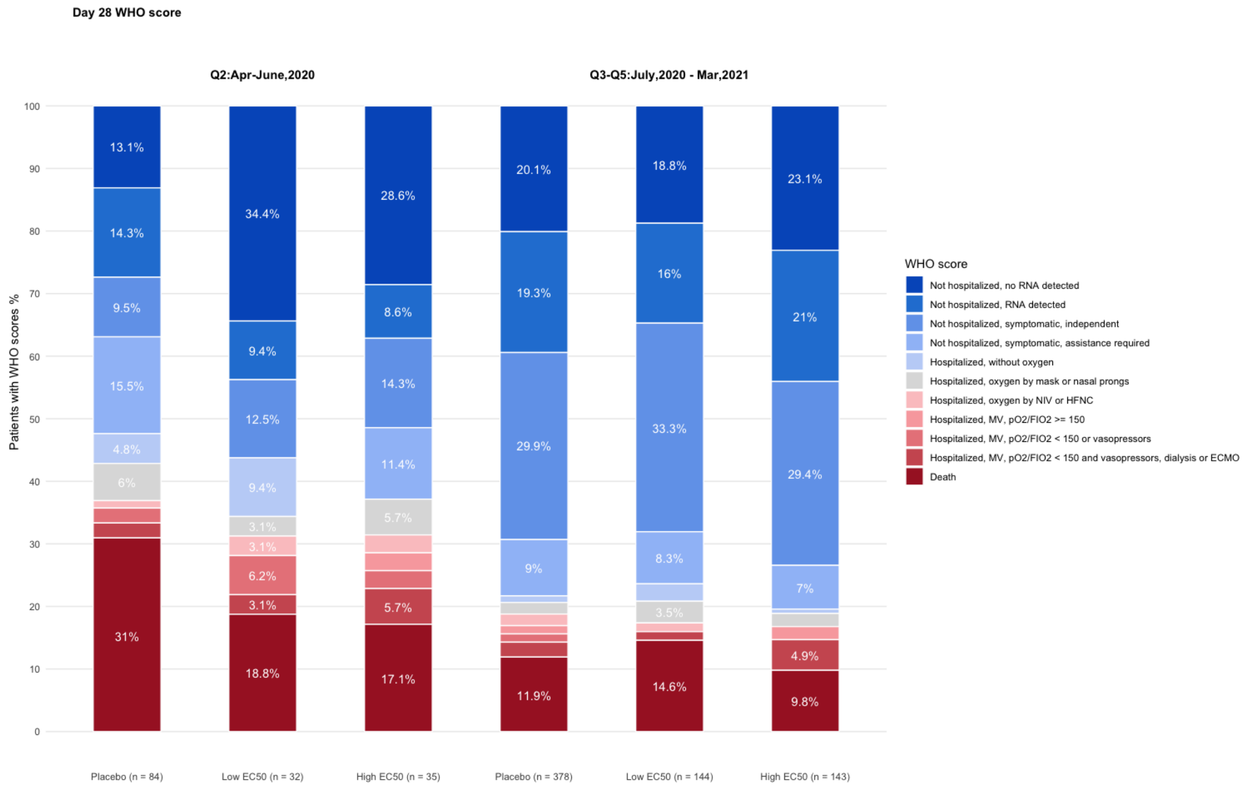

© 2021 American Medical Association. All rights reserved.

eReferences

1. A minimal common outcome measure set for COVID-19 clinical research. *Lancet Infect Dis.* 2020;20(8):e192-e197.
2. Yoon HA, Bartash R, Gendlina I, et al. Treatment of Severe COVID-19 with Convalescent Plasma in Bronx, NYC. *JCI Insight.* 2021.
3. Bortz RH, 3rd, Florez C, Laudermilch E, et al. Single-Dilution COVID-19 Antibody Test with Qualitative and Quantitative Readouts. *mSphere.* 2021;6(2).
4. Recommendations for Investigational COVID-19 Convalescent Plasma, 2020. Available from: https://www.fda.gov/media/141480/download. Last accessed Sept. 22, 2021.
5. New York SARS-CoV Microsphere Immunoassay for Antibody Detection. https://www.fda.gov/media/137541/download. Last accessed Sept. 22, 2021.
6. Herrera NG, Morano NC, Celikgil A, et al. Characterization of the SARS-CoV-2 S Protein: Biophysical, Biochemical, Structural, and Antigenic Analysis. *ACS Omega.* 2021;6(1):85-102.
7. Wrapp D, Wang N, Corbett KS, et al. Cryo-EM structure of the 2019-nCoV spike in the prefusion conformation. *Science.* 2020;367(6483):1260-1263.
8. Dieterle ME, Haslwanter, D., Bortz III., R.H., Wirchnianski, A.S., Lasso,, G. V, O., Abbasi, S.A., Fels, J.M., Laudermilch, E., Florez, C., Mengotto, A., Kimmel, D.,, Malonis RJ, Georgiev, G., Quiroz, J., Barnhill, J., Pirofski, L.-a., Daily, J.P., Dye, J.M., Lai, J.R.,, Herbert AS, Chandran, K., Jangra, R.K. A replication-competent vesicular stomatitis virus for studies of SARS-CoV-2 spike-mediated cell entry and its inhibition. *Cell Host and Microbe (2020).* 2020.

© 2021 American Medical Association. All rights reserved.

9. Gelman A, John B. Carlin, Hal S. Stern, David B. Dunson, Aki Vehtari, and Donald B. Rubin. Bayesian data analysis. *CRC press, 2013.*

© 2021 American Medical Association. All rights reserved.
