# Supplementary material for: Efficacy and Safety of COVID-19 Convalescent Plasma in Hospitalized Patients: A Randomized Clinical Trial
Source: JAMA Intern Med. 2021 Dec 13;182(2):1–12. doi: 10.1001/jamainternmed.2021.6850 (PMC8669605; doi:10.1001/jamainternmed.2021.6850)
Supplement: Supplement 3. — Statistical Analysis Plan [file jamainternmed-e216850-s003.pdf]

Statistical Analysis Plan

TRIAL FULL TITLE	Convalescent plasma to limit coronavirus associated complications: a randomized blinded phase 2 study comparing the efficacy and safety of anti-SARS-COV-2 plasma to placebo in COVID-19 hospitalized patients (CONTAIN COVID-19)
SAP VERSION	1.1
SAP VERSION DATE	3/23/2021
STUDY STATISTICIAN	Andrea B. Troxel
ASSOCIATED PROTOCOL VERSION	3.2
STUDY PRINCIPAL INVESTIGATOR	Mila Ortigoza & Liise-anne Pirofski
SAP AUTHOR(S)	Keith S. Goldfeld & Andrea B. Troxel

History of changes

Version	Date	Modification(s)
1.0	01/18/2021	First complete version
1.1	03/23/2021	Added brief description of exploratory analyses (Section 9.2.6)

1 SAP Signatures

I give my approval of the attached SAP entitled ***Convalescent plasma to limit coronavirus associated complications: a randomized blinded phase 2 study comparing the efficacy and safety of anti-SARS-COV-2 plasma to placebo in COVID-19 hospitalized patients*** dated 3/23/2021.

Statistician (Author)

Name: Andrea B. Troxel

Signature: Andrea B. Troxel

Date: 3/23/2021

Statistician Reviewer (as applicable)

Name: Keith S. Goldfeld

Signature: [illegible]

Date: 3/23/2021

Principal Investigator

Name: Mila Ortigoza & Liise-anne Pirofski

Type text here

Signature: [illegible] [illegible]

Date: 3/23/2021

Contents

1 SAP Signatures 3

2 Abbreviations and Definitions 6

3 Introduction 7
- 3.1 Preface . . . 7
- 3.2 Scope of the Analyses . . . 7

4 Study Objectives and Endpoints 7
- 4.1 Study Objectives . . . 7
- 4.2 Endpoints . . . 7

5 Study Methods 8
- 5.1 General Study Design and Plan . . . 8
- 5.2 Inclusion and Exclusion Criteria and General Study Population . . . 8
- 5.3 Randomization and Blinding . . . 9
- 5.4 Study Assessments . . . 9

6 Sample Size 9

7 General Analysis Considerations 10
- 7.1 Timing of Analyses . . . 10
- 7.2 Analysis Populations . . . 10
  - 7.2.1 Intention to Treat . . . 10
  - 7.2.2 Safety Population . . . 10
- 7.3 Covariates and Subgroups . . . 10
- 7.4 Missing Data . . . 10
- 7.5 Interim Analyses and Data Monitoring . . . 10
  - 7.5.1 Purpose of Interim Analyses . . . 10
  - 7.5.2 Planned Schedule of Interim Analyses . . . 11
  - 7.5.3 Stopping Rules . . . 11
  - 7.5.4 Practical Measures to Minimize Bias . . . 11
  - 7.5.5 Documentation of Interim Analyses . . . 11

8 Summary of Study Data 12

9 Efficacy Analyses 13
- 9.1 Primary Efficacy Analysis . . . 13
  - 9.1.1 WHO score at 14 days . . . 13
  - 9.1.2 Rationale for prior distributions of primary analyses . . . 14
    - 9.1.2.1 Level-specific intercepts/cut points . . . 14
    - 9.1.2.2 Site-specific effects . . . 14
    - 9.1.2.3 Between-site variation . . . 14
    - 9.1.2.4 Covariate coefficients . . . 14
    - 9.1.2.5 Treatment effect . . . 15
- 9.2 Secondary Efficacy Analyses . . . 15
  - 9.2.1 Effect of duration of COVID-19 symptoms prior to CP transfusion . . . 15
  - 9.2.2 Additional models to assess heterogeneous treatment effects . . . 15
  - 9.2.3 Effect of donor CP antibodies on the efficacy of CP . . . 15
  - 9.2.4 Precision medicine analysis . . . 16
  - 9.2.5 Mortality and time to hospital discharge . . . 16
  - 9.2.6 Additional exploratory analyses . . . 16
- 9.3 Summary of analyses . . . 16

10 Safety Analyses **17**

11 Tables, Listings, and Figures **18**

12 References **20**

2 Abbreviations and Definitions

AFib	atrial fibrillation
ARDS	acute respiratory distress syndrome
CHF	chronic heart failure
COPD	chronic obstructive pulmonary disease
COVID-19	coronavirus disease
CP	convalescent plasma
CPO	cumulative proportional odds
DSMB	Data & Safety Monitoring Board
ECMO	extracorporeal membrane oxygenation
eGFR	estimated glomerular filtration rate
FIO2	fraction of inspired oxygen
ILD	interstitial lung disease
ITT	intention-to-treat
NIV	noninvasive ventilation
NP	nasopharyngeal
NYHA	New York Heart Association
OR	odds ratio
OSA	obstructive sleep apnea
pO2	partial pressure of oxygen
RCT	randomized controlled trial
SARS-CoV-2	Severe Acute Respiratory Syndrome Coronavirus 2
SpO2	oxygen saturation
SS	saline solution (defined as half-, quarter- or normal saline)
TACO	transfusion-associated circulatory overload
TRALI	transfusion-related acute lung injury

3 Introduction

3.1 Preface

There are currently few treatment options for coronavirus disease (COVID-19) and the related pneumonia, caused by Severe Acute Respiratory Syndrome Coronavirus 2 (SARS-CoV-2) beyond supportive care. Human convalescent plasma is a treatment option for COVID-19 and could be rapidly available when there are enough people who have recovered and donate high titer (anti-SARS-CoV-2) neutralizing immunoglobulin-containing plasma. Use of convalescent plasma is a form of passive antibody therapy that involves the administration of antibodies to a given agent to a susceptible individual for the purpose of preventing or treating the infectious disease it causes. When used for therapy, antibody is most effective when administered shortly after the onset of symptoms. The goal is to treat patients who are sick enough to warrant hospitalization but do not have severe respiratory disease and/or ARDS.

3.2 Scope of the Analyses

These analyses will assess the efficacy and safety of ***therapeutic convalescent plasma*** *(CP) for COVID-19 hospitalized patients.*

4 Study Objectives and Endpoints

4.1 Study Objectives

The purpose of this study is to evaluate the efficacy and safety of convalescent plasma from people who have recovered from COVID-19 containing antibodies to SARS-CoV-2 versus control (SS) to prevent worsening respiratory status or death in hospitalized patients with COVID-19 who are within 3 days of presentation to the hospital or within 7 days of symptom onset.

4.2 Endpoints

The primary outcome is health status at 14 days using the WHO 11-point ordinal scale for clinical improvement which ranges from 0 (uninfected) to 10 (death). Health status at 28 days will also be assessed. Effect size will be measured as the cumulative odds ratio comparing treatment to placebo control, estimated using a Bayesian cumulative proportional odds model that adjusts for initial status.

Secondary outcomes will include, but will not be limited to, assessments of heterogeneous treatment effects based on baseline characteristics or conditions, effect of the *level* of convalescent (donor) plasma antibodies, mortality, and time to hospital discharge.

The safety outcomes include (1) transfusion-related acute lung injury (TRALI), (2) transfusion-associated circulatory overload (TACO), and (3) transfusion reaction (other than TRALI or TACO).

Patient State	Score	Description
Uninfected	0	No viral RNA detected
Ambulatory	1	Asymptomatic; viral RNA detected
	2	Symptomatic; independent
	3	Symptomatic; assistance needed
Hospitalized: mild disease	4	No oxygen therapy
	5	Oxygen by mask or nasal prongs
Hospitalized: severe disease	6	Oxygen by NIV or high flow
	7	Intubation & vechanical ventilation; pO2/FIO2 $\geq$ 150 or SpO2/FIO2 $\geq$ 200
	8	Mechanical ventilation pO2/FIO2 $<$150 (SpO2/FIO2 $<$200) or vasopressors
	9	Mechanical ventilation pO2/FIO2 $<$150 and vasopressors, dialysis, or ECMO
Death	10	Dead

5 Study Methods

5.1 General Study Design and Plan

- This is a multi-center randomized controlled trial (RCT) using Bayesian modeling and interim monitoring for efficacy and safety issues.
- Efficacy will be based on the posterior probability distribution of the effectiveness estimate, the cumulative odds ratio comparing treatment and control.
- Control arm will be saline solution.
- This trial will be double-blinded.
- Randomization will be stratified by site and health risk status (high vs. low).
- Patients will be randomized after hospitalization, and the intervention will commence within one day of randomization.
- Baseline measurements will be collected at randomization, CP will be provided immediately following randomization, and outcome measurements will be collected at day 14 and day 28. Concomitant medications at time of randomization will be recorded. CP-related adverse events will be recorded daily.

5.2 Inclusion and Exclusion Criteria and General Study Population

Inclusion Criteria

1. Patients $\geq$ 18 years of age
2. Hospitalized with laboratory confirmed COVID-19
3. One or more of the following respiratory signs or symptoms: cough, chest pain, shortness of breath, fever, oxygen saturation $\leq$ 94%, abnormal CXR/CT imaging
4. Hospitalized for $\leq$ 72 hours OR within 7 days from first signs of illness
5. On supplemental oxygen, non-invasive ventilation or high-flow oxygen
6. Patients may be on other randomized controlled trials of pharmaceuticals for COVID -19 and patients who meet eligibility criteria will not be excluded on this basis.

Exclusion Criteria

1. Receipt of pooled immunoglobulin in past 30 days
2. Contraindication to transfusion or history of prior reactions to transfusion blood products
3. Invasive mechanical ventilation or extracorporeal membrane oxygenation (ECMO)
4. Volume overload secondary to congestive heart failure or renal failure
5. Unlikely to survive past 72 hours from screening based on the assessment of the investigator
6. Unlikely to follow-up or clinical assessment will be hindered by patient's poor functional status

5.3 Randomization and Blinding

Subjects will be randomized in a 1:1 ratio to receive 1 unit of study product (CP) or placebo control (equivalent volume of SS). Randomization will be stratified by site and by risk category.

High risk: Subjects with age ≥60 years **or** age <60 *and* at least one of the following:

- Chronic pulmonary conditions (COPD, OSA, ILD, etc)
- Chronic heart conditions (CHF with NYHA ≥ class 2, AFib, ischaemic heart disease, etc)
- Hypertension
- Chronic kidney disease with eGFR < 60 mL/min
- Body Mass Index ≥ 35
- Diabetes mellitus
- Immunosuppression (CD4 < 200, on immunosuppressive medications for autoimmune conditions, cancers, solid or stem cell transplants, steroids such as prednisone >10mg/day or equivalent)

Lower risk: Subjects with age <60 *and* without the presence of any high risk factors listed above.

5.4 Study Assessments

The WHO 11-point score will be collected at Day 14 following randomization and also at Day 28. See the study protocol for a detailed schedule of study assessments.

6 Sample Size

The planned total sample size for the trial is 1000 subjects.

We estimated an initial sample size using simulations assuming a frequentist, two-sided Type I error rate (alpha) of 0.05 and 80% power. We made the following additional assumptions based on information available at the time of estimation:

- 30% incidence of worsening respiratory status (10% death and 20% on invasive mechanical ventilation or ECMO, respectively) and 10% of discharged alive in the control group estimated by current data from our hospital.
- 1.8 odds ratio (OR) of worsening respiratory status between the control group and the anti-SARS-CoV-2 convalescent plasma group; this approximately corresponds to a 13% absolute reduction in incidence of worsening respiratory status (5% death and 12% on invasive mechanical ventilation or ECMO, respectively) and 5% absolute increase of discharged alive using anti-SARS-CoV-2 convalescent plasma.
- Very few subjects will be randomized and fail to receive study plasma infusion or will be lost to follow-up and have missing data for the primary endpoint.

We initially estimated that a sample size of 300 patients (150 in each arm) would be sufficient to detect the specified difference in clinical status between the two arms with a power of at least 0.8; therefore, the planned total sample size of 1000 subjects is deemed more than sufficient.

7 General Analysis Considerations

7.1 Timing of Analyses

Continuous monitoring and analysis by the DSMB will occur approximately every two weeks. The final analysis will occur when the DSMB has recommended stopping the study for safety, efficacy, or harm and the investigators have agreed to accept this recommendation.

7.2 Analysis Populations

7.2.1 Intention to Treat

The primary analysis will estimate the full ITT effect based on all patients who were randomized.

7.2.2 Safety Population

All patients who received any study treatment.

7.3 Covariates and Subgroups

The primary outcome model will include the covariates age, sex, baseline WHO score, and duration of time since COVID-19 symptom onset as control variables (only main effects).

Secondary pre-planned analysis will be conducted to investigate the potential differential effect of the following covariates (i.e., interaction effects):

- Sex
- Age
- Duration of time since COVID-19 symptoms onset at randomization
- WHO score at baseline
- Health risk status at baseline
- Antibodies in the donor CP

Exploratory analysis will be conducted to evaluate the potential effect of comorbid conditions and concomitant medications at randomization on the safety, efficacy and harm of CP.

7.4 Missing Data

We anticipate minimal missingness in the covariates and/or outcome data.

7.5 Interim Analyses and Data Monitoring

7.5.1 Purpose of Interim Analyses

Given the urgency to identify effective therapeutic options for COVID-19 patients in this world-wide pandemic, frequent or continuous monitoring of the accumulating data is absolutely necessary. Continuous monitoring, using Bayesian stopping rules that allow for real-time decisions without the penalties for multiple data looks and α-spending associated with the classic RCT monitoring approach, is an efficient approach to this problem. At each interim analysis, the posterior distribution of the parameter describing the treatment effect will be reported (graphically and analytically) and the prespecified stopping criteria will guide

the recommendations of the DSMB. The Bayesian monitoring approach enables straightforward, actionable rules for efficacy, futility, harm and safety, all of which can incorporate information accrued across all studies. The process involves estimation of the posterior probability of a favorable or unfavorable odds ratio, and the stopping rules will be based on the posterior probability that the odds ratio exceeds a pre-specified threshold.

7.5.2 Planned Schedule of Interim Analyses

The interim analyses will be conducted every two weeks.

7.5.3 Stopping Rules

The primary analysis of the WHO 11-point ordinal scale 0-10 at day 14 will be based on a cumulative proportional odds (CPO) model (see Section 9.1). The estimated log odds will be modeled as a function of a CP treatment indicator, the covariates, and the random effects for sites. We denote the parameter of interest (the overall treatment effect) in the CPO model using δ. Details about the analytic model and the initial priors are found in Section 9.1.

We propose considerations for stopping the study based on the following posterior probabilities for the odds ratios ($OR = e^{\delta}$).

Stopping for efficacy

$$P\,(OR < 1) \geq 0.95 \quad \text{and} \quad P\,(OR < 0.8) \geq 0.50$$

Stopping for harm

$$P\,(OR > 1) \geq 0.80$$

Stopping for safety

The logistic regression model for evaluating safety is described in Section 10. We denote the parameter of interest (the overall CP effect) in the CPO model using θ.

We propose stopping for safety based on the posterior probability for the odds ratio (OR) of adverse events in the CP condition compared to the control condition ($OR_{ae} = e^{\theta}$). The proposed stopping rule enforces considerations for stopping for safety reasons, even if only a relatively weak safety signal is detected.

$$P(OR_{ae} > 1) \geq 0.75.$$

7.5.4 Practical Measures to Minimize Bias

All interim analyses will be conducted by an unblinded biostatistician who is coordinating with the DSMB. The DSMB will review the results and if there is a consensus among the DSMB members that an action should be taken, the recommendation will be shared with the RCT team.

7.5.5 Documentation of Interim Analyses

Snapshots of the data available at each interim analysis will be preserved, as will all documentation of analysis plans, programming code, and reports provided at each interim analysis. It will be possible to fully recreate the decision process from the trial archive at a time when any limitations of access to information by blinded statisticians becomes unnecessary.

8 Summary of Study Data

All demographic and baseline variables will be reported by study arm. All continuous variables will be summarized by means, standard deviations, medians, and interquartile ranges (IQR). Frequencies and percentages (based on the non-missing sample size) will be reported for all categorical measures (including binary measures). All summary tables will be structured with a column for each treatment arm, including any missing observations.

Data	Type
**Demographics**	
Age in years	# ; NA = not available
Sex	0 = male; 1 = female; 2 = other; NA = not available
Blood group	0 = O; 1 = A; 2 = B; 3 = AB; NA = not available
**Status at enrollment**	
Quarter of randomization	1 = Jan-Mar, 2020; 2 = Apr-Jun, 2020; 3 = Jul-Sep, 2020; 4 = Oct-Dec, 2020; 5 = Jan-Mar, 2021; 6 = Apr-Jun, 2021; 7 = Jul-Sep, 2021; 8 = Oct-Dec, 2021
Days since symptoms onset at time of randomization	1 = 0 to 3; 2 = 4 to 6; 3 = 7 to 10; 4 = 11 to 14; 5 = more than 14; NA = not available
Days since COVID-19 diagnosis at randomization	#; NA = not available
Baseline WHO score	4 = Hospitalized - no O2; 5 = Hospitalized - O2 with mask; 6 = Hospitalized - O2 with non-invasive ventilation
Time from randomization to first infusion	#; NA = not available (Enter 0 if the patient was randomized to standard of care and if first infusion is performed on the day of randomization)
Randomized treatment received	0 = no; 1 = yes; NA = not available
Risk status	0 = lower risk; 1 = higher risk
**Disease history**	
History of diabetes (all types)	0 = no; 1 = yes; NA = not available
History of pulmonary disease	0 = no; 1 = yes; NA = not available
History of cardiovascular disease	0 = no; 1 = yes; NA = not available

The following table lists concomitant medication at the time of randomization.

Medications at randomization	Type
Hydroxychloroquine	0 = no, 1 = yes, NA = not available
Antibacterial	0 = no, 1 = yes, NA = not available
Antiviral (not Remdesivir)	0 = no, 1 = yes, NA = not available
Remdesivir	0 = no, 1 = yes, NA = not available
Anti-inflammatory (non-steroids)	0 = no, 1 = yes, NA = not available
Steroids	0 = no, 1 = yes, NA = not available
Antithrombotic	0 = no, 1 = yes, NA = not available
Double blind Remdesivir	0 = no, 1 = yes, NA = not available
Double blind Hydroxichloroquine	0 = no, 1 = yes, NA = not available
Convalescent plasma outside RCT	0 = no, 1 = yes, NA = not available
Any double blind RCT other than Hydroxychloroquine or Remdesivir	0 = no, 1 = yes, NA = not available

9 Efficacy Analyses

9.1 Primary Efficacy Analysis

The primary efficacy outcome is clinical status at 14 days after randomization, assessed using the WHO 11-point ordinal outcome scale.

9.1.1 WHO score at 14 days

The analysis will be a cumulative odds model for the ordinal WHO score at 14 days. If Y is the WHO 11-point scale, $(Y = 0, ..., 10)$, let π_y be the probability of observing stuatus y:

$$\pi_y = P\left(Y = y\right), y = 0,\ \ldots,\ 10,\ \sum_{y=0}^{10} \pi_y = 1$$

and p_y be the probability of observing status y or worse:

$$p_y = P\left(Y \geq y\right) = \sum_{s=y}^{10} \pi_s,\ y = 1,\ \ldots,\ 10 \qquad (1)$$

Assume that data from J sites are available and that there are n_j subjects at the jth site, $j = 1, \ldots, J$. Denote the outcome for the ith patient from the jth trial on the 11-point WHO ordinal COVID-19 scale at day 14 by $Y_{ij} = y$, $y = 0, \ldots, 10$, and that patient's baseline covariates (a vector of length m) by $\boldsymbol{x}_{ij}$. $\boldsymbol{x}_{ij}$ will include the following: *age, sex, baseline WHO-11 score, risk stratum, quarter of enrollment, and days since symptom onset at randomization.* T_{ij} is a treatment indicator, where $T_{ij} = 1$ if patient i in site j was randomized to CP, $T_{ij} = 0$ otherwise.

The following cumulative odds model for Y_{ij} will be considered:

$$\begin{aligned}
\text{logit}\left(p_{ijy}\right) &= \tau_y + b_j + \boldsymbol{\beta}\boldsymbol{x}_{ij} + \delta T_{ij} \\
\tau_y &\sim t_{\text{student}}\left(\text{df=3}, \mu = 0, \sigma = 8\right), \qquad \text{monotone} \\
b_j &\sim \text{Normal}\left(0, \sigma = \eta\right) \\
\eta &\sim \text{Exponential}\left(\text{rate} = 0.1\right) \\
\boldsymbol{\beta} &\sim \text{Normal}\left(\mathbf{0}, \Sigma = 2.5^2 I_{m\times m}\right) \\
\delta &\sim \text{Normal}\left(0, \sigma = 0.354\right) \\
OR &= \exp\left(\delta\right)
\end{aligned} \tag{2}$$

The log odds defined from the cumulative probabilities of the control arm are estimated by τ_y from (2), which corresponds the intercept associated with level y on the ordinal WHO outcome $Y = y$, $y = 1, \ldots, 10$. All τ_y, $y = 1, \ldots, 10$, satisfy the monotonicity requirements for the intercepts of the proportional odds model. b_j represents the jth site-specific effect on the cumulative log odds. β is a vector of covariate effects. δ is the treatment effect, the log odds ratio comparing the odds of a worse outcome under CP with the odds of a worse outcome with SS.

9.1.2 Rationale for prior distributions of primary analyses

The prior distributions used in (2) were selected based on extensive simulations that had three goals: (i) to understand the behavior of the estimating procedure in a variety of realistic situations, (ii) to compare the inferences from the Bayesian analysis with Bayesian monitoring to frequentist analysis with frequentist interim monitoring (with 3 to 5 interim looks) and to anchor the prior distributions to results consistent with inferences from frequentist analyses; this was an identified goal because the clinical community is still more familiar with and more comfortable with inferences from frequentists analyses; and (iii) to assess convergence issues and sensitivity of the posterior distributions to variations in the postulated priors. The simulations were performed in R [11] and Stan [13].

The overarching philosophy has been to be conservative (skeptical priors) with respect to the outcome measures, to be less conservative (less skeptical priors) for parameters that will not influence decision making but are important to estimate, and to be flexible with respect to nuisance parameters to ensure stable model fitting. While the details presented below are specific to the primary analyses, the same rationale and philosophy are applicable to the secondary analyses as well. Here we provide brief summary of the choices in (2):

9.1.2.1 Level-specific intercepts/cut points

τ_y are the level-specific cut points of the cumulative odds model; they are constrained to be monotonically increasing. The priors for these parameters are based on a modified $t-$distribution, and are set to be weakly informative. Stan implements this through the use of an inverse transformation function, where the MCMC draws are on an unconstrained parameter space and transformed back to the desired monotonic parameters [12].

9.1.2.2 Site-specific effects

The site-specific effects b_j are presumed to have a Normal distribution with mean 0 and an unknown variance η, as is commonly assumed in a mixed effects model. η will be estimated from the data.

9.1.2.3 Between-site variation

The variation across sites η will be estimated using a weakly informative prior exponential distribution that is constrained to be greater than 0.

9.1.2.4 Covariate coefficients

The covariate coefficients $\boldsymbol{\beta}$ have each a weakly informative prior on the log-odds scale, corresponding to little prior information about the effects, and allowing the data to quickly prevail in the estimation. Note

that the (relatively) large variance of the Normal distribution ($\sigma = 2.5$) makes the prior weakly informative without the need for heavy tails that the $t-$distributions allow. This is a case where the Normal distribution and the t-distribution result in similar posterior distributions for the parameters, but the Normal distribution provides somewhat better model convergence.

9.1.2.5 Treatment effect

To be conservative and to maintain desired operating characteristics of the model, we impose a skeptical prior on the overall treatment effect δ that is centered around 0. The $\sigma = 0.354$ of the Normal prior for the δ (on the log-odds ratio scale) corresponds to 95% interval for the efficacy odds ratio being between 0.5 and 2.

9.2 Secondary Efficacy Analyses

9.2.1 Effect of duration of COVID-19 symptoms prior to CP transfusion

The effect of duration of symptoms prior to treatment with CP will also be explored in detail. The CONTAIN study collects this information in the format of ordinal variable: 0-3 days; 4-6 days; 7-10 days; 11-14 days and days >14. To explore the impact of symptoms duration on the CP effect on the WHO 11-point score, an extended version of the model described for the primary outcome (Section 9.1) will be developed. The extended model will include study specific treatment by symptom duration interaction parameters γ_s, $s \in \{2, 3, 4, 5\}$ in the Bayesian model as follows:

$$
\begin{aligned}
\text{logit}\left(p_{ijy}\right) &= \tau_y + b_j + \boldsymbol{\beta}\boldsymbol{x}_{ij} + T_{ij}\left(\delta + \gamma_s d_{ijs}\right), && s = 2, \ldots, 5 \text{ for symptom duration strata} \\
\tau_y &\sim t_{\text{student}}\left(\text{df=3}, 0, \sigma = 8\right), && \text{monotone} \\
b_j &\sim \text{Normal}\left(0, \sigma = \eta\right) \\
\eta &\sim \text{Exponential}\left(\text{rate} = 0.1\right) \\
\boldsymbol{\beta} &\sim \text{Normal}\left(\mathbf{0}, \Sigma = 2.5^2 I_{m\times m}\right) \\
\delta &\sim \text{Normal}\left(0, \sigma = 0.354\right) \\
\gamma_s &\sim t_{\text{student}}\left(df = 3, 0, 2.5\right)
\end{aligned} \tag{3}
$$

This model differs from the primary model in one key respect - the effect of the treatment can depend on the duration of symptoms. d_{ijs} is an indicator variable, where $d_{ijs} = 1$ when patient i in site j was categorized as s with respect to symptom duration, $d_{ijs} = 0$ otherwise. For example, the effect for patients in category 1 is δ, for patients in category 2 it is $\delta + \gamma_2$, for patients in category 3 it is $\delta + \gamma_3$, etc.

9.2.2 Additional models to assess heterogeneous treatment effects

We will estimate four additional models using the structure of Model 3 to assess the possible difference in treatment effects due to these additional factors that have also been hypothesized to have a strong impact on the effectiveness of CP: age, sex, baseline health status as measured by the WHO-baseline score, and risk stratum.

9.2.3 Effect of donor CP antibodies on the efficacy of CP

The primary analysis of CONTAIN addresses the question *whether treatment with convalescent plasma (yes/no) is efficacious against a simple saline solution.* The statistical model to address this primary question is discussed in Section 9.1.

A second and equally important question that CONTAIN aims to address is whether the level of antibodies in the CP matters and if so, how the amount of antibodies is related to the efficacy of treatment with CP. We plan on exploring two different models. The first model will treat the antibody levels (or some transformation

of the antibody measures) as a continuous predictor of the log-odds outcome. The second will be based on a latent threshold model, described conceptually in [3] and [4].

9.2.4 Precision medicine analysis

We will employ existing and newly developed methodologies to identify biosignatures for response to CP treatment. Biosignatures are patient characteristics or combination of such characteristics that are associated with the heterogeneity of treatment effects. In its simplest case, the biosignature is continuous variable (e.g., a linear combination of baseline patient characteristics) that has a strong (large in magnitude, significant) interaction with the treatment indicator in the model for the outcome. [9, 8]. The methodologies developed for discovery of such biosignatures for treatment response are under the rubric of developing optimal treatment decision rules, i.e., give the particular treatment only to patients who are likely to benefit from it (based on what is known about the patient at the time of treatment decision making). Precision medicine is an active area of current developments and new approaches are constantly being developed to address ever more complex clinical circumstances. [7, 10, 14, 1]

9.2.5 Mortality and time to hospital discharge

The tertiary outcomes include overall mortality (time to death) and time to hospital discharge. The analysis of overall mortality will be based on a (stratified) log-rank test with adjustment for RCT. Cox proportional hazards model will be employed to adjust for the covariates in the comprehensive covariates list (age, sex, etc) and to evaluate interactions of baseline characteristics with treatment. The proportional hazards assumption will be evaluated using the method of cumulative martingale residuals[6].

The variable of time to discharge is defined as the duration from randomization to hospital discharge to home, acute and long-term rehab facilities. Death before discharge is a competing risk event that precludes a successful discharge and thus will be properly accounted in the analysis of time to discharge. Gray's test[5] will be used to compare the subdistribution hazards (cumulative incidence function, CIF) of time-to-discharge between treatment groups. Fine-Gray regression model[2] will be employed to estimate treatment effect on the CIF adjusting for the comprehensive list of covariates.

9.2.6 Additional exploratory analyses

Additional analyses will be conducted to investigate important aspects of the treatment, exposures, and biometric measures. These will include but will not be limited to (1) convalescent (donor) plasma and participant anti-SARS-CoV-2 titer and neutralizing titer, (2) convalescent (donor) plasma and participant SARS-CoV-2 antibody profiles and functional assays, (3) rates, levels and duration of SARS-CoV-2 RNA in NP swabs, (4) SARS CoV2 variants, (5) clinical status at other visit days, mortality, and rates of discharge, (6) lymphocytes, neutrophils, and cytokines, and (7) the moderating effect of concomitant medications - including corticosteroids, Remdesivir, and anticoagulants - on convalescent (donor) plasma treatment effects.

9.3 Summary of analyses

The following table provides a schematic representation of all the analyses that we plan to conduct for five outcomes: **WHO score at day 14**, **WHO score at day 28**, **mortality at day 14**, **mortality at day 28**, and **time to discharge**. Stopping rules for efficacy will be based on the non-interaction models of the primary outcome **WHO score at day 14**. Stopping will be considered if the primary endpoint is met; the interim analyses will be further informed by the *non-interaction* secondary analyses. The interaction models will be estimated after the study has stopped.

Planned Analyses

Description	Day	Adjustment	Interaction models				
			Age	Sex	Symptoms Duration	WHO Baseline	Risk Stratum
**Primary analysis: comparison of CP vs. Saline Solution**							
1. WHO score: cum. prop. OR	14	parsimonious[1]	x	x	x	x	x
**Secondary analyses: comparison of CP vs. Saline Solution**							
2. WHO score: cum. prop. OR	14	expanded[2]	x	x	x	x	x
3. WHO score: cum. prop. OR	28	expanded	x	x	x	x	x
**Tertiary analyses: comparison of CP vs. Saline Solution**							
4. All-cause mortality (yes/no)	14	expanded	x	x	x	x	x
5. All-cause mortality (yes/no)	28	expanded	x	x	x	x	x
6. Time to discharge		expanded	x	x	x	x	x
**Tertiary analyses: comparison of no AB, low AB, and not low AB**							
7. WHO score: cum. prop. OR	14	expanded	x	x	x	x	x
8. WHO score: cum. prop. OR	28	expanded	x	x	x	x	x
9. All-cause mortality (yes/no)	14	expanded	x	x	x	x	x
10. All-cause mortality (yes/no)	28	expanded	x	x	x	x	x
11. Time to discharge		expanded	x	x	x	x	x

[1] Parsimonious adjustment includes age, sex, WHO score at baseline, days since symptom onset, and risk stratum.
[2] Expanded adjustment also includes past history and concomitant medications before or at time of infusion.

10 Safety Analyses

We propose monitoring for safety based on adverse events related to the transfusion of plasma. Specifically, we will compare the CP and control conditions with respect to the proportion of patients who experienced at least one of the adverse events in Section 4.2: TRALI, TACO, TACO/TRALI/worsening COVID - overlap and undifferentiated reactions, and any other arterial thrombotic event or a venous thrombotic event.

The presence/absence of safety events is a binary outcome that we will analyze with logistic regression models. Let Z_{ij} be an indicator that the ith subject in the jth site experiencing a transfusion-related event. The effect of CP will be denoted by θ. The log odds of having the transfusion-related event in the SS arm is estimated by γ, which corresponds to the intercept.

The following logistic regression model will be used to model Z:

$$
\begin{aligned}
Z_{ij} &\sim \text{Binomial}\left(\boldsymbol{r}_i\right), & 0 < \boldsymbol{r}_i < 1 \\
\text{logit}\left(P\left(Z_{ij} = 1\right)\right) &= \gamma + b_j + \boldsymbol{\lambda}\boldsymbol{x}_{ij} + T_{ij}\theta \\
\gamma &\sim t_{\text{student}}\left(\text{df=}3, 0, \sigma = 2.5\right) \\
\boldsymbol{\lambda} &\sim \text{Normal}\left(\mathbf{0}, \Sigma = 5^2 I_{m\times m}\right) \\
\theta &\sim t_{\text{student}}\left(\text{df=}3, 0, \sigma = 5.0\right)
\end{aligned}
\tag{4}
$$

θ corresponds to the difference of log-odds of a saftey issue for CP and log-odds for control. Note that the prior for θ has a larger standard deviation than the prior for the effect of the treatment on the efficacy/harm. This prior for θ is considered weakly informative and would allow for the posterior distribution to follow the data with relatively smaller sample sizes.

11 Tables, Listings, and Figures

The following are shells of the tables we expect to produce for the interim and final analyses.

Table 0

**Site**	**Final sample size**	
	**Control Group**	**CP Group**
NYU	m_{11}	n_{11}
Einstein	m_{12}	n_{12}
Yale	m_{13}	n_{13}
...	...	...
**Total**	**M**	**N**

Table 1: Baseline demographic and clinical characteristics

**Variable**	**Control Group (total # = M)**	**CP Group (total # = N)**
Age (median, IQR)		
Female sex (n, %)		
Baseline WHO severity<br>4 - hospitalized/no O2 (n, %)<br>5 - hospitalized/O2 by mask or nasal prongs (n, %)<br>6 - hospitalized/O2 by non-invasive ventilation (n, %)		
Blood group<br>O (n, %)<br>A (n, %)<br>B (n, %)<br>AB (n, %)<br>Not available (n, %)		
Duration of symptoms onset at randomization<br>0-3 days (n, %)<br>4-6 days (n, %)<br>7-10 days (n, %)<br>11-14 days (n, %)<br>>14 days (n, %)		
Risk stratum<br>Lower risk (n, %)<br>Higher risk (n, %)		
Days since COVID-19 diagnosis at randomization (median, IQR)		
History of diabetes (all types) (n, %)		
History of pulmonary disease (all types) (n, %)		
History of cardiovascular disease (all types) (n, %)		

12 References

[1] Adam Ciarleglio, Eva Petkova, Todd Ogden, and Thaddeus Tarpey. Constructing treatment decision rules based on scalar and functional predictors when moderators of treatment effect are unknown. *Journal of the Royal Statistical Society. Series C, Applied statistics*, 67(5):1331, 2018.

[2] J.P. Fine and R.J. Gray. A proportional hazards model for the subdistribution of a competing risk. *Journal of the American Statistical Association*, 94:496–509, 1999.

[3] Keith S Goldfeld. A bayesian implementation of a latent threshold model. https://www.rdatagen.net/post/a-latent-threshold-model-to-estimate-treatment-effects/, Dec 2020.

[4] Keith S Goldfeld. A latent threshold model to dichotomize a continuous predictor. https://www.rdatagen.net/post/a-latent-threshold-model/, Nov 2020.

[5] R.J. Gray. A class of K-sample tests for comparing the cumulative incidence of a competing risk. *The Annals of statistics*, pages 1141–1154, 1988.

[6] D.Y. Lin, L.J. Wei, and Z. Ying. Checking the cox model with cumulative sums of martingale-based residuals,. *Biometrika*, 80(3):557–572, 1993.

[7] Susan A Murphy. Optimal dynamic treatment regimes. *Journal of the Royal Statistical Society: Series B (Statistical Methodology)*, 65(2):331–355, 2003.

[8] Hyung Park, Eva Petkova, Thaddeus Tarpey, and R Todd Ogden. A constrained single-index regression for estimating interactions between a treatment and covariates. *Biometrics*, 2020.

[9] Eva Petkova, R Todd Ogden, Thaddeus Tarpey, Adam Ciarleglio, Bei Jiang, Zhe Su, Thomas Carmody, Philip Adams, Helena C Kraemer, Bruce D Grannemann, et al. Statistical analysis plan for stage 1 embarc (establishing moderators and biosignatures of antidepressant response for clinical care) study. *Contemporary clinical trials communications*, 6:22–30, 2017.

[10] Min Qian and Susan A Murphy. Performance guarantees for individualized treatment rules. *Annals of statistics*, 39(2):1180, 2011.

[11] R Core Team. *R: A Language and Environment for Statistical Computing*. R Foundation for Statistical Computing, Vienna, Austria, 2020.

[12] Stan Development Team. Stan reference manual, 2020. Version 2.25 (available online).

[13] Stan Development Team. Stan modeling language users guide, 2020.

[14] Yingqi Zhao, Donglin Zeng, A John Rush, and Michael R Kosorok. Estimating individualized treatment rules using outcome weighted learning. *Journal of the American Statistical Association*, 107(499):1106–1118, 2012.
