# Supplementary material for: Efficacy and Safety of COVID-19 Convalescent Plasma in Hospitalized Patients: A Randomized Clinical Trial
Source: JAMA Intern Med. 2021 Dec 13;182(2):1–12. doi: 10.1001/jamainternmed.2021.6850 (PMC8669605; doi:10.1001/jamainternmed.2021.6850)
Supplement: Supplement 4. — Nonauthor Collaborators. The CONTAIN COVID-19 Study Group [file jamainternmed-e216850-s004.pdf]

Supplemental Online Content: Nonauthor Collaborators

*Indicates required information. Only first name, last name, and suffix will appear in PubMed.

*Group Name(s): The CONTAIN COVID-19 Study Group							
*First Name and Middle Initial(s)	*Last Name	*Suffix (eg, Jr, III)	Academic Degrees	Institution	Location (city, state/province, country)	Role or Contribution, eg, chair, principal investigator	Group (if more than 1 Group listed in the byline) and/or Subgroup (eg, Steering Committee)
Fatema Z	Rahman		BS	NYU Langone Health	New York, NY, USA	Associate Site Coordinator	
Adeyinka O	Ajayi		MD, MPH	NYU Langone Health	New York, NY, USA	Study Coordinator	
Sara L	Rodriguez		MBA	NYU Langone Health	New York, NY, USA	Regulatory Coordinator	
Ana G	Ledesma		BA	NYU Langone Health	New York, NY, USA	Data Coordinator	
Deborah	Keeling		MS	NYU Langone Health	New York, NY, USA	Project Finance	
Norka	Rappoport		BA	NYU Langone Health	New York, NY, USA	Project Finance	
Sam F	Ebel		JD, MPH	NYU Langone Health	New York, NY, USA	Project Contracts	
Jayne	Kim		PhD	NYU Langone Health	New York, NY, USA	Project Regulatory Coordinator	
Michelle	Chang		MS, MPH	NYU Langone Health	New York, NY, USA	Study Project Manager (previous)	
Kevin	Chan		MS	NYU Langone Health	New York, NY, USA	Study Project Manager (previous)	
Payal	Patel		BS	NYU Langone Health	New York, NY, USA	Lead Programmer	
Anne	Martocci		BA	NYU Langone Health	New York, NY, USA	Programmer	
Shivang	Dave		MS	NYU Langone Health	New York, NY, USA	Programmer	
Yousef	Darwish		BS	NYU Langone Health	New York, NY, USA	Programmer	
Monica	Taveras		BS	NYU Langone Health	New York, NY, USA	Study Data Manager (current)	
Victoria	Shoyelu		BS	NYU Langone Health	New York, NY, USA	Study Data Manager (previous)	
Patrick	Xin		MA, MS	NYU Langone Health	New York, NY, USA	Study Associate Data Manager	
Eduardo	Iturrate		MD, MSW	NYU Langone Health	New York, NY, USA	Site Associate Data Manager	
Lee C	Moldolsky		MSN, RN	NYU Langone Health – Long Island	Long Island, NY, USA	Transfusion Liaison	
Brian J	Raimondo		BSN	NYU Langone Health - Manhattan	New York, NY, USA	Transfusion Liaison	
Sarah	Mendez		EdD, RN, AOCNS	NYU Langone Health - Manhattan	New York, NY, USA	Transfusion Liaison	
Patricia	Hughes		MA, RN, NPD-BC OCN	NYU Langone Health - Manhattan	New York, NY, USA	Transfusion Liaison	
Stephanie	Sterling		MD	NYU Langone Health - Brooklyn	Brooklyn, NY, USA	Sub-Investigator	
Aaron S	Lord		MD, MSC	NYU Langone Health - Brooklyn	Brooklyn, NY, USA	Sub-Investigator	
Shadi	Yaghi		MD	NYU Langone Health - Brooklyn	Brooklyn, NY, USA	Sub-Investigator	
Karen	Veloso		MD	NYU Langone Health – Long Island	Long Island, NY, USA	Sub-Investigator	
Masooma	Sheikh		MD	NYU Langone Health – Long Island	Long Island, NY, USA	Sub-Investigator	
Erica	Visconti-Ferrara		DO	NYU Langone Health – Long Island	Long Island, NY, USA	Sub-Investigator	
Andrew	Fleming		MD	NYU Langone Health	New York, NY, USA	Sub-Investigator	
Heekoung	Youn		RN	NYU Langone Health, Bellevue	New York, NY, USA	Research Nurse	
Baby	Jane Fran		RN	NYU Langone Health, Bellevue	New York, NY, USA	Research Nurse	
Rosario	Medina		RN, MA	NYU Langone Health, Bellevue	New York, NY, USA	Research Nurse	
Renee	McKell		BS	NYU Langone Health – Brooklyn	Brooklyn, NY, USA	Site Coordinator	
Saila	Khan		MBBS	NYU Langone Health – Long Island	Long Island, NY, USA	Site Coordinator	
Tanya	Hamilton		MS	NYU Langone Health - Manhattan	New York, NY, USA	Blood Bank, Rand Key Holder	
Carlos J	Sanchez		BS, MT	NYU Langone Health - Brooklyn	Brooklyn, NY, USA	Blood Bank, Rand Key Holder	
Nandini H	Patel		MS, MLS(ASCP)	NYU Langone Health – Long Island	Long Island, NY, USA	Blood Bank	
Levi	Cleare		BA	Albert Einstein College of Medicine	Bronx, NY, USA	Study Research Scientist	
Olivia	Vergnolle		PhD	Albert Einstein College of Medicine	Bronx, NY, USA	Study Research Scientist	
Antonio	Nakouzi		BS	Albert Einstein College of Medicine	Bronx, NY, USA	Study Research Scientist	
Gregory	Quevedo		BS	Albert Einstein College of Medicine	Bronx, NY, USA	Study Research Scientist	
Robert H	Bortz	III	MS	Albert Einstein College of Medicine	Bronx, NY, USA	Study Research Scientist	
Ariel S	Wirchnianski		MS	Albert Einstein College of Medicine	Bronx, NY, USA	Study Research Scientist	
Catalina	Florez		PhD	Albert Einstein College of Medicine	Bronx, NY, USA	Study Research scientist	

Supplemental Online Content: Nonauthor Collaborators

*Indicates required information. Only first name, last name, and suffix will appear in PubMed.

*First Name and Middle Initial(s)	*Last Name	*Suffix (eg, Jr, III)	Academic Degrees	Institution	Location (city, state/province, country)	Role or Contribution, eg, chair, principal investigator	Group (if more than 1 Group listed in the byline) and/or Subgroup (eg, Steering Committee)
Rachelle	Babb		PhD	Albert Einstein College of Medicine	Bronx, NY, USA	Study Research scientist	
Jennifer	Ayala		BA	Albert Einstein College of Medicine	Bronx, NY, USA	Regulatory Coordinator	
K. Zoe	Tsagaris		MS	Albert Einstein College of Medicine	Bronx, NY, USA	Regulatory Coordinator	
Andria	James		BS	Albert Einstein College of Medicine	Bronx, NY, USA	Regulatory coordinator	
Isaiah	Eke		MD, MPH	Albert Einstein College of Medicine	Bronx, NY, USA	Site Coordinator	
Aisha	Obeidallah		BA	Albert Einstein College of Medicine	Bronx, NY, USA	Site Coordinator	
Oana A	Sandu		MD	Albert Einstein College of Medicine	Bronx, NY, USA	Data Coordinator	
Sophie	Sohval		MD	Albert Einstein College of Medicine	Bronx, NY, USA	Data Coordinator	
Leana	Serrano-Rahman		MPH	Albert Einstein College of Medicine	Bronx, NY, USA	Blood bank	
Joan	Uehlinger		MD	Albert Einstein College of Medicine	Bronx, NY, USA	Blood bank	
Rachel	Bartash		MD	Albert Einstein College of Medicine	Bronx, NY, USA	Sub-Investigator	
Aya	Al-Abduladheem		MD	Albert Einstein College of Medicine	Bronx, NY, USA	Sub-Investigator	
Inessa	Gendlina		MD, PhD	Albert Einstein College of Medicine	Bronx, NY, USA	Sub-Investigator	
Carol	Sheridan		RN, MSN	Albert Einstein College of Medicine	Bronx, NY, USA	Research nurse	
Anna	Bortnick		MD, PhD, MS	Albert Einstein College of Medicine	Bronx, NY, USA	Sub-Investigator	
Jeremy	Eichler		BS	Albert Einstein College of Medicine	Bronx, NY, USA	Site Coordinator	
Rachel	Kaufman		BS	Albert Einstein College of Medicine	Bronx, NY, USA	Site Coordinator	
Sarah	Yukelis		BA	Albert Einstein College of Medicine	Bronx, NY, USA	Site Coordinator	
Michael	Pennock		MD	Albert Einstein College of Medicine	Bronx, NY, USA	Site Coordinator	
Michelle	Goggin		PhD	Albert Einstein College of Medicine	Bronx, NY, USA	Regulatory Coordinator	
Christine	Shen		BS	Albert Einstein College of Medicine	Bronx, NY, USA	Data Coordinator	
Jayabhargav	Annam		BS	Albert Einstein College of Medicine	Bronx, NY, USA	Data Coordinator	
Ahmed	Khokhar		MD	Albert Einstein College of Medicine	Bronx, NY, USA	Data Coordinator	
Daniel	Barboto		MD	Albert Einstein College of Medicine	Bronx, NY, USA	Data Coordinator	
Brianna J	Lally		MD	Albert Einstein College of Medicine	Bronx, NY, USA	Data Coordinator	
Audrey	Lee		MS	Albert Einstein College of Medicine	Bronx, NY, USA	Data Coordinator	
Max	Lee		MD	Albert Einstein College of Medicine	Bronx, NY, USA	Data Coordinator	
Xiuyi A	Yang		MS	Albert Einstein College of Medicine	Bronx, NY, USA	Data Coordinator	
Stephanie	Allen		MD	Albert Einstein College of Medicine	Bronx, NY, USA	Data Coordinator	
Avinash	Malaviya		MS	Albert Einstein College of Medicine	Bronx, NY, USA	Data Coordinator	
Omar	Moussa		MD	Albert Einstein College of Medicine	Bronx, NY, USA	Data Coordinator	
Rosa	Park		MS	Albert Einstein College of Medicine	Bronx, NY, USA	Data Coordinator	
Reise	Sample		BFA	Albert Einstein College of Medicine	Bronx, NY, USA	Data Coordinator	
Andrea	Bae		MS	Albert Einstein College of Medicine	Bronx, NY, USA	Data Coordinator	
Galit	Benoni		MD	Albert Einstein College of Medicine	Bronx, NY, USA	Data Coordinator	
Jeff	LaFleur		MA	Albert Einstein College of Medicine	Bronx, NY, USA	Translational Core Administrator	
Lindsie L	Boerger		BA	Yale University Medical Center	New Haven, CT, USA	Lead Site Coordinator	
Lisa D	Baker		RN, BSN, OCN	Yale University Medical Center	New Haven, CT, USA	Lead Site Coordinator	
Martha A	Luther		BSN, MPH	Yale University Medical Center	New Haven, CT, USA	Site Coordinator	
Lirim S	Ameti		MD	Yale University Medical Center	New Haven, CT, USA	Sub-Investigator	
Neima	Briggs		MD, PhD	Yale University Medical Center	New Haven, CT, USA	Sub-Investigator	
Marjorie R	Golden		MD	Yale University Medical Center	New Haven, CT, USA	Sub-Investigator	
Michael	Gormally		MD, PhD	Yale University Medical Center	New Haven, CT, USA	Sub-Investigator	
Gloria S	Huang		MD	Yale University Medical Center	New Haven, CT, USA	Sub-Investigator	
Raymond M	Johnson		MD, PhD	Yale University Medical Center	New Haven, CT, USA	Sub-Investigator	

Supplemental Online Content: Nonauthor Collaborators

*Indicates required information. Only first name, last name, and suffix will appear in PubMed.

*First Name and Middle Initial(s)	*Last Name	*Suffix (eg, Jr, III)	Academic Degrees	Institution	Location (city, state/province, country)	Role or Contribution, eg, chair, principal investigator	Group (if more than 1 Group listed in the byline) and/or Subgroup (eg, Steering Committee)
Alyssa R	Morrison		BS	Yale University Medical Center	New Haven, CT, USA	Sub-Investigator	
Michele	Montagna-Hill		MS	Yale University Medical Center	New Haven, CT, USA	Site Data manager	
Brooke N	Rivera		MSW	Yale University Medical Center	New Haven, CT, USA	Site Data manager	
Grace M	Cortezzo		none	Yale University Medical Center	New Haven, CT, USA	Site Data manager	
Kay B	Debski		BS	Yale University Medical Center	New Haven, CT, USA	Regulatory Coordinator	
Amy	Nicoletti		MS	Yale University Medical Center	New Haven, CT, USA	Regulatory Coordinator	
Kerry	DeBenedictis		BA, MA	Yale University Medical Center	New Haven, CT, USA	Research Assistant	
Rivcah	Davis		MS, BB(ASCP)	Johns Hopkins Medical Center	Baltimore, MD, USA	Blood Bank, Rand Key Holder	
Christi	Marshall		B.S. MT(ASCP), CQA(ASQ)	Johns Hopkins Medical Center	Baltimore, MD, USA	Blood Bank, Rand Key Holder	
Miriam Andrea	Duque Cuartas		MD	UHealth Tower, Jackson Memorial	Miami, FL, USA	Blood Bank, Rand Key Holder	
Laura	Beauchamps		MD	Jackson Memorial	Miami, FL, USA	Sub-Investigator	
Jovanna	Bertran-Lopez		MD, MPH	Jackson Memorial	Miami, FL, USA	Sub-Investigator	
Jose	Gonzales Zamora		MD	Jackson Memorial	Miami, FL, USA	Sub-Investigator	
Maria	Delgado-Lelievre		MD	UHealth Tower	Miami, FL, USA	Sub-Investigator	
Sheela	Dominguez		MBA, MA	UHealth Tower, Jackson Memorial	Miami, FL, USA	Site Project Manager	
Chin Chin	Lee		MSPH, MS, BS	UHealth Tower, Jackson Memorial	Miami, FL, USA	Site Project Manager and Regulatory Coordinator	
Halina	Kusack		RN, BBM, MSN	UHealth Tower, Jackson Memorial	Miami, FL, USA	Research Nurse Manager	
Vela	Karakeshishyan		MD, BSN	UHealth Tower, Jackson Memorial	Miami, FL, USA	Site Coordinator	
Americo	Hajaz		MD	UHealth Tower, Jackson Memorial	Miami, FL, USA	Site Coordinator	
Dasmany	Deniz		BS	UHealth Tower, Jackson Memorial	Miami, FL, USA	Site Coordinator	
Giovanni	Garcia		BHSA	UHealth Tower, Jackson Memorial	Miami, FL, USA	Site Coordinator	
Katheryn	Dae		MD, BS	UHealth Tower	Miami, FL, USA	Site Coordinator	
Patricia	Blenet		MSN, BA	UHealth Tower	Miami, FL, USA	Site Coordinator	
Deborah	Jaffe		MS, BS	UHealth Tower	Miami, FL, USA	Site Coordinator	
Lourdes	Olson		MD	UHealth Tower	Miami, FL, USA	Site Coordinator	
Diane	Sabogal		MSN, BSN	UHealth Tower	Miami, FL, USA	Site Coordinator	
Olivia	Blust		BA	UHealth Tower	Miami, FL, USA	Site Coordinator	
Veronica	Del Prete Perez		MD	Jackson Memorial	Miami, FL, USA	Site Coordinator	
Claudia	Bornia		MD	Jackson Memorial	Miami, FL, USA	Site Coordinator	
Vanessa	Rodriguez-Perez		BA	UHealth Tower, Jackson Memorial	Miami, FL, USA	Data Coordinator	
Vivian	Calderon		BS	UHealth Tower, Jackson Memorial	Miami, FL, USA	Data Coordinator	
Rajan	Ramdev		BA	UHealth Tower, Jackson Memorial	Miami, FL, USA	Data Coordinator	
Aaliyah	Jolly		BS	UHealth Tower, Jackson Memorial	Miami, FL, USA	Data Coordinator	
Ivonne	Guzman		BA	UHealth Tower, Jackson Memorial	Miami, FL, USA	Data Coordinator	
Richard	Guerra		BS	UHealth Tower, Jackson Memorial	Miami, FL, USA	Data Coordinator	
Sebastian	Brito		BS	UHealth Tower, Jackson Memorial	Miami, FL, USA	Data Coordinator	
Rhonda	Hobbs		BS, MT(ASCP), SBB	UTHealth-Houston	Houston, TX, USA	Blood Bank	
Rebecca	Denham		MPH	UTHealth-Houston	Houston, TX, USA	Blood Bank	
John	Dick	II	MT, ASCP	UTHealth-Houston	Houston, TX, USA	Blood Bank	
Maria D	Hernandez		MS	UTHealth-Houston	Houston, TX, USA	Associate Site Coordinator	
Laura E	Nielsen		BSN, RN, CCRN	UTHealth-Houston	Houston, TX, USA	Research Nurse	
Sami M	Anjum		MS	UTHealth-Houston	Houston, TX, USA	Site Coordinator	
Shelby C	Mader		BS	UTHealth-Houston	Houston, TX, USA	Site Coordinator	
Taylor P	Stutz		BS	UTHealth-Houston	Houston, TX, USA	Site Coordinator	

Supplemental Online Content: Nonauthor Collaborators

*Indicates required information. Only first name, last name, and suffix will appear in PubMed.

*First Name and Middle Initial(s)	*Last Name	*Suffix (eg, Jr, III)	Academic Degrees	Institution	Location (city, state/province, country)	Role or Contribution, eg, chair, principal investigator	Group (if more than 1 Group listed in the byline) and/or Subgroup (eg, Steering Committee)
Mehriban	Mammadova		MPH	UTHealth-Houston	Houston, TX, USA	Site Coordinator	
Pamela	Nichols		MPH	UTHealth-Houston	Houston, TX, USA	Site Coordinator	
Tanya S	Khan		None	UTHealth-Houston	Houston, TX, USA	Site Coordinator	
Maha R	Boktour		MPH	UTHealth-Houston	Houston, TX, USA	Site Coordinator	
Brenda L	Castaneda		RMA	UT-RGV	Edinburg, TX, USA	Site Coordinator	
Brenda D	Benitez		None	UT-RGV	Edinburg, TX, USA	Site Coordinator	
Erik	Hinojosa		BA	UT-RGV	Edinburg, TX, USA	Site Coordinator	
Brenda C	Guerra		MLS	UT-RGV	Edinburg, TX, USA	Blood Bank	
Armando	Ortiz		MT	UT-RGV	Edinburg, TX, USA	Blood Bank	
Renee S	Hebbeler-Clark		MD	UT-Tyler	Tyler, TX, USA	Sub-Investigator	
Pamela J	McShane		MD	UT-Tyler	Tyler, TX, USA	Sub-Investigator	
Rebekah	Hibbard		BS, CCRC	UT-Tyler	Tyler, TX, USA	Site Project Manager	
Benji E	Hawkins		CCRC	UT-Tyler	Tyler, TX, USA	Site Coordinator	
Elizabeth R	Dohanich		BBA, CCRC	UT-Tyler	Tyler, TX, USA	Site Coordinator	
Carly	Wadle		MPH	UT-Tyler	Tyler, TX, USA	Site Coordinator	
Kimberly L	Greenlee		MPH	UT-Tyler	Tyler, TX, USA	Data Coordinator	
Jennifer	Brooks		RN	UT-Tyler	Tyler, TX, USA	Site Coordinator	
Christopher D	Herrick		BS, CCRC	UT-Tyler	Tyler, TX, USA	Regulatory Coordinator	
Amit	Gode		MBBS, MPH	Medical College of WI & Froedtert Hospital	Wauwatosa, Wisconsin	Site Co-PI	
Paul	Bergl		MD	Medical College of WI & Froedtert Hospital	Wauwatosa, Wisconsin	Site Co-PI	
Kurt	Hu		MD	Medical College of WI & Froedtert Hospital	Wauwatosa, Wisconsin	Site Co-PI	
Jayshil	Patel		MD	Medical College of WI & Froedtert Hospital	Wauwatosa, Wisconsin	Site Co-PI	
Shankar	Srinivasan		MS, MBA, PhD	Medical College of WI & Froedtert Hospital	Wauwatosa, Wisconsin	Site Project Manager	
Jeanette	Graf		BS	Medical College of WI & Froedtert Hospital	Wauwatosa, Wisconsin	Site Project Manager	
Char	Klis		none	Medical College of WI & Froedtert Hospital	Wauwatosa, Wisconsin	Site Project Manager	
Kyersten	Reimer		none	Medical College of WI & Froedtert Hospital	Wauwatosa, Wisconsin	Rand Key Holder	
Erica	Carpenter		BS	Medical College of WI & Froedtert Hospital	Wauwatosa, Wisconsin	Blood bank	
Christine	Naczek		BS	Medical College of WI & Froedtert Hospital	Wauwatosa, Wisconsin	Blood bank	
Rae	Petersen		BS	Medical College of WI & Froedtert Hospital	Wauwatosa, Wisconsin	Associate Site Coordinator	
Renee	Dex		BSN	Medical College of WI & Froedtert Hospital	Wauwatosa, Wisconsin	Research Nurse Manager	
Jennifer	Drossart		BS, MPH	Medical College of WI & Froedtert Hospital	Wauwatosa, Wisconsin	Associate Site Coordinator	
James	Zelten		MS	Medical College of WI & Froedtert Hospital	Wauwatosa, Wisconsin	Associate Site Coordinator	
Charles	Brummitt		MD	St. Luke’s Advocate Aurora Health	Milwaukee, Wisconsin	Site PI	
Mengyao	Liang		MD	St. Luke’s Advocate Aurora Health	Milwaukee, Wisconsin	Lead Sub-Investigator	
Lynda	Yanny		BSN	St. Luke’s Advocate Aurora Health	Milwaukee, Wisconsin	Research Nurse Manager	
Gary	Dennison		none	St. Luke’s Advocate Aurora Health	Milwaukee, Wisconsin	Regulatory Coordinator	
Phyllis	Runningen		BSN	St. Luke’s Advocate Aurora Health	Milwaukee, Wisconsin	Lead Site Coordinator	
Brian	Brzezinski		BS	St. Luke’s Advocate Aurora Health	Milwaukee, Wisconsin	Rand Key Holder	
Stephen	Fiebig		BS	St. Luke’s Advocate Aurora Health	Milwaukee, Wisconsin	Rand Key Holder	
Erica	Carpenter		BS	St. Luke’s Advocate Aurora Health	Milwaukee, Wisconsin	Blood Bank	
Chris	Naczek		BS	St. Luke’s Advocate Aurora Health	Milwaukee, Wisconsin	Blood Bank	
Michelle	Kasdorf		MS	St. Luke’s Advocate Aurora Health	Milwaukee, Wisconsin	Associate Site Coordinator	
