# Supplementary material for: Efficacy and Safety of COVID-19 Convalescent Plasma in Hospitalized Patients: A Randomized Clinical Trial
Source: JAMA Intern Med. 2021 Dec 13;182(2):1–12. doi: 10.1001/jamainternmed.2021.6850 (PMC8669605; doi:10.1001/jamainternmed.2021.6850)
Supplement: Supplement 5. — Data Sharing Statement [file jamainternmed-e216850-s005.pdf]

# Data Sharing Statement

Ortigoza. Efficacy and Safety of COVID-19 Convalescent Plasma in Hospitalized Patients. *JAMA Intern Med*. Published December 13, 2021. doi:10.1001/jamainternmed.2021.6850

**Data**
**Data available:** Yes
**Data types:** Deidentified participant data
**How to access data:** Send email to: CONTAINData@nyulangone.org
**When available:** beginning date: 01-31-2022

**Supporting Documents**
**Document types:** None

**Additional Information**
**Who can access the data:** Qualified researchers.
**Types of analyses:** Primary outcome. Secondary outcome.
**Mechanisms of data availability:** Data request with description of proposed research will be reviewed. If proposal is approved, data will be available after a signed data sharing agreement with NYU Langone is executed. Approval by investigators. Signed data access agreement.
